# The m^6^A reader ECT8 is an abiotic stress sensor that accelerates mRNA decay in Arabidopsis

**DOI:** 10.1093/plcell/koae149

**Published:** 2024-06-05

**Authors:** Zhihe Cai, Qian Tang, Peizhe Song, Enlin Tian, Junbo Yang, Guifang Jia

**Affiliations:** Synthetic and Functional Biomolecules Center, Beijing National Laboratory for Molecular Sciences, Key Laboratory of Bioorganic Chemistry and Molecular Engineering of Ministry of Education, College of Chemistry and Molecular Engineering, Peking University, Beijing 100871, China; Synthetic and Functional Biomolecules Center, Beijing National Laboratory for Molecular Sciences, Key Laboratory of Bioorganic Chemistry and Molecular Engineering of Ministry of Education, College of Chemistry and Molecular Engineering, Peking University, Beijing 100871, China; Synthetic and Functional Biomolecules Center, Beijing National Laboratory for Molecular Sciences, Key Laboratory of Bioorganic Chemistry and Molecular Engineering of Ministry of Education, College of Chemistry and Molecular Engineering, Peking University, Beijing 100871, China; Synthetic and Functional Biomolecules Center, Beijing National Laboratory for Molecular Sciences, Key Laboratory of Bioorganic Chemistry and Molecular Engineering of Ministry of Education, College of Chemistry and Molecular Engineering, Peking University, Beijing 100871, China; Synthetic and Functional Biomolecules Center, Beijing National Laboratory for Molecular Sciences, Key Laboratory of Bioorganic Chemistry and Molecular Engineering of Ministry of Education, College of Chemistry and Molecular Engineering, Peking University, Beijing 100871, China; Synthetic and Functional Biomolecules Center, Beijing National Laboratory for Molecular Sciences, Key Laboratory of Bioorganic Chemistry and Molecular Engineering of Ministry of Education, College of Chemistry and Molecular Engineering, Peking University, Beijing 100871, China; Peking-Tsinghua Center for Life Sciences, Peking University, Beijing 100871, China; Beijing Advanced Center of RNA Biology, Peking University, Beijing 100871, China

## Abstract

*N* ^6^-methyladenosine (m^6^A) is the most abundant mRNA modification and plays diverse roles in eukaryotes, including plants. It regulates various processes, including plant growth, development, and responses to external or internal stress responses. However, the mechanisms underlying how m^6^A is related to environmental stresses in both mammals and plants remain elusive. Here, we identified EVOLUTIONARILY CONSERVED C-TERMINAL REGION 8 (ECT8) as an m^6^A reader protein and showed that its m^6^A-binding capability is required for salt stress responses in Arabidopsis (*Arabidopsis thaliana*). ECT8 accelerates the degradation of its target transcripts through direct interaction with the decapping protein DECAPPING 5 within processing bodies. We observed a significant increase in the *ECT8* expression level under various environmental stresses. Using salt stress as a representative stressor, we found that the transcript and protein levels of ECT8 rise in response to salt stress. The increased abundance of ECT8 protein results in the enhanced binding capability to m^6^A-modified mRNAs, thereby accelerating their degradation, especially those of negative regulators of salt stress responses. Our results demonstrated that ECT8 acts as an abiotic stress sensor, facilitating mRNA decay, which is vital for maintaining transcriptome homeostasis and enhancing stress tolerance in plants. Our findings not only advance the understanding of epitranscriptomic gene regulation but also offer potential applications for breeding more resilient crops in the face of rapidly changing environmental conditions.

## Teaser

An m^6^A reader accelerates target mRNA degradation by directly interacting with a decapping protein within processing bodies.

## Introduction

As one of the most essential internal chemical modifications in eukaryotic mRNA, *N*^6^-methyladenosine (m^6^A) plays important roles in numerous processes, including chromatin maintenance, RNA processing and metabolism, translation efficiency (TE), as well as other biological events (Jia et al. 2011; Zheng et al. 2013; Liu et al. 2014; Ping et al. 2014; Wang et al. 2014, 2015; Xiao et al. 2016; Pendleton et al. 2017; Xu et al. 2021, 2022). The dynamic regulation of m^6^A modification involves the well-coordinated efforts of methyltransferases and demethylases and m^6^A-binding proteins that participate in entire RNA lifecycle (Jia et al. 2011; Zheng et al. 2013; Liu et al. 2014; Ping et al. 2014; Xiao et al. 2016; Pendleton et al. 2017; Knuckles et al. 2018; Yue et al. 2018; van Tran et al. 2019; Xu et al. 2021). In mammals, m^6^A modifications have been associated with a wide range of biological processes, including embryonic development, stem cell differentiation, and cancer progression (Zheng et al. 2013; Geula et al. 2015; Xu et al. 2017, 2021; Yoon et al. 2017; Yankova et al. 2021). However, the understanding of the precise mechanisms underlying how m^6^A influences transcription processes and subsequent fate determination in plants remains incomplete.

In Arabidopsis (*Arabidopsis thaliana*), disruption of the m^6^A writer core subunit leads to developmental defects and even embryonic lethality (Zhong et al. 2008; Bodi et al. 2012; Shen et al. 2016; Růžička et al. 2017; Sun et al. 2022; Wang et al. 2022; Zhang et al. 2022a). Conditional complementation of these mutants has unveiled the regulatory role of m^6^A in various aspects of plant development (Bodi et al. 2012; Wong et al. 2023). Two m^6^A demethylases in Arabidopsis, ALKB HOMOLOG 9B (ALKBH9B) and ALKBH10B, have been identified, and they are associated with stress response, floral transition, and viral infection (Duan et al. 2017; Martínez-Pérez et al. 2017, 2021; Tang et al. 2021, 2022). Arabidopsis has 13 YT521-B homology (YTH) domain-containing proteins predicted to function as m^6^A-binding proteins. Among them, EVOLUTIONARILY CONSERVED C-TERMINAL REGION 2, 3, and 4 (ECT2, ECT3, and ECT4, respectively) have been characterized as m^6^A readers, collectively contributing to the regulation of leaf morphogenesis and abscisic acid (ABA) response with genetic redundancy (Arribas-Hernández et al. 2018, 2020; Scutenaire et al. 2018; Wei et al. 2018; Song et al. 2023). Mechanistic studies have revealed that ECT2, ECT3, and ECT4 form a complex in the cytoplasm and interact with poly(A) binding proteins, POLY(A) BINDING PROTEIN 2 and 4 (PAB2 and PAB4), to enhance the stability of their bound m^6^A-modified mRNAs (Song et al. 2023). The longer isoform of CLEVAGE AND POLYADENYLATION SPECIFICITY FACTOR 30 (CPSF30-L) is another identified m^6^A-binding protein in Arabidopsis, which regulates floral transition, ABA response, and nitrogen signaling (Hou et al. 2021; Song et al. 2021). It recognizes m^6^A-modified far upstream elements (FUE) of polyadenylation signal, influencing poly(A) site selection within liquid-like nuclear bodies (Song et al. 2021).

Disrupting m^6^A writers or erasers in Arabidopsis has revealed that m^6^A also plays a role in mRNA degradation (Luo et al. 2014; Anderson et al. 2018; Govindan et al. 2022). However, whether other m^6^A readers participate in this regulatory process remains to be well-investigated. The research is necessary to elucidate the precise mechanism involving how m^6^A is related to mRNA degradation in plants.

The m^6^A modification is emerging as an epitranscriptomic mark that regulates gene expression, and it exhibits the potential to swiftly adapt to environmental stresses. When mammalian cells are subjected to heat shock or hypoxia, ∼5% to 10% of m^6^A sites in mRNAs undergo dynamic changes (Liu et al. 2023). Similarly, Arabidopsis also displays dynamic changes in m^6^A levels after 6 h treatment with 150 mM NaCl (Hu et al. 2021). Nevertheless, the mechanisms by which m^6^A responds to environmental stresses in both mammals and plants remain elusive. The regulation of m^6^A modification hinges on 3 key categories of proteins: the m^6^A writers, erasers, and readers, and it has been observed that the expression level of m^6^A writer subunits increases after high-concentration NaCl treatment (Hu et al. 2021). Nonetheless, the specific m^6^A reader proteins responsible for facilitating a rapid response to environmental stresses remain a mystery.

Considering that the detailed mechanism of most m^6^A reader proteins remains unclear and it could be an enormous work to investigate them individually, especially because of the potential functional redundancy observed before (Song et al. 2023), we initially focus on ECT8, which exhibits the strongest response to external abiotic stress. It suggests that ECT8 could be a promising point of entry for studying the function of m^6^A modification during rapid signaling transmission in Arabidopsis and it is worthy to explore whether ECT8 has other intriguing regulatory functions.

In this study, we discovered that ECT8, an m^6^A reader protein, serves as a sensor of abiotic stresses in Arabidopsis. ECT8 exhibits a strong binding affinity to m^6^A-modified mRNA through recognition with conserved tryptophan residues, and it primarily localizes within processing bodies (P-bodies) in the cytoplasm, where it exerts its regulatory functions. Combining analysis of formaldehyde crosslinking and immunoprecipitation (FA-CLIP)-sequencing, RNA-seq, and mRNA lifetime sequencing confirmed that ECT8 accelerates the degradation of targeted mRNAs. More specifically, ECT8 directly interacts with the decapping protein DECAPPING 5 (DCP5), contributing to the accelerated degradation of m^6^A-modified mRNA. Under salt stress, the increased abundance of ECT8 enhances its binding capability, thereby amplifying the degradation of ECT8-bound mRNAs. On the other hand, disruption of *ECT8* leads to increased expression levels of negative regulators of salt stress, resulting in the elevated sensitivity to salinity. Collectively, our findings demonstrate that ECT8 serves as an abiotic stress sensor by accelerating mRNA decay in Arabidopsis, which is important for transcriptome homeostasis maintenance and stress tolerance. This work provides valuable insights into the molecular mechanisms governing m^6^A modification and its role in plant stress responses, with broad implications for agriculture and environmental adaptation.

## Results

### ECT8 is an m^6^A-binding protein

**Figure 1.**
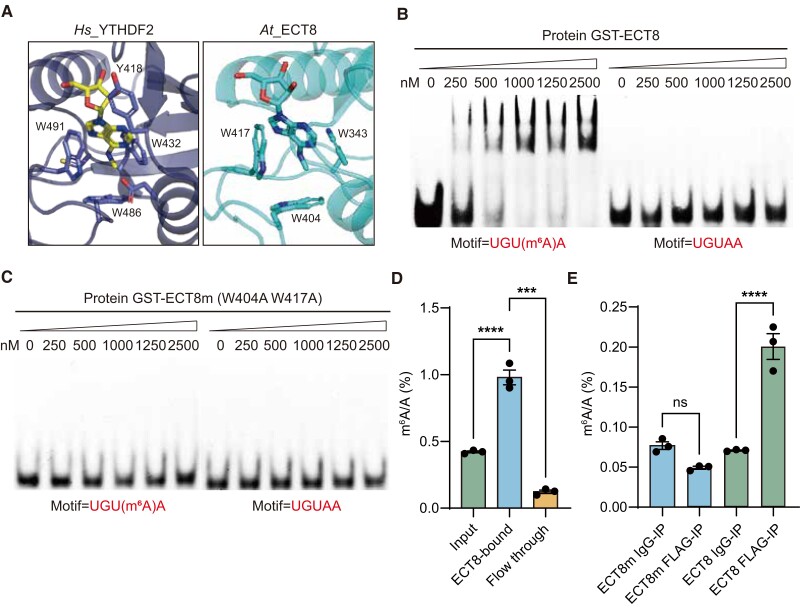
ECT8 is an m^6^A-binding protein in Arabidopsis. **A)** Structure of ECT8 simulated by AlphaFold (AF-Q9FPE7-F1) comparing with the published structure of YTH-YTHDF2 in complex with m^6^A (PDB: 4RDN). The ligands for binding m^6^A nucleotide are highlighted. **B)** EMSA assay showing the binding ability of GST-ECT8 with RNA probe containing m^6^A-modified UGUAA motif but not with unmethylated probe. Each lane was loaded with varying concentrations (shown below the triangle panel) of protein and a consistent amount of RNA oligo with a final concentration of 4 nM. **C)** EMSA assay showing abolished binding affinity of GST-ECT8m toward both methylated and unmethylated RNA probe with UGUAA motif. Each lane was loaded with varying concentrations (shown below the triangle panel) of protein and a consistent amount of RNA oligo with a final concentration of 4 nM. **D)** In vitro RIP-UPLC-MS/MS showing that m^6^A is enriched in ECT8-bound mRNA compared with input and the ﬂow-through fractions. Data are presented as means ± Se, *n* = 3 independent experiments, each with 3 technical replicates. ***P < 0.001 and ****P < 0.0001 by 1-way ANOVA. **E)** In vivo FA-RIP-UPLC-MS/MS showing that m^6^A is enriched in ECT8-Flag-bound RNA but not in ECT8m-Flag-bound RNA compared with IgG-bound RNA, separately. Data are presented as means ± Se, *n* = 3 independent experiments, each with 3 technical replicates. ns, not significant and ****P < 0.0001 by 1-way ANOVA.

To unravel the biological roles of ECT8 in Arabidopsis, we began with an extensive protein sequence alignment of the YTH domain, revealing high sequence similarities between ECT8 and YTH domain-containing family proteins (YTHDF1 to 3) in mammals, as well as a resemblance to the known Arabidopsis m^6^A reader protein, ECT2 (Supplementary Fig. S1A) (Scutenaire et al. 2018). Additionally, the sequence similarities between the YTH domain of ECT8 and YTHDF1 to 3 were 69.7%, 70.4%, and 69.7%, respectively, while the similarity to ECT2 was 81.7% (https://www.bioinformatics.org/). This alignment and structural analysis highlighted the importance of 3 conserved tryptophan residues (located at positions 343, 404, and 417 in ECT8) critical for m^6^A recognition within YTH domain, forming a hydrophobic aromatic cage similar to other YTH domain proteins, such as YTHDF2 (Fig. 1A).

In order to investigate ECT8’s ability to bind m^6^A modifications, we expressed and purified glutathione-*S*-transferase (GST)-tagged full-length wild-type (WT) ECT8 protein and a nonbinding variant (GST-ECT8m) with 2 mutations (W404A/W417A) (Supplementary Fig. S1B). Electrophoretic mobility shift assays (EMSA) with 5′-FAM-labeled RNA probes, both m^6^A-modified and unmodified, clearly demonstrated ECT8’s m^6^A-binding capability, a feature not observed with ECT8m (Fig. 1, B and C; Supplementary Fig. S1C). For further research, we conducted an in vitro RNA immunoprecipitation (RIP) assay coupled with ultrahigh-performance liquid chromatography and triple–quadrupole tandem MS (UPLC-MS/MS) using GST-ECT8 protein and poly(A)^+^ RNA extracted from Arabidopsis. Our findings showed a significant enrichment of m^6^A-modified poly(A)^+^ RNA within the fraction bound by GST-ECT8, in contrast to the flow-through fraction (Fig. 1D). These results confirm ECT8's recognition of m^6^A modifications in vitro and emphasize the critical role of conserved tryptophan residues in forming a hydrophobic aromatic cage that is essential for ECT8’s m^6^A-binding ability.

To further confirm that ECT8 indeed serves as an m^6^A-binding protein in planta, we conducted in vivo RIP assay coupled with UPLC-MS/MS using 2 native genetic complementation lines generated in the T-DNA insertion *ect8-1* mutant background: *ProECT8:ECT8-FLAG/ect8-1* (termed as *ECT8/ect8-1*) and *ProECT8:ECT8m-FLAG/ect8-1* (termed as *ECT8m/ect8-1*) transgenic plants (Supplementary Fig. S2, A to D). These lines, respectively, expressed WT ECT8 and the functionally impaired ECT8m (W404A/W417A) (Supplementary Fig. S2B). The results indicated that ECT8-FLAG-IP successfully isolated transcripts containing m^6^A modifications when compared with the IgG-IP control. On the other hand, both ECT8m-FLAG-IP and its corresponding IgG-IP control showed no such enrichment (Fig. 1E). These collective findings provide robust support for the conclusion that ECT8 functions as an m^6^A reader protein in Arabidopsis.

### *ECT8* is highly expressed in flowers, roots, and leaves, and ECT8 localizes in the cytoplasm

In our pursuit of uncovering the further molecular functions of ECT8, we explored its expression patterns in Arabidopsis. To achieve this, we generated *proECT8:GUS* transgenic plants and revealed that ECT8 is prominently expressed in vital anatomical regions, including flowers, roots, and leaves (Supplementary Fig. S3A). In roots, ECT8 displays specific expression patterns, with minimal presence in the meristematic zone but high expression levels in the root cap, mature zone, and elongation zone. Across other tissues, ECT8 primarily localizes to vascular tissues, while its expression diminishes notably in actively dividing areas like pollen and seeds, suggesting its role in responding to external stress in relatively mature tissues.

For a more comprehensive analysis of its tissue-specific expression pattern, we analyzed RNA-seq data from Arabidopsis RNA-seq database (Zhang et al. 2020) (http://ipf.sustech.edu.cn/pub/athrna/), which substantiated our findings from GUS staining assays (Supplementary Fig. S3B). We further fractionated nuclear and cytoplasmic portions using *ECT8/ect8-1* seedlings. Using histone H3 (H3) as a nuclear marker and UDP-glucose pyrophosphorylase (UGPase) as a cytoplasmic marker, we found the evidence that ECT8 is primarily localized in the cytoplasm (Supplementary Fig. S3C).

### Salt stress induces a significant increase in the transcript and protein abundance of ECT8

**Figure 2.**
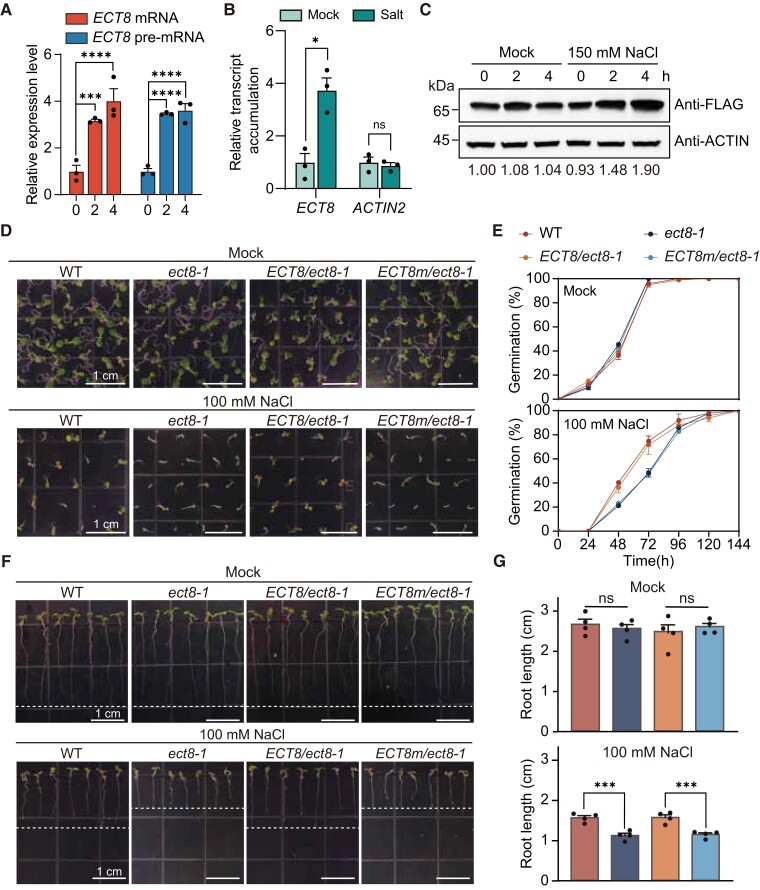
ECT8 is required for salt stress response in an m^6^A-dependent manner. **A)** RT-qPCR for the increase expression level of *ECT8* mRNA and unspliced immature transcripts (pre-mRNA) during salt stress over time. *TUB8* was used as negative control. Data are presented as means ± Se, *n* = 3 independent experiments, each with 3 technical replicates. ***P < 0.001, ****P < 0.0001 by 2-way ANOVA. **B)** Nuclear run-on assay indicating that the transcription rate of *ECT8* is highly increased after 4 h NaCl treatment. *ACTIN2* was used as negative control. Data are presented as means ± Se, *n* = 3 independent experiments, each with 3 technical replicates. ns, not significant, and *P < 0.05 by 2-way ANOVA. **C)** Protein immunoblot showing the relative expression level of ECT8 protein under mock and 150 mM salt treatment over time. ACTIN was used for loading control. kDa, kilodalton. **D)** Phenotypic analysis of salt response among WT, *ect8-1*, *ECT8/ect8-1*, and *ECT8m/ect8-1* plants under mock control and 100 mM NaCl treatments. Representative images showing the morphology of 7-d-old seedlings. WT, wild-type. The scale bar is shown as white lines. **E)** Statistical analysis of germination rates in WT, *ect8-1*, *ECT8/ect8-1*, and *ECT8m/ect8-1* plants under mock control and 100 mM NaCl treatment. WT, wild-type. Data are presented as means ± Se, *n* = 4 independent experiments, each with at least 35 seedlings. **F)** Phenotypic analysis of root length in WT, *ect8-1*, *ECT8/ect8-1*, and *ECT8m/ect8-1* plants under mock control and 100 mM NaCl treatments. The 3-d-old seedlings grown on 1/2 MS plates are transferred to regular 1/2 MS medium and medium supplemented with 100 mM NaCl and cultivated vertically, respectively. Representative images showing the morphology of 7-d-old seedlings. WT, wild-type. The scale bar is shown as white lines. **G)** Statistical analysis of primary root length in WT, *ect8-1*, *ECT8/ect8-1*, and *ECT8m/ect8-1* plants under mock control and 100 mM NaCl treatment. WT, wild-type. Data are presented as means ± Se, *n* = 4 independent experiments, each with at least 10 seedlings. ns, not significant, and ***P < 0.001 by 1-way ANOVA.

Analysis of an Arabidopsis RNA-seq database (Zhang et al. 2020) revealed that the expression level of *ECT8* is largely upregulated in response to various external abiotic stresses, including salt, oxidative, and drought stress, when compared with other YTH family proteins (Supplementary Fig. S4A). In contrast, *ECT8* expression remained relatively stable under conditions such as darkness-induced stress. To delve deeper into our investigation of ECT8's response to external abiotic stress, we subjected WT seedlings to a 150 mM NaCl treatment. The results showed a continuous increase in mRNA and pre-mRNA expression of *ECT8* over time during salt stress (Fig. 2A).

To further confirm that salt treatment increases *ECT8* transcription, we conducted a nuclear run-on assay coupled with RT-qPCR to measure the relative in situ transcription rate of *ECT8* in intact nuclei. The nuclear run-on assay was performed by supplying BrUTP to nuclei, and labeled transcripts were enriched by anti-BrdU beads. Indeed, we found the transcription accumulation of *ECT8* was significantly increased by four times upon a 4 h salt stress treatment compared with normal conditions (Fig. 2B). Subsequently, we also observed an increase in the protein level of ECT8 under salt stress (Fig. 2C). These findings show an enhanced transcription rate and higher ECT8 protein abundance under salt stress conditions. As for m^6^A writer subunits and erasers, in contrast to *ECT8*, no significant transcriptional changes were observed after the 4 h 150 mM NaCl treatment (Supplementary Fig. S4, B and C), suggesting that the m^6^A reader ECT8 is largely the main responder to salt stress instead of m^6^A writers, subunits, or erasers.

### ECT8's m^6^A-binding capability is required for salt stress response

Subsequently, we further investigated the role of ECT8 in responding to salt stress. Under normal conditions, both the *ect8-1* mutant and WT displayed indistinguishable germination and growth rates (Fig. 2, D and E). However, when exposed to varying high concentrations of NaCl (100 and 150 mM), *ect8-1* mutant plants exhibited delayed germination, reduced rates of green cotyledons and survival rate, and shorter primary root length compared with WT (Fig. 2, D to G; Supplementary Fig. S4, D to F). The hypersensitivity to salt stress in the *ect8-1* mutant can be rescued by expression of WT ECT8, but not by expression of ECT8m (Fig. 2, D to G; Supplementary Fig. S4, D to F). The results suggest that the m^6^A-binding ability of ECT8 regulates the response to salt stress.

### ECT8 binds to the 3′ untranslated region of mRNAs with m^6^A modification under both normal and salt stress conditions

**Figure 3.**
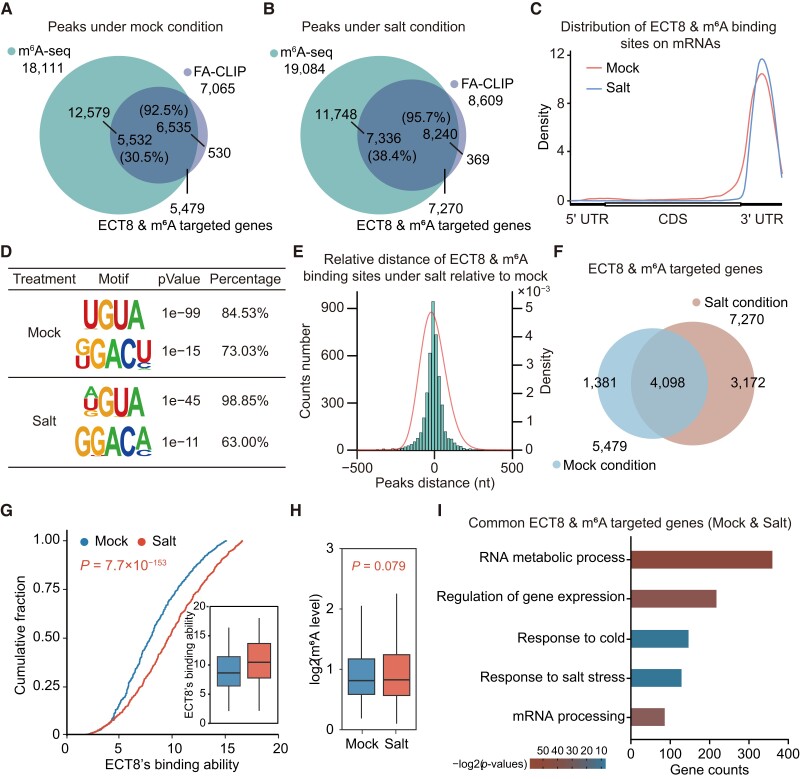
ECT8 binds to mRNA 3′UTR regions under normal and salt stress conditions. **A, B)** Overlap between FA-CLIP-identified ECT8-binding sites and m^6^A peaks under normal **A)** and salt stress conditions **B)**. **C)** Metagene proﬁle illustrating the region distribution of ECT8- and m^6^A-binding sites across the indicated mRNA segments under normal and salt stress conditions. 5′ UTR, 5′ untranslated region; CDS, coding sequence; 3′ UTR, 3′ untranslated region. **D)** Motifs identiﬁed by HOMER software based on the ECT8- and m^6^A-binding sites under normal and salt stress conditions. **E)** Distribution of the distance of ECT8- and m^6^A-binding sites under salt stress compared with those under normal condition. **F)** Venn plot depicting the overlap of ECT8- and m^6^A-targeted genes identified in normal and salt conditions. **G)** Cumulative plot combined boxplot showing the ECT8's binding ability toward 4,098 common ECT8- and m^6^A-targeted genes under normal and salt conditions. In boxplot, lower and upper hinges represent first and third quartiles, the center line represents the median, and whiskers represent ±1.5× interquartile range. *P*-values were calculated using Wilcoxon test. **H)** Boxplot indicating the m^6^A level of 4,098 common ECT8- and m^6^A-targeted genes under both conditions. Results have been calibrated with m^6^A spike-ins to diminish the difference of efficiency during immunoprecipitation in m^6^A-seq. Lower and upper hinges represent first and third quartiles, the center line represents the median, and whiskers represent ±1.5× interquartile range. *P*-values were calculated using Wilcoxon test. **I)** GO analysis of 4,098 common ECT8- and m^6^A-targeted genes identified in both normal and salt conditions. *P*-values were calculated from DAVID website (https://david.ncifcrf.gov/).

Considering that the function of m^6^A reader proteins relies heavily on their specific target genes, we conducted 2 biological replicated strand-specific FA-CLIP and 3 biological replicated strand-specific m^6^A-seq experiments under normal (mock) and 150 mM NaCl (salt) conditions to explore the molecular function of ECT8 (Supplementary Fig. S5, A and B). In the overlapping biological replicated sequencing results, we identified 18,111 m^6^A peaks (Supplementary Data Set 1) and 7,065 ECT8-binding sites under mock condition (Supplementary Data Set 3). Over 92% of ECT8’s binding sites overlapped with m^6^A peaks (6,535 ECT8- and m^6^A-binding sites), resulting in 5,479 identified ECT8- and m^6^A-targeted genes (Fig. 3A). Similarly, under salt condition, we identified 19,084 m^6^A peaks (Supplementary Data Set 2) and 8,609 ECT8-binding sites (Supplementary Data Set 4), revealing that over 95% of ECT8's binding sites harbor m^6^A peaks (8,240 ECT8- and m^6^A-binding sites) corresponding to 7,270 ECT8- and m^6^A-targeted genes (Fig. 3B). Taking the ECT8- and m^6^A-targeted genes into further analysis, we found that ECT8 predominantly interacts with mRNAs, with a concentrated binding distribution within the 3′ untranslated region (UTR) region under both conditions (Fig. 3C; Supplementary Fig. S5, C and D). The known m^6^A motifs found in plants, URUAY (R = A or G, Y = C or U), and RRACH (R = A or G, and H is not G) were also identified in ECT8's binding sites (Fig. 3D).

### Elevated ECT8 protein enhances its binding capacity to m^6^A-modified mRNAs under salt stress

To explore the regulatory function of ECT8 under salt stress, we subsequently investigated whether salt stress would alter ECT8's m^6^A-binding pattern and capability. We found that salt stress treatment did not disturb the m^6^A distribution pattern or ECT8's binding positions (Fig. 3E; Supplementary Fig. S5E). Remarkably, over 60% of ECT8 and m^6^A-binding sites were overlapped between normal and salt conditions, corresponding to 4,098 ECT8- and m^6^A-targeted genes (termed as common ECT8- and m^6^A-targeted genes) identified in both conditions (Fig. 3F; Supplementary Fig. S5, F and G). These indicate that ECT8 maintains a consistent m^6^A-binding pattern across both conditions.

We then used the common ECT8- and m^6^A-targeted genes identified in both normal and salt stress conditions for the evaluation of ECT8's binding capability under normal and salt condition. The results showed that ECT8 exhibits a significantly increased binding ability of common ECT8- and m^6^A-targeted genes under salt stress condition compared with normal condition, although overall m^6^A abundance does not differ between the conditions (Fig. 3, G and H; Supplementary Fig. S5H). It suggests that the elevated ECT8 protein level induced by salt stress enhances its interaction with targeted transcripts.

To gain a deeper understanding of ECT8's role in response to external stress, we conducted gene ontology (GO) pathway analysis using common ECT8- and m^6^A-targeted genes. It revealed a distinct enrichment of pathways related to RNA metabolism, gene expression regulation, and responses to salt and cold stresses (Fig. 3I). Additionally, we also performed the GO analysis for the unique ECT8- and m^6^A-targeted genes specific to normal and salt condition (Fig. 3F; Supplementary Fig. S5, I and J). Only the unique ECT8- and m^6^A-targeted genes specific to salt condition were involved in pathways related to osmotic changes and salt stress response. These results underscore the comprehensive role of ECT8 in regulating the expression levels of targeted transcripts under stresses.

### ECT8 accelerates the degradation of m^6^A-modified mRNAs

**Figure 4.**
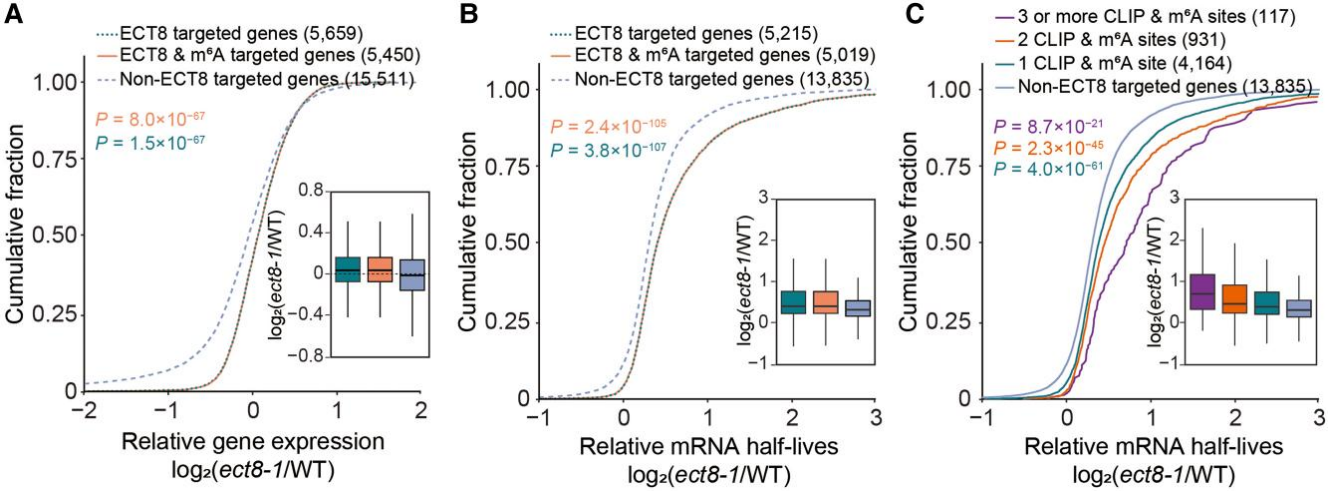
ECT8 facilitates the degradation of m^6^A-modified mRNAs**. A)** Cumulative distribution and boxplot of relative mRNA expression of 5,659 ECT8-targeted genes, 5,450 ECT8- and m^6^A-targeted genes, and 15,511 non-ECT8-targeted genes in *ect8-1* compared with WT under mock condition. In boxplot, lower and upper hinges represent first and third quartiles, the center line represents the median, and whiskers represent ±1.5× interquartile range. WT, wild-type. *P*-values were calculated using Wilcoxon test. **B)** Cumulative distribution and boxplot of relative mRNA half-lives of 5,215 ECT8-targeted genes, 5,019 ECT8- and m^6^A-targeted genes, and 13,835 non-ECT8-targeted genes in *ect8-1* compared with WT under mock conditions. In boxplot, lower and upper hinges represent first and third quartiles, the center line represents the median, and whiskers represent ±1.5× interquartile range. WT, wild-type. *P*-values were calculated using Wilcoxon test. **C)** Cumulative distribution and boxplot of relative mRNA half-lives of ECT8- and m^6^A-targeted genes with 3 or more binding sites (117), 2 binding sites (931), 1 binding site (4,164), and non-ECT8-targeted genes (13,835) in *ect8-1* compared with WT under mock conditions. In boxplot, lower and upper hinges represent first and third quartiles, the center line represents the median, and whiskers represent ±1.5× interquartile range. WT, wild-type. *P*-values were calculated using Wilcoxon test.

To investigate gene expression regulation mediated by ECT8, we performed strand-specific poly(A)^+^ RNA-seq on 12-d-old WT and *ect8-1* seedlings under normal condition, ensuring high replicability between the replicates (Supplementary Fig. S6A and Data Set 5). The identified transcripts were categorized into 3 groups: ECT8-targeted genes, ECT8- and m^6^A-targeted genes, and non-ECT8-targeted genes. Significantly, both ECT8-targeted genes and ECT8-and m^6^A-targeted genes exhibited higher transcript abundance than non-ECT8-targeted genes in *ect8-1* compared with WT (Fig. 4A). Given that changes in gene expression are predominantly influenced by transcription rates and RNA stability, coupled with ECT8's cytoplasmic localization (Supplementary Fig. S3C), it is reasonable to posit that ECT8 plays a pivotal role in promoting the degradation of its target transcripts.

Based on our investigation of ECT8, we performed transcriptome-wide mRNA lifetime sequencing on 7-d-old WT and *ect8-1* seedlings at different time points with high replicability (0, 15, 30, 60, and 120 min) (Supplementary Fig. S6B and Data Set 7). Our results showed that the transcripts of ECT8-targeted genes, as well as ECT8- and m^6^A-targeted genes, exhibited significantly longer half-lives in *ect8-1* when compared with non-ECT8-targeted genes (Fig. 4B). Further results revealed that mRNAs with more ECT8-binding sites exhibited notably longer half-lives than those with fewer binding sites in *ect8-1* compared with WT (Fig. 4C). It reinforces the hypothesis that ECT8 accelerates the degradation of its bound mRNAs, and this degradation process becomes more pronounced as the number of binding sites increases.

Considering the elevated ECT8 level under stress, we wonder whether there could be similar results under salt stress condition. Therefore, we performed strand-specific poly(A)^+^ RNA-seq on 12-d-old WT and *ect8-1* seedlings treated with 150 mM NaCl (Supplementary Fig. S6A and Data Set 6). It revealed that disruption of *ECT8* significantly increased mRNA abundance of ECT8-targeted genes and ECT8- and m^6^A-targeted genes compared with non-ECT8-targeted genes under salt condition (Supplementary Fig. S6C), consistent with the results under normal condition. These results further confirm that ECT8 promotes the degradation of its bound mRNAs under both normal and salt stress conditions.

Due to the cytoplasmic localization of ECT8, we explored its potential role in translation regulation. We first determined that ECT8 is exclusively localized in the nonribosome region (Supplementary Fig. S7A). Furthermore, ribo-seq results did not show significant differences in TE for non-ECT8-targeted genes, ECT8-targeted genes, and ECT8- and m^6^A-targeted genes between *ect8-1* mutant and WT under normal growth condition (Supplementary Fig. S7, B and C, and Data Set 8). These results confirm that ECT8 does not play a role in the overall translational regulation under normal condition.

### ECT8 promotes m^6^A-modified mRNA decay through direct interaction with DCP5 in P-bodies

**Figure 5.**
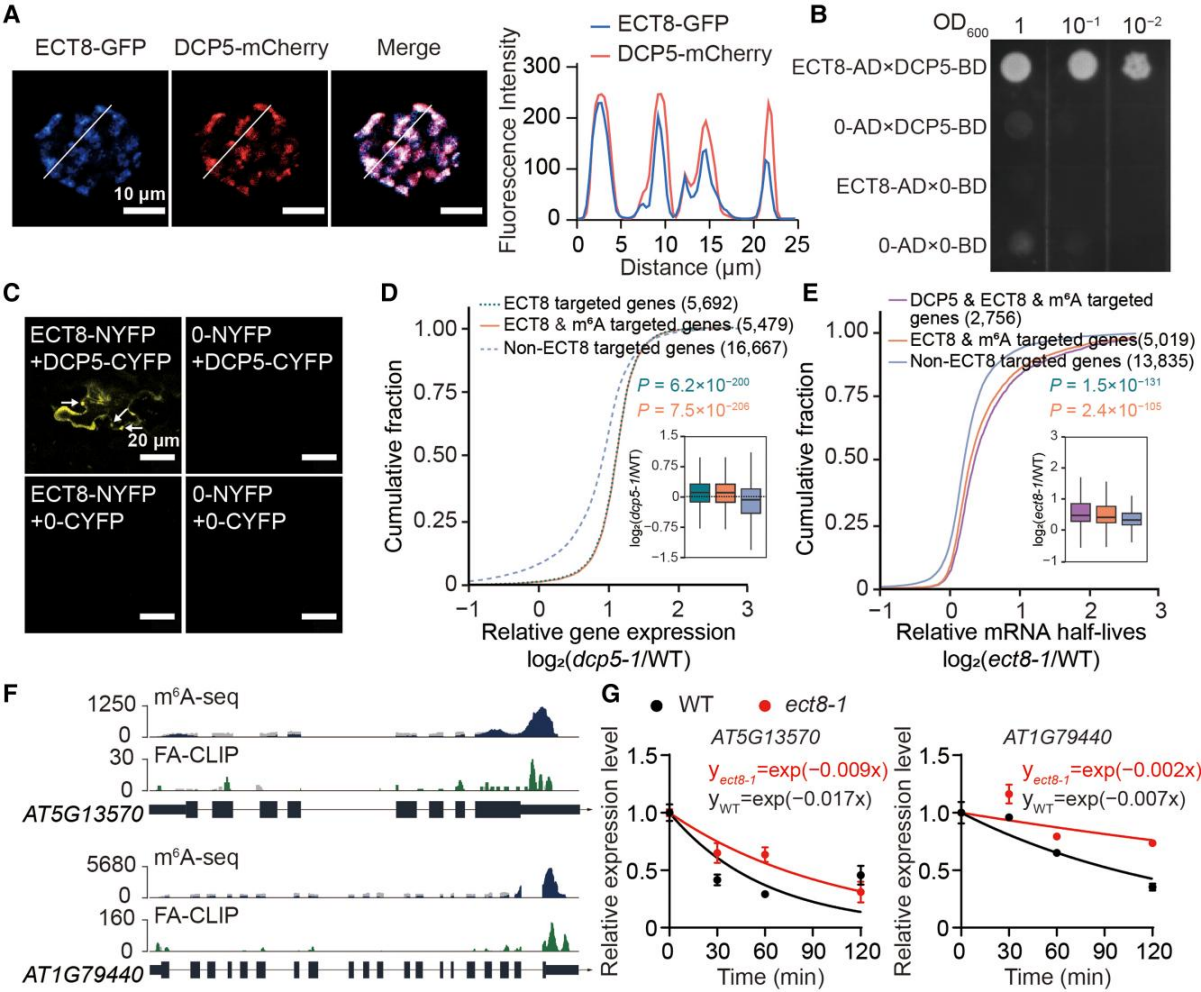
ECT8 accelerates the degradation decay of m^6^A-modified mRNA through direct interaction with DCP5 within P-bodies. **A)** Confocal microscopy showing the colocalization of ECT8-GFP and DCP5-mCherry in P-bodies from protoplast coexpression experiment. Intensity traces (white lines) are analyzed by ImageJ and plotted at the right. Scale bar = 10 *μ*m. **B)** Y2H assay showing the physical associations between ECT8 and DCP5 in yeast cells on selective medium without tryptophan, leucine, histidine, and adenine. The full-length CDS of ECT8 and DCP5 were fused with wither the GAL4-AD or BD domain as indicated. AD, the activation domain expressed from *pGADT7*; BD, the binding domain expressed from *pGBKT7*. 0-AD, the empty vector of *pGADT7*; 0-BD, the empty vector of *pGBKT7.***C)** BiFC assay showing the physical associations between ECT8 and DCP5 in *N. benthamiana* leaf cells. The puncta are highlighted using white arrows. Scale bars = 20 *μ*m. NYFP, N-terminal domain of YFP expressed from pBI121; CYFP, C-terminal domain of YFP expressed from pBI121; 0-NYFP, the empty vector of *pBI121-NYFP*; 0-CYFP, the empty vector of *pBI121-CYFP.***D)** Cumulative distribution and boxplot of relative expression level of 5,692 ECT8-targeted genes, 5,479 ECT8- and m^6^A-targeted genes, and 16,667 non-ECT8-targeted genes in *dcp5-1* compared with WT under mock conditions. In boxplot, lower and upper hinges represent first and third quartiles, the center line represents the median, and whiskers represent ±1.5× interquartile range. WT, wild-type. *P*-values were calculated using Wilcoxon test. **E)** Cumulative distribution and boxplot of relative mRNA half-lives of 2,756 DCP5-, ECT8-, and m^6^A-targeted genes, 5,019 ECT8- and m^6^A-targeted genes, and 13,835 non-ECT8-targeted genes in *ect8-1* compared with WT under normal condition. In boxplot, lower and upper hinges represent first and third quartiles, the center line represents the median, and whiskers represent ±1.5× interquartile range. WT, wild-type. *P*-values were calculated using Wilcoxon test. **F)** Integrative genomics viewer (IGV) showing the m^6^A-seq and FA-CLIP sequencing results on *AT5G13570* and *AT1G79440* transcripts. FA-CLIP, formaldehyde crosslinking and immunoprecipitation. **G)** The RNA half-lives of *AT5G13570* and *AT1G79440* transcripts in 7-d-old WT and *ect8-1* seedlings. External spike-ins were used as internal control. WT, wild-type. Data are presented as means ± Se, *n* = 2 independent experiments, each with 3 technical replicates.

In human, YTHDF2 localizes in P-bodies and directly interacts with CCR4–NOT Transcription Complex Subunit 1 (CNOT1), a component of the deadenylase complex (CCR4–NOT complex), to promote the deadenylation of m^6^A-containg RNAs (Du et al. 2016). Considering ECT8’s structural and functional similarities to YTHDF2, along with the presence of a disordered Prion-like domain (PrLD) at its N-terminus (Fig. 1A; Supplementary Fig. S8A), it implies that ECT8 could engage in liquid–liquid phase separation (LLPS) to exert its mRNA decay function. Our results showed that ECT8 and P-body component DECAPPING 1 (DCP1) and DCP5 colocalized in liquid puncta (Motomura et al. 2015) (Fig. 5A; Supplementary Fig. S8B), confirming that ECT8 is localized in P-bodies.

Given that ECT8 facilitates mRNA degradation and localizes in P-bodies, we suspected that proteins interacting with ECT8 might play a role in mRNA degradation. We conducted yeast 2-hybrid (Y2H) to screen several candidates responsible for 5′ cap structure removal or deadenylation. Eventually, DCP5 and VARICOSE (VCS) were identified as potential interacting components in P-bodies of ECT8, but not others such as DCP1, DECAPPING 2 (DCP2), a scaffold protein in the CCR4–NOT complex (NOT1), EXORIBONUCLEASE4 (XRN4), or even ARGONAUTE 1 (AGO1), which is involved in miRNA-mediated posttranscriptional gene silencing (Fig. 5B; Supplementary Fig. S8, C and D) (Vaucheret et al. 2004; Goeres et al. 2007; Xu et al. 2007; Maldonado-Bonilla 2014; Bhat et al. 2020; Pereira et al. 2020). DCP5, in conjunction with other proteins such as VCS, DCP1, and DCP2, primarily functions in removing the protective 5′ cap structure from mRNA molecules within P-bodies, ultimately leading to mRNA degradation by XRN4 (Xu and Chua 2009; Wawer et al. 2018). To validate this finding, we further conducted bimolecular fluorescence complementation (BiFC) by coexpressing ECT8 and DCP5 with split yellow fluorescent protein (YFP) in *Nicotiana benthamiana* leaves. The results demonstrated that the coexpression of ECT8 and DCP5 resulted in a strong reconstituted YFP signal in the cytoplasm (Fig. 5C), confirming the direct protein–protein interaction between ECT8 and DCP5.

Subsequently, we investigated whether DCP5 facilitates the function of ECT8 in mediating the degradation of m^6^A-modified mRNA. To achieve this, we analyzed published poly(A)^+^ RNA sequencing data from the *dcp5-1* mutant and its WT counterparts (GSE61812), categorizing the detected transcripts into 3 groups: ECT8-targeted genes, ECT8- and m^6^A-targeted genes, and non-ECT8-targeted genes. The analysis revealed that disruption of *DCP5* significantly increases the mRNA abundance of ECT8-targeted genes and ECT8- and m^6^A-targeted genes compared with non-ECT8-targeted genes (Fig. 5D). This suggests that DCP5 promotes the degradation of mRNAs bound by ECT8. Furthermore, we identified 10,776 genes that were upregulated in the *dcp5-1* mutant, which we refer to as DCP5-regulated genes. More than 60% of ECT8 and m^6^A target genes were overlapped with DCP5-regulated genes, collectively referred to as DCP5-, ECT8-, and m^6^A-targeted genes (Supplementary Fig. S8E). Our mRNA lifetime sequencing shows that DCP5-, ECT8-, and m^6^A-targeted genes had significantly prolonged mRNA half-lives in *ect8-1* (Fig. 5E), indicating a coregulatory function of ECT8 and DCP5 in mRNA degradation.

Moreover, we selected 4 DCP5-targeted genes, AT5G13570, AT1G79440, AT3G45970, and AT1G03440, for further confirmation. Among them, AT5G13570 and AT3G45970 have been validated (Xu and Chua 2009). The results from FA-CLIP and m^6^A-seq showed that the binding sites of ECT8 on transcripts of AT5G13570 and AT1G79440 contain m^6^A modifications, while the other 2 transcripts lack m^6^A modifications (Fig. 5F; Supplementary Fig. S8F). The mRNA lifetime assays revealed that the mRNA half-lives of AT5G13570 and AT1G79440 are longer in *ect8-1* than those in WT, but this difference is not observed in AT3G45970 and AT3G45970 (Fig. 5G; Supplementary Fig. S8G). These results collectively demonstrate that ECT8, leveraging its m^6^A-binding capability, promotes RNA decay through its interaction with DCP5 in P-bodies.

### ECT8 destabilizes negative regulators of salt stress response for enhancing salt stress tolerance

**Figure 6.**
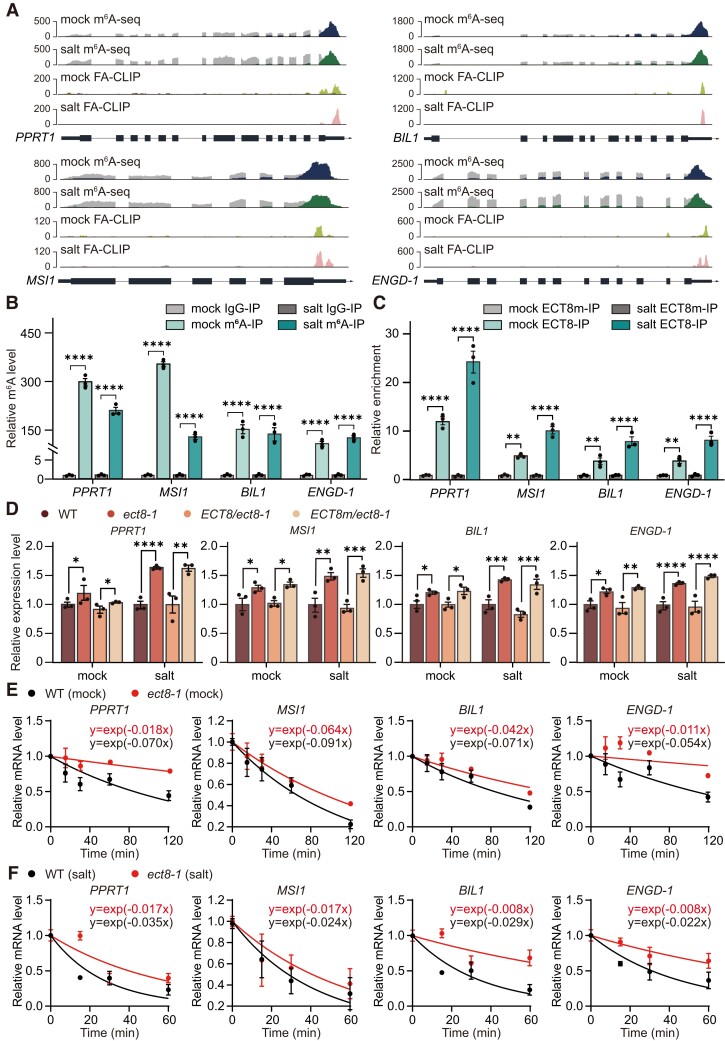
ECT8 amplifies the degradation of the negative salt stress regulators under salt stress condition. **A)** Integrative genomics viewer showing the sequencing results on *PPRT1*, *MSI1*, *BIL1*, and *ENGD-1* transcripts. FA-CLIP, formaldehyde crosslinking and immunoprecipitation. **B)** m^6^A-IP-qPCR validation of the m^6^A enrichment level in *PPRT1*, *MSI1*, *BIL1*, and *ENGD-1* in 12-d-old WT seedlings under mock and salt conditions. IgG-IP was used for negative control, and external m^6^A spike-in was used for calibration. IP, immunoprecipitation. Data are presented as means ± Se, *n* = 3 independent experiments, each with 3 technical replicates. ****P < 0.0001 by 2-way ANOVA. **C)** FA-RIP-qPCR validation of the binding affinity of ECT8 toward *PPRT1*, *MSI1*, *BIL1*, and *ENGD-1* in 12-d-old *ECT8/ect8-1* and *ECT8m/ect8-1* seedlings under both mock and salt stress conditions. *ECT8m/ect8-1* was used as negative control. *AT2G07689* was used as internal control. IP, immunoprecipitation. Data are presented as means ± Se, *n* = 3 independent experiments, each with 3 technical replicates. **P < 0.01 and ****P < 0.0001 by 2-way ANOVA. **D)** Relative mRNA expression levels of *PPRT1*, *MSI1*, *BIL1*, and *ENGD-1* in 12-d-old WT, *ect8-1*, *ECT8/ect8-1*, and *ECT8m/ect8-1* seedlings under mock and salt stress. *TUB8* was used as the internal control. Data are presented as means ± Se, *n* = 3 independent experiments, each with 3 technical replicates. *P < 0.05, **P < 0.01, ***P < 0.001, and ****P < 0.0001 by 2-way ANOVA. **E, F)** The mRNA half-lives of *PPRT1*, *MSI1*, *BIL1*, and *ENGD-1* in 7-d-old WT and *ect8-1* seedlings under mock **E)** and salt stress **F)** conditions. External spike-ins were used as internal control. Data are presented as means ± Se, *n* = 2 independent experiments, each with 3 technical replicates.

Building on the function of ECT8 that promotes m^6^A-modified mRNA decay through its interaction with DCP5 in P-bodies, we dedicated efforts into salt stress hypersensitivity that is observed in the *ect8-1* mutant (Fig. 2, D to G; Supplementary Fig. S4, D to F). Consistent with our observation that the common ECT8- and m^6^A-targeted genes are significantly enriched in the salt stress pathway (Fig. 3I), we also found that ECT8- and m^6^A-targeted genes, specifically those upregulated in *ect8-1* from poly(A)^+^ RNA sequencing data, are also enriched in the salt stress response pathway (Supplementary Fig. S9, A and B). Therefore, we selected 4 negative regulators of salt stress response: *PROTEIN WITH THE RING DOMAIN AND TMEMB_185A 1* (*PPRT1*), *ARABIDOPSIS MULTICOPY SUPPRESSOR OF IRA1* (*MSI1*), *BIN2-LIKE 1* (*BIL1*), and *GTPASE/GTP-BINDING PROTEIN* (*ENGD-1*) (Alexandre et al. 2009; Sui et al. 2019; Li et al. 2020; Liu et al. 2020). Notably, all these transcripts are targeted by ECT8 within their m^6^A regions, as illustrated in FA-CLIP and m^6^A-seq (Fig. 6A). Interestingly, we observed that the binding affinity of ECT8 on each transcript was much more enhanced under salt stress condition compared with normal condition, despite no significant change in m^6^A levels between these 2 conditions.

To confirm the results observed above, we performed m^6^A-IP-qPCR and FA-RIP-qPCR under normal and salt stress conditions. It showed that the transcripts of *PPRT1*, *MSI1*, *BIL1*, and *ENGD-1* were indeed modified with m^6^A and directly bound by ECT8 under both conditions (Fig. 6, B and C). Moreover, the expression levels of these 4 transcripts were elevated in the *ect8-1* mutant compared with WT, with a more pronounced increase observed under salt stress (Fig. 6D). These results align with the finding from our sequencing data that the elevated ECT8 protein levels induced by salt stress lead to elevated binding capability. The increased expression levels of *PPRT1*, *MSI1*, *BIL1*, and *ENGD-1* in the *ect8-1* mutant can be recovered by complementing with ECT8, but not with the m^6^A-binding impaired ECT8m (Fig. 6D). This provides evidence that the m^6^A-binding capacity of ECT8 is required for gene regulation and, therefore, the salt stress response.

In addition to our previous findings, we performed RT-qPCR analysis and observed no significant increase in the pre-mRNA levels of these targeted genes (Supplementary Fig. S10). Coupled with our discovery that ECT8 primarily localizes in the cytoplasm (Supplementary Fig. S3C), these results collectively suggest that ECT8 participates in the degradation process of these representative transcripts but not in the regulation of transcription. Considering that ECT8 promotes the degradation of m^6^A-modified mRNA, we performed mRNA lifetime assays to measure half-lives of these 4 negative regulators of salt stress response. The results showed that their transcripts underwent slower degradation in *ect8-1* compared with WT under both normal and salt stress conditions (Fig. 6, E and F).

**Figure 7.**
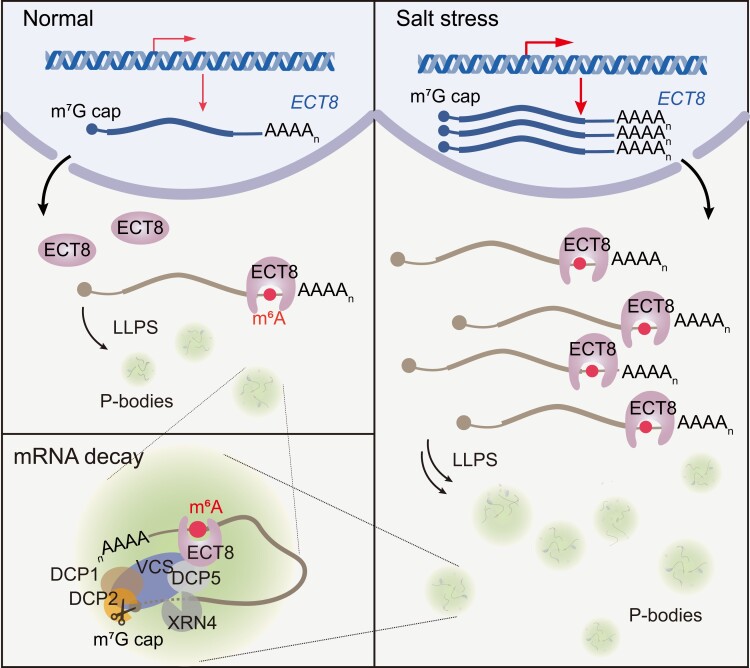
A model for ECT8 serves as a salt stress sensor in Arabidopsis. ECT8 functions as an abiotic stress sensor, promoting the degradation of targeted mRNAs in P-bodies by binding to m^6^A and interacting with the decapping protein DCP5. Using salt stress as an example, the transcription and expression levels of ECT8 are significantly increased. We hypothesize that the increased abundance of ECT8 induced by salt stress recruits more m^6^A-modified mRNAs to enter more P-bodies for the degradation of ECT8-bound mRNAs, including negative regulators of salt stress response, ultimately enhancing salt stress tolerance. LLPS, liquid–liquid phase separation; P-bodies, processing bodies.

Collectively, we demonstrated that ECT8 functions as a sensor for responding to abiotic stresses, facilitating the accelerated degradation of its bound m^6^A-modified mRNA through interacting and collaborating with DCP5 in P-bodies (Fig. 7). The abiotic stresses, including salt stress, result in an increase in the transcription and expression levels of *ECT8*. Considering that the number of P-bodies increases in Arabidopsis under salt stress condition (Steffens et al. 2015), we hypothesize that the increased abundance of ECT8, induced by salt stress, facilitates the recruitment of more m^6^A-modified mRNAs into P-bodies. As a consequence, this amplifies the degradation of ECT8-bound mRNAs, including negative regulators of salt stress response, ultimately enhancing salt stress tolerance.

## Discussion

Rapid climate changes and environmental stressors present great challenges to crop production. Epitranscriptomic modifications hold the promise of responding rapidly to environmental stresses, as evidenced by the alteration of the epitranscriptomic modification m^6^A in mammalian cells and plants under different stimuli (Zhou et al. 2015; Fu and Zhuang 2020; Hu et al. 2021; Govindan et al. 2022; Zhang et al. 2022b; Ries et al. 2023; Wang et al. 2023; Song et al. 2024). However, it remains unclear how m^6^A responds to these environmental stresses in detail. In this study, we identified ECT8 as an m^6^A reader in Arabidopsis and showed that it accelerates the degradation of m^6^A-modified mRNA by directly interacting with DCP5. Importantly, we discovered that ECT8 serves as a sensor for responding to abiotic stresses and enhances salt stress tolerance. Abiotic stresses lead to increased transcription of *ECT8* and result in a higher abundance of ECT8 protein. The increased abundance of ECT8 not only enhances its binding capability to a larger set of m^6^A-modified mRNAs but also amplifies the regulatory functions under abiotic stresses.

The regulation of m^6^A modification involves m^6^A writers, erasers, and reader proteins. While m^6^A modification holds the promise of responding rapidly to environmental stresses, there has been no conclusive evidence or findings indicating which m^6^A regulatory protein facilitates a quick response to these stresses. Theoretically, the m^6^A reader-mediated pathway is the fastest way for m^6^A to respond to environmental stress, as only m^6^A readers sense the stimuli and control RNA fate. In contrast, the m^6^A writer or eraser-meditated pathway involves 3 steps: the m^6^A writer or eraser senses stimuli and adds or removes m^6^A, and then, m^6^A is recognized by m^6^A reader proteins to control RNA fate. It was indeed observed that the expression levels of m^6^A writer subunits increased by about 50% after 6 h of exposure to 150 mM NaCl treatment, while the total m^6^A level in mRNA significantly increased after 12 h of treatment with 150 mM NaCl (Hu et al. 2021). This indicates that m^6^A alteration takes a longer time to respond to salt stress. In contrast, we found that both m^6^A writers and m^6^A modifications remained unchanged at 4 h of exposure to 150 mM NaCl treatment, whereas the expression level of *ECT8* increased by 3-fold after 2 h of treatment with 150 mM NaCl (Fig. 2A; Supplementary Fig. S4C). These results support our hypothesis that plants naturally employ the m^6^A reader-mediated pathway as the initial step to quickly respond to environmental stresses.

We have demonstrated that ECT8 rapidly responds to stress and amplifies the degradation of its bound m^6^A-modified mRNAs to regulate stress tolerance. However, the mechanism underlying how abiotic stresses induce increased transcription of *ECT8* remains unclear. We suspect that abiotic stresses might manipulate the transcription activity of transcription factors through posttranslation modifications. Further investigation is required to understand the mechanism by which the transcription of *ECT8* senses abiotic stresses.

Disrupting m^6^A writers or erasers revealed that m^6^A plays a role in mRNA degradation in plants (Duan et al. 2017; Wang et al. 2022). However, the precise mechanism by which m^6^A regulates mRNA degradation remains elusive. We discovered that Arabidopsis ECT8, as an m^6^A reader, accelerates the degradation of its bound m^6^A-modified mRNA through its direct interaction with DCP5 within P-bodies (Fig. 7). DCP5 facilitates the decapping of ECT8-bound m^6^A-modified mRNAs, followed by 5′-to-3′ exoribonucleolytic cleavage (Xu and Chua 2009). We confirmed that disruption of *DCP5* inhibits the degradation of ECT8-bound m^6^A-modified mRNA (Fig. 5, D and E). Human m^6^A reader protein YTHDF2 promotes mRNA degradation through 3 pathways: (i) YTHDF2 directly interacts with CNOT1, a component of the deadenylase complex (CCR4–NOT complex), for deadenylation (Zheng et al. 2008; Du et al. 2016); (ii) YTHDF2 interacts with HRSP12 to recruit endoribonuclease RNase P/MRP complex for internal cleavage (Park et al. 2019); and (iii) YTHDF2 interacts with UPF1 to recruit a decapping-promoting factor PNRC2 for decapping (Boo et al. 2022). In contrast to human YTHDF2, ECT8 does not directly interact with NOT1, the components of the deadenylase complex (CCR4–NOT complex) in Arabidopsis (Supplementary Fig. S8A), indicating ECT8 may not follow the deadenylation pathway for mRNA decay (Pereira et al. 2020). Our results also showed that 60% of ECT8- and m^6^A-targeted genes overlap with DCP5-regulated genes (Supplementary Fig. S8E), suggesting that other proteins, such as VCS (Supplementary Fig. S8D), might interact with ECT8 and facilitate ECT8-mediated m^6^A-modified mRNA decay.

It was previously observed that the number of P-bodies increases in Arabidopsis under salt stress condition (Steffens et al. 2015). We hypothesize that the increased abundance of ECT8 induced by salt stress recruits more m^6^A-modified mRNAs to enter more P-bodies for the degradation of ECT8-bound mRNAs. During the revision process, it was reported that under high-concentration ABA treatment, ECT8 translocates to stress granules (SGs) to suppress protein translation (Wu et al. 2024). Additionally, thus study showed that ECT8 undergoes LLPS under salt stress. As well as the similarity of the formation events of P-bodies and SGs, certain components of P-bodies can dynamically shuttle with SGs under stress conditions (Kearly et al. 2024), indicating the coexistence of P-bodies and SGs under stress, such as salt stress. These findings suggest that ECT8 might have dual functions: promoting mRNA degradation in P-bodies and halting mRNA translation in SGs under stress.

Analysis of the evolutionary tree of the YTH family proteins in plants has revealed that ECT8 shares the same evolutionary branch with ECT6 and ECT7 (Yin et al. 2021), suggesting potential functional redundancy among them. ECT8, along with the characterized m^6^A readers ECT2/3/4, localizes in the cytoplasm. In contrast to ECT8's function, the function of ECT2/3/4 promotes the stabilization of m^6^A-modified mRNA (Song et al. 2023). We postulate that ECT8 and ECT2/3/4 may engage in antagonistic regulatory functions, collectively participating in maintaining transcript homeostasis.

In summary, our study demonstrated that (i) ECT8 functions as an m^6^A reader and accelerates the degradation of its bound m^6^A-modified mRNA through a direct interaction with DCP5, leading to decapping, and (ii) ECT8 serves as a rapid sensor of stress responses and the increased abundance of ECT8 protein amplifies the degradation of m^6^A-modified mRNA under environmental stresses. These findings uncover how plants utilize the m^6^A reader-mediated pathway to swiftly respond to stresses, offering potential opportunities for breeding more resilient crops.

## Materials and methods

### Plant materials and cultivation condition

Arabidopsis (*A. thaliana*) T-DNA insertion mutant *ect8-1* (SALK_206710) with Col-0 ecotype background was obtained from the Arabidopsis Biological Resource Center (ABRC). All seeds were sterilized and kept at 4 °C in darkness for 3 d then were grown on half-strength MS (1/2 MS) medium for 12 d before being transferred to greenhouse under same condition (22 °C, 16 h light/8 h dark using cool-white fluorescent tubes) with a light intensity by region from 90 to 120 *μ*mol m^−2^ s^−1^. *N. benthamiana* was grown under the same lighting conditions at a temperature of 28 °C for 4 wks. In instances where the plants were subjected to transformation experiments, the apical meristem was pinched to induce the formation of larger leaves.

### Plasmid and transgenic plant constructs

To construct *proECT8:ECT8-FLAG* for transgenic plant transformation, the *ECT8* expression cassette, including the 2 kb 5′ upstream sequence and the entire ECT8 open reading frame without the stop codon, was amplified and cloned into the *pCAMBIA1305-3 × FLAG* vector. For *proECT8:ECT8m-FLAG* for transgenic plant transformation, the mutation was constructed using Mut Express MultiS Fast Mutagenesis kit V2 (C215, Vazyme). In detail, *ECT8m* was generated by mutagenesis PCR to change the 2 tryptophan (W)-encoding TGG sequences into alanine (A)-encoding GCC sequences. To construct *proECT8:GUS*, the 2 kb promoter region of *ECT8* was cloned into *pCAMBIA1305-GUS*. All of the *Agrobacterium tumefaciens*-mediated genetic transformation used the floral dip method. To construct *GST-ECT8* and *GST-ECT8m* for recombinant protein purification, the full-length or mutated coding sequence (CDS) of *ECT8* was cloned into *pGEX-6P-1*. To construct the plasmids used for Y2H bait expression, all the CDS of *ECT8*, *NOT1*, *DCP1*, *DCP2*, *DCP5*, *VCS*, *XRN4*, and *AGO1* without the start codon were cloned into *pGADT7* and *pGBKT7*. To construct the *35S:ECT8-GFP*, *35S:DCP1-mCherry*, and *35S:DCP5-mCherry* for PEG-mediated Arabidopsis protoplast transfection, CDS without the stop codon was cloned into *pBSK-GFP* and *pBSK-mCherry*, respectively. To construct the *35S:ECT8-NYFP* and *35S:DCP5-CYFP* for BiFC assay, the entire coding region without the stop codon was cloned into *pBI121-NYFP* or *pBI121-CYFP*, respectively. All these coding region were amplified from complementary DNA (cDNA), which was transcribed from WT poly(A)^+^ RNA using the PrimeScript RT Reagent Kit with gDNA Eraser (Perfect Real Time) (RR047A) from TaKaRa. All primers used are listed in Supplementary Data Set 9.

### Protein expression and purification

The plasmids for protein expression were transformed into *Escherichia coli* (*E. coli*) BL21 Gold (DE3) competent cells. Single colonies from the transformation were picked and cultured in 3 to 5 mL LB liquid medium containing 50 *μ*g/mL ampicillin at 37 °C and 220 rpm on a shaker for about 12 h. After that, all *E. coli* culture was then inoculated into 1 L LB liquid medium with 50 *μ*g/mL ampicillin and incubated at 37 °C and 200 rpm on a shaker. After about 1 h and a half, the optical density at 600 nm (OD_600_) was monitored until it reached a range of 0.6 to 0.8. The temperature was then reduced to 16 °C, and isopropyl-*β*-D-thiogalactoside (IPTG) was added to a final concentration of 0.5 mM to induce protein expression overnight. The following day, the *E. coli* cells were harvested and resuspended in 30 mL lysis buffer (10 mM Tris-HCl, pH 8.0, 500 mM NaCl, 1 mM PMSF, 3 mM DTT, and 5% [*v*/*v*] glycerol) followed by sonication in an ice bath for 20 to 30 min (20 W power, with 10 s pulses and 10 s pauses). After centrifugation, the supernatant was filtered through a 0.45 *μ*m membrane to obtain the lysis fraction. This fraction was loaded into a GST affinity column (GE Healthcare) pre-equilibrated. The protein was eluted using elution buffer (10 mM Tris-HCl, pH 8.0, 500 mM NaCl, 10 mM reduced glutathione, and 3 mM DTT), and solution was collected. The protein solution was then concentrated to a volume of 2 to 5 mL and further purified using Superdex 75 exclusion column (GE Healthcare). All the fractions collected were then concentrated to a suitable volume and protein concentration was quantified using the BCA method. Finally, glycerol was added to 25% (*v*/*v*) of the final volume of the protein solution, thoroughly mixed, aliquoted based on the desired volume, flash-frozen in liquid nitrogen, and stored at −80 °C to maintain protein activity.

### EMSA

The precise concentrations of the recombinant proteins GST-ECT8 and GST-ECT8m (W404A/W417A) were determined using the BCA Protein Quantification Kit (Vazyme). Proteins were diluted into a range of concentrations (25, 12.5, 10, 5, and 2.5 *μ*M) using 1× binding buffer (10 mM HEPES, pH 8.0, 50 mM KCl, 1 mM EDTA, 0.05% [*v*/*v*] Triton X-100, 5% [*v*/*v*] glycerol, and 10 mg/mL Salmon DNA [ZOMANBIO], 1 mM DTT, 40 U/mL RNase inhibitor [Thermo Fisher Scientific]). The EMSA reaction system was prepared by adding 1 *μ*L FAM-labeled RNA probe (4 nM as final concentration), 1 *μ*L recombinant protein for different concentration, 2 *μ*L 5× binding buffer, and 6 *μ*L nuclease-free water. After electrophoresis for 60 min at 90 V, samples with RNA loading buffer (250 mM Tris-HCl, pH 8.0, and 40% [*v*/*v*] glycerol) were loaded to Novex 4% to 20% (*w*/*v*) TBE gel (Thermo Fisher Scientific) in prechilled 0.5× TBE buffer (5 g/L Tris, 2.75 g/L boric acid, and 50 mM EDTA, pH 8.0) for electrophoresis separation for additional 90 min at 90 V in an ice bath. The results were visualized by ChemiDoc (Bio-Rad). All oligonucleotides used are listed in Supplementary Data Set 6.

### m^6^A quantification analysis by UPLC-MS/MS

One hundred to 200 ng of RNA was digested using 1 U of Nuclease P1 (Wako) in a 17 *μ*L volume, which included 10 mM NH_4_Ac (pH 5.3), at 42 °C for 4 h. Subsequently, 1 U of shrimp alkaline phosphatase (NEB) and 2 *μ*L of rCutsmart buffer (NEB) were added for an additional 4 h incubation at 37 °C. The digested nucleotides were then centrifuged at 21,000 × *g* for 30 min, and supernatant was extracted prior to sample injection. The nucleotides underwent separation through reverse phase ultra-performance liquid chromatography (Shimadzu) using a ZORBAX SB-Aq column (Agilent) and were detected by 5,500 triple–quadrupole mass spectrometer (AB SCIEX) operating in positive electrospray ionization mode with Multiple Reaction Monitoring (MRM) feature. The quantification of nucleosides was carried out by comparing the nucleoside's mass transitions to their respective base ions: *m/z* 282.0 to 150.1 (m^6^A), *m/z* 268.0 to 136.0 (A), *m/z* 245.0 to 113.1 (U), *m/z* 244.0 to 112.0 (C), and *m/z* 284.0 to 152.0 (G). Quantification was analyzed by referencing a standard curve created from a series of pure nucleoside standards (Sigma-Aldrich). The ratios of m^6^A to 4 kinds of nucleosides (A, U, C, and G) were then calculated using the fitting curve above.

### In vitro RIP-UPLC-MS/MS

Total RNA was extracted from 12-d-old seedlings using TRIzol Reagent (Invitrogen). Subsequently, ∼75 *μ*g of this total RNA was employed to isolate poly(A)^+^ RNA using oligo(dT)_25_ Dynabeads (Thermo Fisher Scientific) following the manufacturer's procedure. The poly(A)^+^ RNA (0.8 *μ*g, while an additional 0.2 *μ*g poly(A)^+^ RNA is referred to as the Input fraction) and recombinant proteins with a GST-Tag (final concentration 500 nM) were incubated at 4 °C in 200 *μ*L of IPP buffer (150 mM NaCl, 0.1% [*v*/*v*] NP-40, 10 mM Tris-HCl, pH 7.5, 4 U/μL RNase inhibitor [Thermo Fisher Scientific], and 0.5 mM DTT) for 2 h with gentle rotation. Pre-equilibrated GST-Tag affinity magnetic beads (Pierce) were added, followed by an additional 2 h incubation at 4 °C. The supernatant was collected, and the RNA was extracted through precipitation using ethanol, referred to as the flow-through fraction. Subsequently, the GST-Tag beads were washed 3 times with 200 *μ*L of IPP buffer, and RNA bound to the beads was extracted using TRIzol Reagent (Invitrogen) and recovered through precipitation using isopropanol, referred to as the protein-bound fraction. The abundance of m^6^A in the input, flow-through, and protein-bound fractions was measured using UPLC-MS/MS. All oligonucleotides used are listed in Supplementary Data Set 6.

### GUS staining assay

The homozygous *ProECT8:GUS* transgenic plants were planted following previous procedures. During the growth of Arabidopsis, the required tissue samples were collected and subjected to GUS staining (Coolaber, SL7160) for incubation at 37 °C overnight. Samples were washed with 75% (*v*/*v*) ethanol until the negative control (such as WT) appeared colorless, and observations were made using a stereomicroscope.

### Separation of nuclear and cytoplasmic fractions

One gram of 12-d-old Arabidopsis WT seedlings grown on 1/2 MS medium was collected, flash-frozen in liquid nitrogen, and subjected to grinding using a TissueLyser II (Qiagen) at 30 Hz for 1 min and 45 s. The resulting powdered samples were mixed with 10 mL of Honda buffer (comprising 440 mM sucrose, 1.25% [*v*/*v*] Ficoll, 2.5% [*w*/*v*] Dextran T40, 20 mM HEPES, pH 7.4, 10 mM MgCl_2_, 0.5% [*v*/*v*] Triton X-100, 1 mM DTT, and 1× protease inhibitor cocktail [Roche]) and rotated at 4 °C until the samples were completely homogenized (about 20 min). After filtering the samples twice through a single layer of Miracloth, a total of 300 *μ*L of the sample was retained as the total RNA component, and 50 *μ*L of the sample was preserved as the total protein component. The remaining sample was centrifuged at 4 °C and 2,000 × *g* for 5 min, and 400 *μ*L of the supernatant was collected as the cytoplasmic RNA component, with 50 *μ*L of the sample preserved as the cytoplasmic protein component. To completely remove interference from nuclear components, 4 *μ*L of RNase inhibitor (Thermo Fisher Scientific) was added to the 400 *μ*L cytoplasmic RNA component, followed by centrifugation at 4 °C and 14,000 × *g* for 15 min. The pellet from the previous centrifugation step was resuspended in 3 to 5 mL of Honda buffer and subjected to centrifugation at 4 °C and 2,000 × *g* for 5 min, repeated 3 or more times, to completely eliminate any remaining cytoplasmic components. The final pellet was resuspended in 1 mL of Honda buffer and centrifuged at 4 °C and 8,000 × *g* for additional 1 min. The pellet collected in this step was named as nuclear RNA component as well as a portion should also be reserved for the nuclear protein fraction.

### Salt stress phenotypic analysis and high-concentration NaCl treatment

Plants of WT and other different genotypes plants were cultivated under identical conditions as described before, and their seeds were collected at the same time. The mature seeds were carefully dried and stored at room temperature in darkness. The salt stress phenotypic experiments were conducted with a minimum of 3 repetitions, each consisting of 4 biological replicates (with no less than 35 seeds per genotype). As for the detail, using 1/2 MS culture medium containing varying NaCl concentrations (0, 100, 150 mM), we periodically assessed the germination rate, using the appearance of the radicle as the criterion, and the rate of green cotyledons.

For root length measurements, we used ∼10 seedlings per replicate. This procedure was replicated 3 times with 3-d-old seedlings. Initially, these seedlings were grown on regular 1/2 MS medium. They were then transferred to 1/2 MS medium containing either no NaCl, 100 mM NaCl, or 150 mM NaCl. They were then cultivated vertically for 4 or 5 d, and their root lengths were meticulously recorded from ImageJ software (version 1.54d).

The high-concentration NaCl treatment procedure was as follows: a specific number of 12-d-old seedlings were placed in liquid 1/2 MS culture medium with 150 mM NaCl (salt condition) and in liquid 1/2 MS culture medium without NaCl (mock condition) for all kinds of seedlings needed. After a 4 h treatment, the liquid medium was promptly blotted dry with blotting paper and all the samples were stored by freezing quickly in liquid N_2_ then stored at −80 °C.

### RT-qPCR

Total RNA of 12-d-old or 7-d-old seedlings was extracted using TRIzol Reagent (Invitrogen). RNA was subjected to reverse transcription using the PrimeScript RT Reagent Kit with gDNA Eraser (Perfect Real Time) (RR047A) from TaKaRa. The procedure involved an initial step of gRNA erasure, followed by reverse transcription. Subsequently, the transcribed cDNAs were appropriately diluted and employed as templates for PCR reactions with Hieff qPCR SYBR Green Master Mix (Low Rox) (Yeasen). These PCR reactions were then analyzed using the ViiATM7 instrument (Applied Biosystems) following the provided instructions. To ensure result accuracy, *TUB8*, *ACTIN2*, or other appropriate internal controls, such as the external spike-ins (*GLuc* and *CLuc*) for mRNA stability assay, were utilized to normalize the results, and each independent sample included 3 biological replicates and 3 technical replicates, with the exception of the RNA stability assay, which included 2 biological replicates. All primers used are listed in Supplementary Data Set 6.

### Y2H assay

The purified recombinant pGBKT7 or pGADT7 plasmid described before was cotransfected into yeast (*Saccharomyces cerevisiae*) AH109 chemically competent cells (ZOMANBIO, ZC1604) following the manufacturer’s instructions. Transformed cells were cultured on double dropout medium YSD-Leu-Trp, and protein interactions were evaluated on quadruple dropout medium YSD-Leu-Trp-Ade-His under 28 °C for 2 d.

### BiFC assay

The resulting construct pBI121-DCP5-CYFP and pBI121-ECT8-NYFP was introduced into *A. tumefaciens* GV3101 (pSoup-p19) chemically competent cell (ZOMANBIO, ZC1410). GV3101 containing plasmids above were coinfiltrated into 4-wk-old *N. benthamiana* leaves (p19 was employed to suppress transgenic silencing). The infiltrated leaves were initially incubated in darkness at 28 °C for 24 to 48 h. Subsequently, imaging was performed using an LSM700 AxioObserver (Zeiss) confocal laser scanning microscope with a Plan-Apochromat 20×/0.8 objective. Specifically, a 488 nm wavelength laser was utilized to excite the YFP, with the emission signal collected from 500 to 550 nm. All images were collected under a pinhole setting of 1.0 AU with a 2.0% laser intensity and digital gain of 1.0.

### Protoplast transient expression

Healthy leaves were selected and cut into fine shreds of ∼1 mm in size. These shreds were transferred into a culture dish containing the 10 mL digestion buffer (12.5 mg/mL Cellulase R10 (Yakult), 3 mg/mL Macerozyme R10 (Yakult), 400 mM mannitol, 20 mM KCl, 20 mM MES, pH 5.7, 10 mM CaCl_2_, 10 mg BSA, and 1× protease inhibitor [Roche]) and vacuum infiltrate in the dark for 15 min. Culture dish with the shredded leaves were put on a horizontal shaker at 22 °C for slow and light-protected enzymatic digestion, lasting ∼4 h. The digested solution was filtered through a Miracloth filter, followed by centrifugation for 3 min at 100 *g* to collect protoplast cells. The supernatant was removed and 20 mL of W5 solution (154 mM NaCl, 125 mM CaCl_2_, 5 mM KCl, and 2 mM MES, pH 5.7) was added to resuspend the cells and repeat again. After that, 200 *μ*L of prechilled MMg solution (400 mM mannitol, 4 mM MES, pH 5.7, and 15 mM MgCl_2_) was added on ice. In particular, 20 *μ*g plasmids were added into the bottom of the tube and gently mixed. Then, an equal volume of PEG solution (400 mg/mL PEG 4000, 200 mM mannitol, and 40 mM CaCl_2_) was added and incubated at room temperature for 5 min. The protoplasts were collected and diluted with W5 solution to achieve a final concentration of 5% (*v*/*v*) serum. Next, the protoplast solution was then transferred into a 6-well plate, kept in the dark, and cultured at 22 °C overnight. Imaging was performed using an LSM700 AxioObserver (Zeiss) confocal laser scanning microscope with a Plan-Apochromat 63×/1.40 oil objective. Specifically, a 488 nm wavelength laser was utilized to excite the GFP, with the emission signal collected from 480 to 530 nm. Meanwhile, a 561 nm wavelength laser was used to excite mCherry, with the emission signal collected from 600 to 650 nm. All images were collected under a pinhole setting of 1.0 AU with a 2.0% laser intensity and digital gain of 1.0.

### Poly(A)^+^ RNA-seq

Total RNA was extracted using TRIzol Reagent (Invitrogen). The quality and integrity of the RNA were assessed by determining the RNA integrity number (RIN) through Agilent 2100 system analysis, following the manufacturer’s instructions. For each sample, 5 μg of intact total RNA with External RNA Controls Consortium (ERCC) spike-in was utilized to isolate poly(A)^+^ RNA, employing oligo(dT)_25_ Dynabeads (Thermo Fisher Scientific). The library construction was conducted using the NEBNext Ultra II Directional RNA Library Prep Kit (NEB). The sizes of RNA fragments and the generated libraries were measured using Agilent 4150 TapeStation system. The libraries were sequenced on the Illumina NovaSeq 6000 platform with a paired-end model (PE150).

### m^6^A-seq

We conducted m^6^A-seq following established protocols. Initially, 4 *μ*g of poly(A)^+^ RNA with 2 *μ*L m^6^A/A control RNA (1:1,000) was fragmented into 100 to 150 nt fragments using the magnesium RNA fragmentation module (NEB) and m^6^A-modified RNA was enriched using procedures described above. Libraries were prepared from both the input and RNA enriched with m^6^A (IP group) using the NEBNext Ultra II Directional RNA Library Prep Kit (NEB) After the size selection using DNA clean beads (Vazyme, N411), the libraries were sequenced on the Illumina NovaSeq 6000 platform with a paired-end model (PE150).

### FA-CLIP

Starting with about 3 g of formaldehyde-crosslinked 12-d-old Arabidopsis seedlings (*ECT8/ect8-1* and *ECT8m/ect8-1*), we conducted the grinding using TissueLyser II (Qiagen) at 30 Hz for 1 min and 45 s. Next, 3 mL of lysis solution (containing 150 mM KCl, 50 mM HEPES, pH 7.5, 2 mM EDTA, 0.5% [*v*/*v*] NP-40, 0.5 mM DTT, 1× cocktail protease inhibitor, and 40 U/mL RNase Inhibitor [Thermo Fisher Scientific]) was added. The mixture was incubated at 4 °C with gentle rotation. Afterward, the solution was centrifuged at 18,000 × *g* for 15 min at 4 °C, and the supernatant was filtered using a 0.22 *µ*m membrane filter. A total of 3 *μ*L of Turbo DNase (Thermo Fisher Scientific) and 3,000 U of RNase T1 (Thermo Fisher Scientific) were added, followed by a 15 min incubation at 22 °C. At the same time, 50 *µ*L Anti-Flag M2 Magnetic beads (Sigma-Aldrich) per sample were washed 4 times with 600 *µ*L low-salt wash buffer (300 mM KCl, 50 mM HEPES, pH 7.5, 0.05% [*v*/*v*] NP-40, 0.5 mM DTT, and 1× protease inhibitor [Roche]). The washed beads and the sample solution were incubated at 4 °C for 3 h. The beads were collected and washed 3 times with low-salt wash buffer (300 mM KCl, 50 mM HEPES, pH 7.5, 0.05% [*v*/*v*] NP-40, 0.5 mM DTT, and 1× protease inhibitor [Roche]). The beads were resuspended in 396 *µ*L of wash buffer with 4 *µ*L of RNase T1 (Thermo Fisher Scientific), followed by a 15 min incubation at 22 °C. After 4 washes with 500 *µ*L high-salt wash buffer (500 mM KCl, 50 mM HEPES, pH 7.5, 0.05% [*v*/*v*] NP-40, 0.5 mM DTT, and 1× protease inhibitor [Roche]), the beads were resuspended in 200 *µ*L 1× T4 PNK buffer (70 mM Tris-HCl, pH 7.6, 10 mM MgCl_2_) and 10 *µ*L T4 PNK (NEB) was added followed by a 1 h reaction at 37 °C. Afterwards, 2 *µ*L of 10 mM ATP (NEB) and additional 3 *µ*L T4 PNK (NEB) were added followed by a 30 min reaction at 37 °C. A total of 800 *µ*L 1× T4 PNK buffer was used to remove residual ATPs during washing procedure. Finally, the beads were resuspended in 180 *µ*L of 1× proteinase K reaction buffer (10 mM Tris-HCl, pH 8.0, 50 mM NaCl, 5 mM EDTA, 0.5% SDS) and 20 *µ*L of proteinase K (Thermo Fisher Scientific, 10 mg/mL) and incubated at 50 °C for 30 min. After using phenol-chloroform extraction method, 10 ng RNA extracted was used for library preparation using NEBNext Small RNA Library Prep Set for Illumina (NEB) and sequenced on the Illumina NovaSeq 6000 platform with a paired-end model (PE150).

### Nuclear run-on assay

The 12-d-old WT and *ect8-1* seedlings were ground into a fine powder and then mixed with 5 mL of cold Honda buffer. After filtering the mixture through 2 layers of Miracloth and spinning it at 2,000 × *g* for 10 min at 4 °C, the nuclei were washed for 2 or 3 times with Honda buffer. Then, all the nuclei were resuspended in 50 *µ*L storage buffer (50 mM Tris-HCl, pH 7.8, 1 mM DTT, 20% [*v*/*v*] glycerol, 5 mM MgCl_2_ and 0.44 M sucrose). Next, run-on assay was performed in a mixture that contained 10 *µ*L of a 10× transcription assay buffer, 50 *µ*L of nuclei in storage buffer, 5 *µ*L of an NTP mixture (100 mM ATP, 100 mM CTP, 100 mM GTP, and 100 mM BrUTP [Sigma-Aldrich]), and 35 *µ*L diethyl pyrocarbonate (DEPC)-treated H_2_O. This run-on reaction was carried out at 30 °C for 30 min. To stop the reaction, 900 *µ*L TRIzol Reagent (Invitrogen) was added and RNA was extracted using the Direct-zol RNA Miniprep Plus kit (ZYMO). The purified RNA was then diluted in 500 *µ*L incubation buffer (20 mM Tris-HCl, pH 7.5, 4 mM MgCl_2_, 0.2% [*v*/*v*] NP-40) and mixed with 60 *μ*L anti-BrdU beads (Santa Cruz) at 4 °C for 2 h. Finally, the precipitated RNA was extracted with TRIzol reagent and used for RT-qPCR analysis.

### mRNA lifetime sequencing

RNA stability was determined using cordycepin to inhibit transcription. In brief, we organized this experiment into 5 groups, each representing a different time point. Each group consisted 7-d-old WT and *ect8-1* seedlings (10 plants for each genotype). They were transferred to 2 mL incubation buffer (15 mM sucrose, 1 mM KCl, 1 mM PIPES, pH 6.25, and 1 mM sodium citrate). After 15 min of incubation at 80 rpm on a shaker, samples were collected at the zero time point, and the timing was started. Subsequently, the remaining samples were placed in incubation buffer containing 1 mM cordycepin (Macklin) and subjected to 3 vacuum infiltrations (0.6 Mpa, each lasting 1 min, with 1 min intervals in between). Samples were then collected at 15, 30, 60, and 120 min for further RNA extraction and subsequent experiments. Quantitative assays included the addition of the same amount of ERCC spike-in controls in same amount total RNA extracted before, which offered better correction effects for mRNA degradation-induced expression level differences compared with other endogenous genes. For library construction, poly(A)^+^ RNA of these samples was extracted using oligo(dT)_25_ Dynabeads (Thermo Fisher Scientific) followed by RNA library construction procedure. All degradation curves were fitted using the equation *y* = exp(−A × *x*), with the expression level at the initial time set as 1 to calculate the relative expression levels and half-lives of each gene at other time points.

### Ribo-seq

One gram of 12-d-old Arabidopsis seedlings was collected and rapidly frozen in liquid N_2_ to halt translation. Subsequently, the seedlings were thoroughly ground, and 1 mL of prechilled polysome extraction buffer (200 mM Tris-HCl, pH 8.0, 50 mM KCl, 25 mM MgCl_2_, 2% [*v*/*v*] polyoxyethylene (10) tridecyl ether, 1% [*w*/*v*] deoxycholic acid, 2 mM DTT, 100 *μ*g/mL cycloheximide, and 10 U/mL DNase I [Thermo Fisher Scientific]) was added. The mixture was rotated at 4 °C for 30 min and then centrifuged at 15,000 rpm at 4 °C for 30 min. 200 *μ*L samples were set aside as the Input fraction, and all remained samples were treated with MNase with 1× MNase reaction buffer for 15 min at 22 °C then quenched by adding 20 U SUPERase-In (Thermo Fisher Scientific). The MNase-digested samples were loaded onto a prechilled 10% to 50% (*w*/*v*) sucrose gradient buffer (40 mM Tris-HCl, pH 8.4, 20 mM KCl, 10 mM MgCl_2_, and 5 *μ*g/mL cycloheximide). They were centrifuged at 27,500 rpm, 4 °C for 4 h using a Beckmann SW-40Ti rotor in an ultracentrifuge, and then, the 80S monosome was separated using Bio-Rad EM-1 Econo UV monitor. RNA was extracted using TRIzol Reagent (Invitrogen), and protein was identified using SDS–PAGE. The isolated RNA was separated through a 15% (*w*/*v*) TBE-urea PAGE (Thermo Fisher Scientific), and the gel slice containing fragments of 21 to 40 nt was excised. The recovered gel was dissolved in gel recovery solution (300 mM NaAc, pH 5.5, 1 mM EDTA, and 0.1 U/mL SUPERase-In [Thermo Fisher Scientific]) and rotated at 65 °C for 10 min. The gel was filtered out using Spin-X columns (Costar), and ethanol was used to precipitate and purify RNA. Subsequently, 3′ dephosphorylation and 5′ phosphorylation were performed before library construction using NEBNext Small RNA Library Prep Set for Illumina (NEB) and sequenced on the Illumina NovaSeq 6000 platform with a paired-end model (PE150).

### m^6^A-IP-qPCR

The RNA fragmentation was performed by taking 18 *μ*L of RNA (including 1 *μ*L m^6^A/A control RNA, 1:1,000 diluted) and mixing it with 2 *μ*L of RNA fragmentation reagent. The mixture was then heated at 94 °C for 3 min. Afterward, it was immediately cooled, and 2 *μ*L of 10× RNA Fragmentation Stop Solution was added for termination. A certain volume was reserved for the input group, and the remaining samples were recovered using Oligo Clean & Concentrator kit (Zymo) following the manufacturer's instructions. A total of 25 *μ*L of protein G magnetic beads (Invitrogen) were taken and washed with 200 *μ*L of reaction buffer (150 mM NaCl, 10 mM Tris-HCl, pH 7.5, and 0.1% [*v*/*v*] NP-40). This mixture was then resuspended in 250 *μ*L of reaction buffer. Next, 1 *μ*L of m^6^A antibody (500 ng/μg, SySy) was added to it and allowed to incubate at 4 °C for 30 min. Subsequently, the beads were washed twice with reaction buffer and resuspended in 250 *μ*L of reaction buffer. An appropriate volume of RNA was added along with 2 *μ*L of RiboLock RNase inhibitor (Thermo Fisher Scientific). This mixture was then incubated at 4 °C for 1 h. The beads were washed twice with reaction buffer, low-salt buffer (50 mM NaCl, 10 mM Tris-HCl, pH 7.5, and 0.1% [*v*/*v*] NP-40), and high-salt buffer (500 mM NaCl, 10 mM Tris-HCl, pH 7.5, and 0.1% [*v*/*v*] NP-40). Finally, RNA was eluted in 50 *μ*L RLT buffer (Qiagen, #79216) at room temperature for 5 min and purified with Oligo Clean & Concentrator kit (Zymo) following procedure. All primers used are listed in Supplementary Data Set 9.

### In vivo FA-RIP

FA-RIP is based on the FA-CLIP method described previously, with the exclusion of the RNase T1 (Thermo Fisher Scientific) digestion step. In brief, seedlings crosslinked with formaldehyde were ground into a powder and incubated with lysis buffer at 4 °C for 30 min. The supernatant was collected and subjected to incubation with Anti-Flag M2 magnetic beads (Sigma-Aldrich) at 4 °C for 3 h. After washing, proteinase K (Thermo Fisher Scientific) digestion, and ethanol precipitation, the recovered RNA was reverse-transcribed to generate the first-strand cDNA. Relative enrichment levels were determined through RT-qPCR and *AT2G07689* was used as negative control. Besides that, these RNAs could also be used for UPLC-MS/MS to detect m^6^A level. All primers used are listed in Supplementary Data Set 9.

### Statistical analysis

One-way or 2-way ANOVA followed by LSD post hoc tests, Wilcoxon rank sum test (Wilcoxon test), and 2-tailed Student’s *t* test were applied for statistical analysis (Supplementary Data Set 10).

### Data analysis for poly(A)^+^ RNA-seq

The library sequencing results were initially processed by cutadapt (v4.4) for adapter trimming and then aligned to the TAIR10 reference genome using hisat2 (Kim et al. 2015) (v2.2.1). PCR duplicates were removed using SAMtools (Li et al. 2009) and Picard. Gene expression quantification was performed using featureCounts (Liao et al. 2014) (v2.0.1) and edgeR (Chen et al. 2016) (v3.36.0) to calculate counts, counts per million (CPM), and identify differential gene expression. The ERCC spike-in was used to normalize the results by using the R package RUVseq (Risso et al. 2014) (v1.28.0). GO enrichment analysis of differentially expressed genes was conducted by gene annotation with the R package org.At.tair.db (Yu et al. 2012) (v3.14.0) and gene set enrichment analysis (GSEA) pathway analysis using the R package clusterProfiler (Yu et al. 2012) (v4.2.2).

### Data analysis for m^6^A-seq

The analysis for the input group refers to poly(A)^+^ RNA-seq pipeline. Adapter trimming and size selection were performed using cutadapt (v4.4) to retain fragments with a length of 50 nt or greater. The sequences were then mapped to the reference genome (TAIR10) with hisat2 (Kim et al. 2015) (v2.2.1) using default parameters. PCR duplicates were removed using SAMtools (Li et al. 2009) and Picard. Subsequently, region enrichment analysis was conducted using macs2 callpeak (Zhang et al. 2008) (2.2.7.1) or the R package exomePeak (Meng et al. 2013) (v2.13). Regions showing enrichment with a fold change of 2 or higher and false discovery rates (FDR) < 0.05 were considered for the analysis of strand-specific m^6^A-enriched regions. We calculated the m^6^A ratio of the m^6^A-modified spike-in from the NEB EpiMark *N*^6^-Methyladenosine Enrichment Kit (*r*_spike-in_) as follows (Wei et al. 2022): (CPM_IP_ + 0.01)/(CPM_Input_ + 0.01). We defined the m^6^A normalization factor (*nf*) for each sample as the *r*_spike-in_ divided by the average *r*_spike-in_ of all WT samples. This factor represents the overall m^6^A level of each sample. Subsequently, we calculated the m^6^A level for every transcript or peak using the formula: (CPM_IP_ + 0.01)/(CPM_Input_ + 0.01) × *nf*.

### Data analysis for FA-CLIP

We initiated the analysis by employing cutadapt (v4.4) for adapter removal and filtering, retaining fragments with a length of 20 nt or longer. Subsequently, we employed hisat2 (Kim et al. 2015) (v2.2.1) to map the sequences to the reference genome (TAIR10) using default parameters. SAMtools (Li et al. 2009) and Picard were used to remove PCR duplicates. For the identification of binding sites, we used macs2 callpeak (Zhang et al. 2008) (2.2.7.1) using the --nomodel option with the criteria of enrichment ≥2 and FDR < 0.05. To evaluate the binding ability of ECT8, we calculated the enrichment fold of ECT8's binding peaks divided by the enrichment fold of ECT8m's binding peaks. Additionally, we applied a criterion based on at least a 1 nt overlap, consistent with both FA-CLIP and m^6^A-seq, to identify ECT8- and m^6^A-targeted genes using IntersectBed (Quinlan and Hall 2010).

### Data analysis for Ribo-seq

To commence the analysis, we utilized cutadapt (v4.4) for adapter trimming and filtered the fragments, retaining those with a length of 20 nt or longer. Then, we applied hisat2 (Kim et al. 2015) (v2.2.1) with default parameters to map these sequences to the reference genome (TAIR10). To quantify transcript counts, we used featureCounts (Liao et al. 2014) (v2.0.1), focusing specifically on CDS annotated in Araport11 (Cheng et al. 2017). For calculations of CPM, we employed the R package edgeR (Chen et al. 2016) (v3.36.0). The formula for calculating TE is as follows: TE = CPM_Ribo_/CPM_Input_.

### Accession numbers

Sequence information of the genes studied in this article can be found in the Arabidopsis TAIR database (https://www.arabidopsis.org) under the following accession numbers: *ECT1* (AT3G03950), *ECT2* (AT3G13460), *ECT3* (AT5G61020), *ECT4* (AT1G55500), *ECT5* (AT3G13060), *ECT6* (AT3G17330), *ECT7* (AT1G48110), *ECT8* (AT1G79270), *ECT9* (AT1G27960), *ECT10* (AT5G58190), *ECT11* (AT1G09810), *ECT12* (AT4G11970), *CPSF30-L* (AT1G30460), *MTA* (AT4G10760), *MTB* (AT4G09980), *FIP37* (AT3G54170), *VIR* (AT3G05680), *HAKAI* (AT5G01160), *HIZ1* (AT1G32360), *HIZ2* (AT5G53440), *ALKBH9B* (AT2G17970), *ALKBH10B* (AT4G02940), *DCP1* (AT1G08370), *DCP2* (AT5G13570), *DCP5* (AT1G26110), *NOT1* (AT1G02080), *AGO1* (AT1G48410), *VCS* (AT3G13300), *XRN4* (AT1G54490), *PPRT1* (AT1G68820), *MSI1* (AT5G58230), *BIL1* (AT2G30980), and *ENGD-1* (AT1G30580). The raw sequencing data of poly(A)^+^ RNA-seq, FA-CLIP, m^6^A-seq, Ribo-seq, and mRNA lifetime sequencing reported in this paper have been deposited in the BIG Data Center (http://bigd.big.ac.cn) under the project number PRJCA020765.

## Supplementary Material

koae149_Supplementary_Data

## Supplementary data

The following materials are available in the online version of this article.

**Supplementary Figure S1.** ECT8 is an m^6^A-binding protein, sharing sequence similarity to other YTH family proteins.

**Supplementary Figure S2.** Material characterization for *ect8-1* T-DNA mutant and transgenic plants.

**Supplementary Figure S3.** The expression pattern and subcellular localization of ECT8.

**Supplementary Figure S4.** ECT8 is highly expressed and quickly responds to salt stress in an m^6^A-dependent manner.

**Supplementary Figure S5.** ECT8 consistently binds to the 3′-UTR of mRNA under normal and salt stress conditions.

**Supplementary Figure S6.** Transcriptomic analysis indicates that ECT8 enhances m^6^A-modified mRNA decay.

**Supplementary Figure S7.** ECT8 does not globally influence TE.

**Supplementary Figure S8.** ECT8 collaborates with DCP5 and VCS rather than interacting with other P-body components.

**Supplementary Figure S9.** GO analysis of upregulated ECT8- and m^6^A-targeted genes in *ect8-1* under different conditions.

**Supplementary Figure S10.** ECT8 does not influence the transcription of negative regulators of the salt stress response.

**Supplementary Data Set 1.** Statistical analysis of m^6^A-seq under mock condition.

**Supplementary Data Set 2.** Statistical analysis of m^6^A-seq under salt condition.

**Supplementary Data Set 3.** Statistical analysis of ECT8-binding sites under mock condition.

**Supplementary Data Set 4.** Statistical analysis of ECT8-binding sites under salt condition.

**Supplementary Data Set 5.** Statistical analysis of RNA-seq between WT and *ect8-1* under mock condition.

**Supplementary Data Set 6.** Statistical analysis of RNA-seq between WT and *ect8-1* under salt condition.

**Supplementary Data Set 7.** Statistical analysis of RNA lifetime between WT and *ect8-1*.

**Supplementary Data Set 8.** Statistical analysis of Ribo-seq between WT and *ect8-1*.

**Supplementary Data Set 9.** List of primers and oligonucleotides used in this study.

**Supplementary Data Set 10.** Statistical analysis in this study.

## Dive Curated Terms

The following phenotypic, genotypic, and functional terms are of significance to the work described in this paper:

PAB4 Gramene: AT2G23350
PAB4 Araport: AT2G23350

SDS CHEBI: CHEBI:8984

AT1G79440 Gramene: AT1G79440
AT1G79440 Araport: AT1G79440

AT2G07689 Gramene: AT2G07689
AT2G07689 Araport: AT2G07689

HEPES CHEBI: CHEBI:46756

PAB2 Gramene: At4G34110
PAB2 Araport: At4G34110

ABA CHEBI: CHEBI:2365

GSEA Gramene: Gene set enrichment analysis
GSEA Araport: Gene set enrichment analysis
