# Supplementary material for: The m6A reader ECT8 is an abiotic stress sensor that accelerates mRNA decay in Arabidopsis
Source: Plant Cell. 2024 Jun 5;36(8):2908–26. doi: 10.1093/plcell/koae149 (PMC11289641; doi:10.1093/plcell/koae149)
Supplement: koae149_Supplementary_Data [file koae149_supplementary_data.zip › TPC2023RA01169R2_Supplementary figures.pdf]

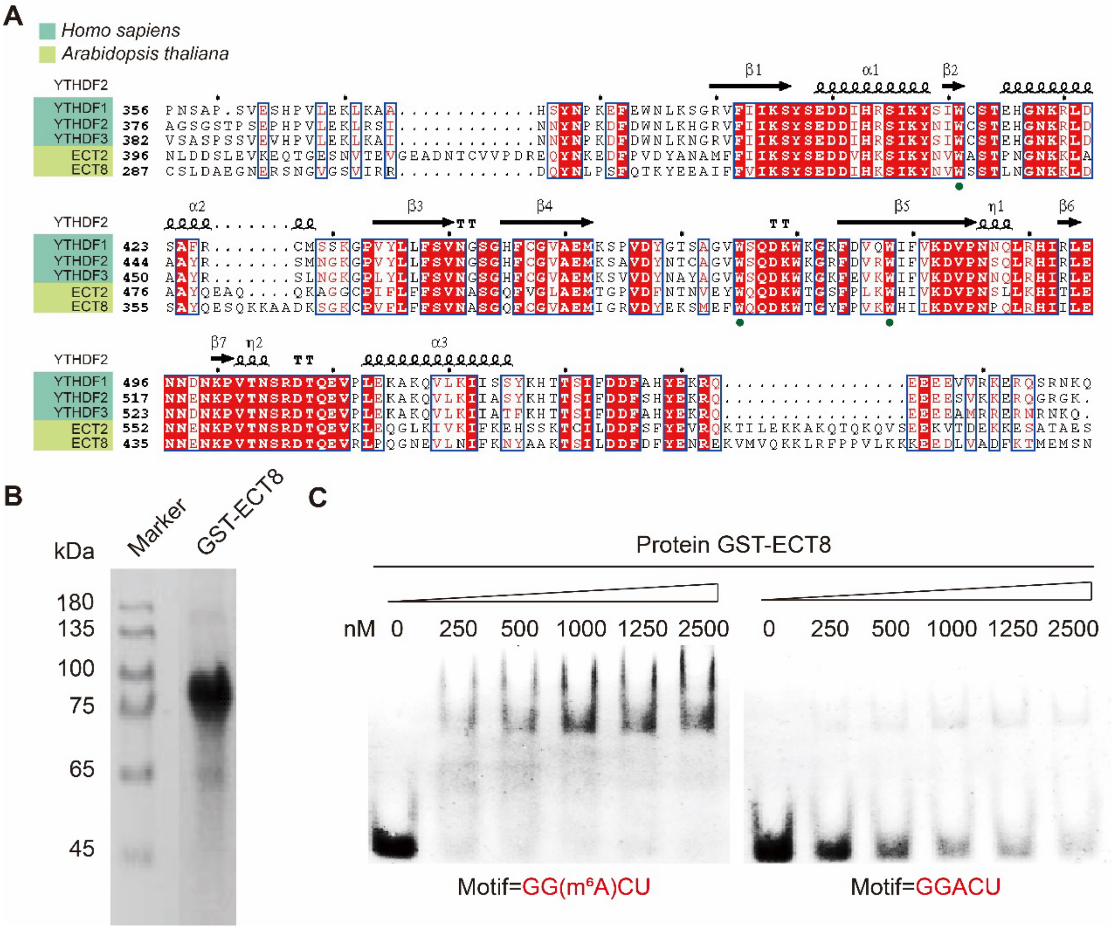

Supplementary Figure S1. ECT8 is an m^{6}A-binding protein, sharing sequence similarity to other YTH family proteins.

(Supports Figure 1)

(A) Protein sequence alignment of ECT8 with YTHDF1-3 in mammals and ECT2 in Arabidopsis (https://espript.ibcp.fr/ESPript/ESPript/index.php) and only the YTH domains are depicted in this alignment. Three important tryptophan residues for m^{6}A binding are highlighted and dotted with green. 3_{10}-helices, α-helices and π-helices are displayed as small, medium and large squiggles, respectively. β-strands are rendered as arrows, strict β-turns as **TT** letters. Regions filled in red mean strict identity, red characters represent similar amino acids, blue frames represent high similarity across groups, black letters indicate amino acids with low similarity, and dotted line are used to represent gaps. The similarity was calculated using PAM250 matrix from the website sited above.

(B) SDS-PAGE result for protein purification of GST-ECT8. kDa, kilodalton.

(C) EMSA confirms the binding affinity of GST-ECT8 with RNA probe containing m^{6}A-modified GGACU motif but not unmethylated probe. Each lane was loaded with varying concentrations (shown below the triangle panel) of protein and a consistent amount of RNA oligo with a final concentration of 4 nM.

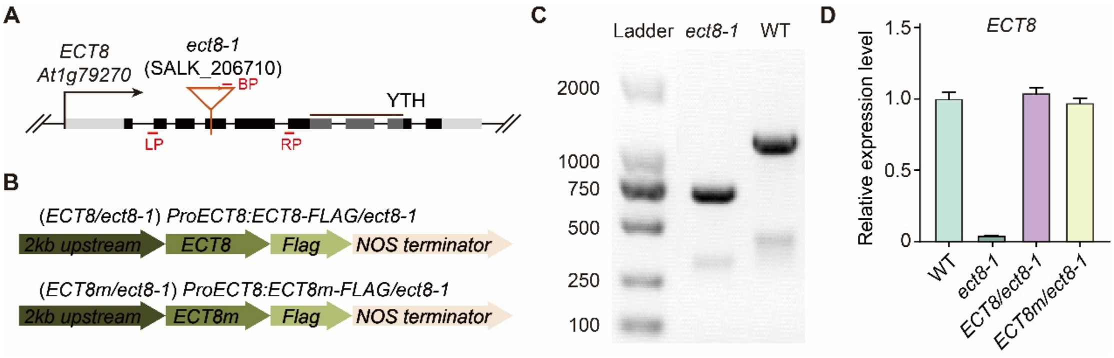

Supplementary Figure S2. Materials characterization for *ect8-1* T-DNA mutant and transgenic plants.
(Supports Figure 1)

(A) Schematic representation of *ECT8* loci and the *ect8-1* T-DNA insertion line. Exons are depicted as boxes and introns as lines, as well as YTH domain is highlighted. The location of primers that used for genotyping are marked in red lines. LP, left genomic primer; RP, right genomic primer; BP, T-DNA border primer.

(B) Schematic representation of transgenic structure of *ECT8/ect8-1* as well as *ECT8m/ect8-1*. *NOS* (nopaline synthase) terminator is used to ensure correct transcription termination during gene expression.

(C) PCR-based genotyping to confirm the homozygous *ect8-1* mutant. WT, wild-type.

(D) RT-qPCR results for the relative expression level of *ECT8* or *ECT8m* in WT, *ect8-1*, *ECT8/ect8-1* and *ECT8m/ect8-1*. WT, wild-type. *TUB8* is used as a reference control. Data are presented as means ± SE, n = 3 independent experiments, each with 3 technical replicates.

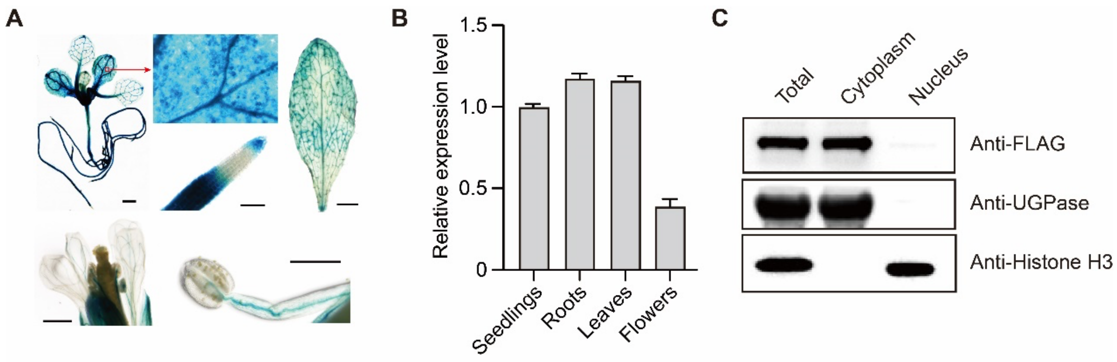

Supplementary Figure S3. The expression pattern and subcellular localization of ECT8.
(Supports Figure 2)

(A) GUS staining assay illustrating the expression pattern of ECT8 in different tissues. Scale bar = 1 mm.

(B) Expression pattern of *ECT8* analyzed from the Arabidopsis RNA-seq database. Data are presented as means ± SE, n = 58 independent sequencing data from database.

(C) Protein immunoblot showing that ECT8 protein primarily localizes in cytoplasm rather than nucleus. UGPase and H3 are used as cytoplasmic and nuclear protein markers, respectively.

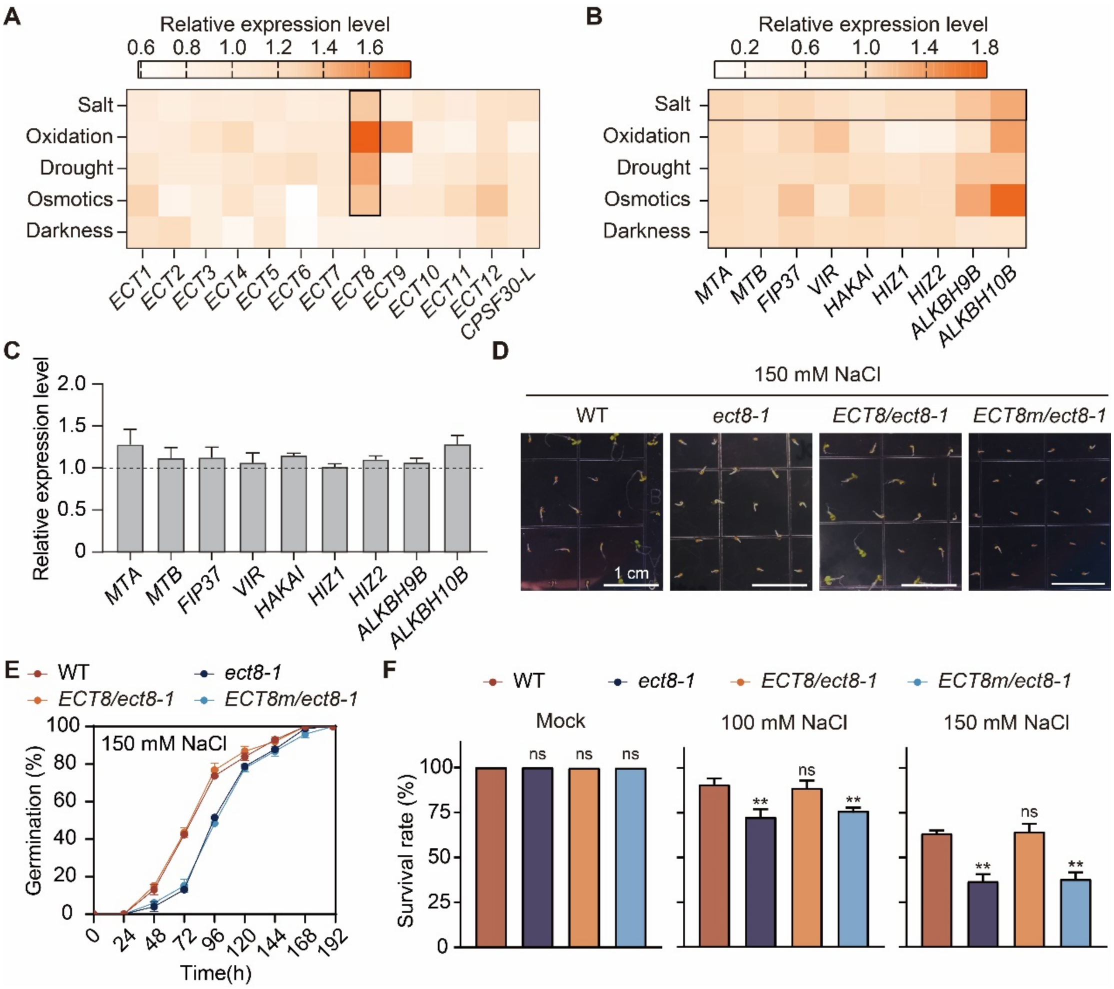

Supplementary Figure S4. ECT8 is highly expressed and quickly responds to salt stress in an m^{6}A-dependent manner.

(Supports Figure 2)

(A) The higher sensitivity of ECT8 protein to various external stresses among YTH family proteins. The data analyzed from the Arabidopsis RNA-seq database (https://plantrnadb.com/athrdb/).

(B) Heatmap displays a negligible change in the relative expression levels of m^{6}A writers or erasers. The data analyzed from the Arabidopsis RNA-seq database (https://plantrnadb.com/athrdb/).

(C) RT-qPCR indicating that there is only a slight increase in the relative expression levels of m^{6}A writers or erasers under 4-hour treatment with 150 mM NaCl comparing to mock conditions. *TUB8* was used as internal control. Data are presented as means ± SE, n = 3 independent experiments, each with 3 technical replicates.

(D) Phenotypic analysis of salt response in WT, *ect8-1*, *ECT8/ect8-1*, and *ECT8m/ect8-1* plants under 150 mM NaCl treatments. Representative images showing the morphology of 6-d-old seedlings. WT, wild-type.

(E) Statistical analysis of germination rates in WT, *ect8-1*, *ECT8/ect8-1*, and *ECT8m/ect8-1* plants under mock control and 150 mM NaCl treatment. WT, wild-type. Data are presented as means ± SE, n = 4 independent experiments, each with at least 35 seedlings.

(F) Statistical analysis of survival rate in WT, *ect8-1*, *ECT8/ect8-1*, and *ECT8m/ect8-1* plants under mock control, 100 mM and 150 mM NaCl treatment, respectively. WT, wild-type. Data are presented as means ± SE, n = 4 independent experiments, each with at least 35 seedlings. ns, not significant and ***P* < 0.01 by one-way ANOVA.

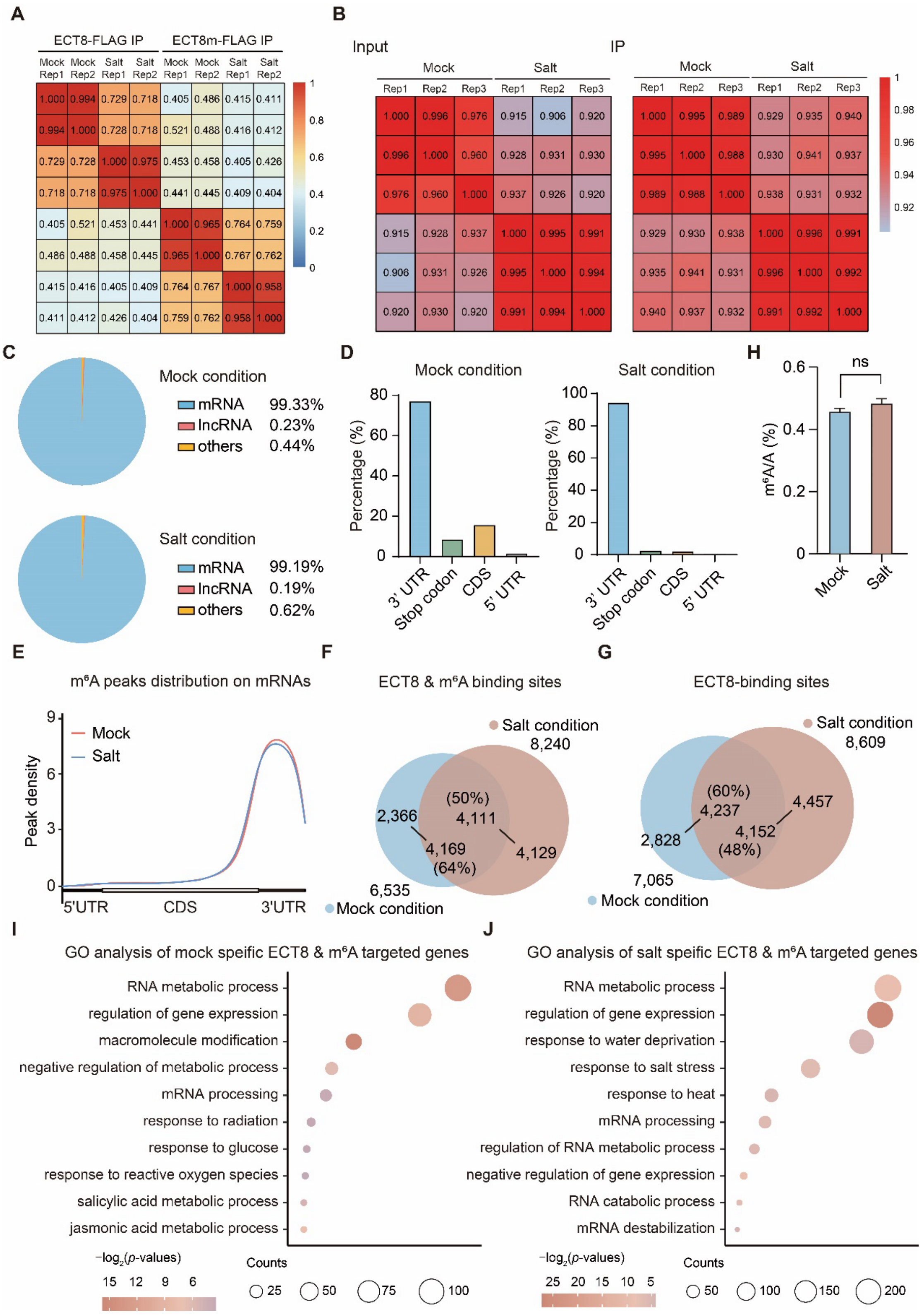

Supplementary Figure S5. ECT8 consistently binds to the 3'-UTR of mRNA under normal and salt stress conditions.

(Supports Figure 3)

(A) Heatmap showing the correlation of FA-CLIP data between *ECT8/ect8-1* and *ECT8m/ect8-1* under mock and salt stress conditions. IP, immunoprecipitation.

(B) Heatmap showing the correlation of m^6A-seq data of Input (left) and IP (right) group under mock and salt stress conditions. IP, immunoprecipitation.
(C) Pie chart depicting the transcript distribution of ECT8 & m^6A targeted genes under mock (up) and salt (down) conditions.
(D) Histogram plot showing the region distribution of ECT8 & m^6A targeted peaks under normal (left) and salt stress (right) conditions. 5′ UTR, 5′ untranslated region; CDS, coding sequence; 3′ UTR, 3′ untranslated region. The stop codon is defined as the region from −10 bp upstream to +10 bp downstream of the stop codon.
(E) Metaplot showing the distribution of m^6A peaks on mRNA under mock and salt conditions. 5′ UTR, 5′ untranslated region; CDS, coding sequence; 3′ UTR, 3′ untranslated region.
(F) Venn plot showing the overlap ratio of ECT8 & m^6A binding sites under mock and salt conditions.
(G) Venn plot showing the overlap ratio of ECT8-binding sites under mock and salt conditions.
(H) Mass spectrometry showing the m^6A level of poly(A)$^+$ RNA extracted from 12-d-old WT seedlings under mock and 4-hour 150 mM NaCl treatment. Data are presented as means ± SE, n = 3 independent experiments, each with 3 technical replicates. ns, not significant by two-tailed student *t*-test (paired).
(I) GO analysis of 1,381 mock specific ECT8 & m^6A targeted genes. *p*-values were calculated from DAVID website (https://david.ncifcrf.gov/).
(J) GO analysis of 3,172 salt specific ECT8 & m^6A targeted genes. *p*-values were calculated from DAVID website (https://david.ncifcrf.gov/).

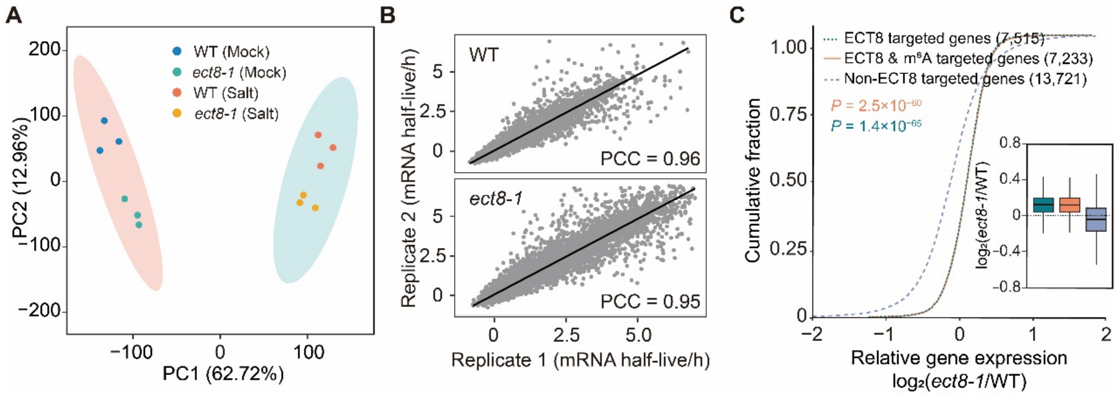

Supplementary Figure S6. Transcriptomic analysis indicates that ECT8 enhances m^{6}A-modified mRNAs decay.

(Supports Figure 4)

(A) Principal component analysis (PCA) analysis showing the correlation of RNA-seq data between WT and *ect8-1* mutant under mock and salt stress conditions. WT, wild-type.

(B) The replicates of mRNA life-time sequencing show high repeatability. PCC: Pearson Correlation Coefficient. WT, wild-type.

(C) Cumulative distribution of relative mRNA expression of 7,515 ECT8 targeted genes, 7,233 ECT8 & m^{6}A targeted genes and 13,721 non-ECT8 targeted genes in *ect8-1* compared to WT under salt stress condition. WT, wild-type. Lower and upper hinges represent first and third quartiles, the center line represents the median, and whiskers represent ±1.5× interquartile range. *p*-values were calculated using Wilcoxon test.

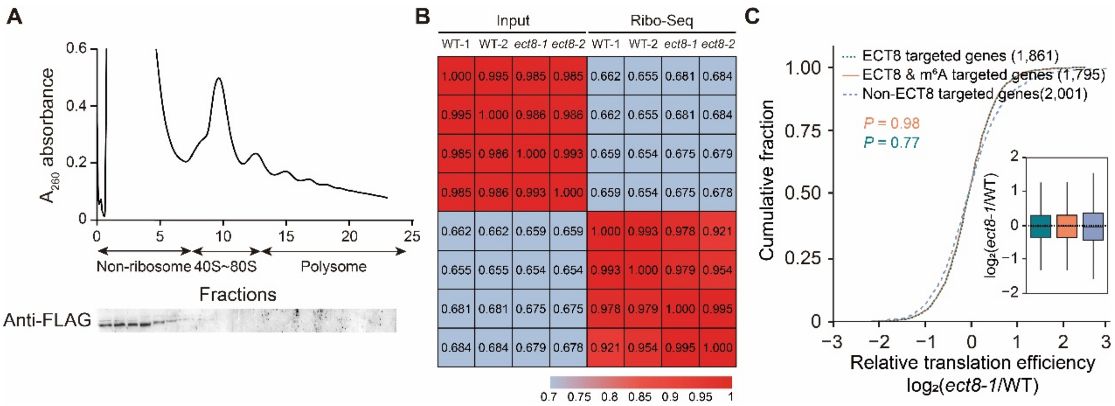

Supplementary Figure S7. ECT8 does not globally influence translation efficiency.

(Supports Figure 4)

(A) Polysome profiling experiment combined with protein immunoblot showing ECT8 only locates in non-ribosome fractions.

(B) Heatmap showing the correlation of ribosome sequencing (Ribo-seq) data, including Input group. WT, wild-type.

(C) Cumulative distribution of relative translation efficiency of 1,861 ECT8 targeted genes, 1,795 ECT8 & m^6A targeted genes and 2,001 non-ECT8 targeted genes in *ect8-1* compared to WT. WT, wild-type. Lower and upper hinges represent first and third quartiles, the center line represents the median, and whiskers represent ±1.5× interquartile range. *p*-values were calculated using Wilcoxon test.

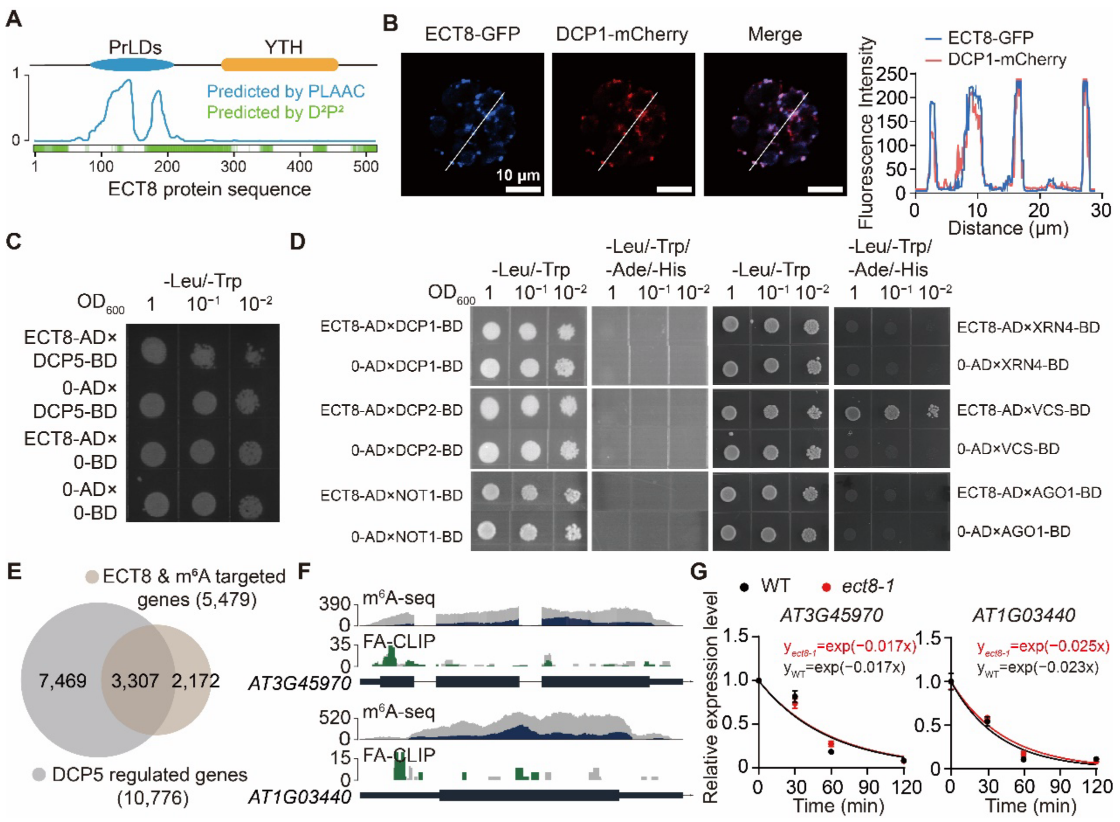

Supplementary Figure S8. ECT8 collaborates with DCP5 and VCS rather than interacting with other P-body components.

(Supports Figure 5)

(A) Top, Protein domain structure of ECT8. Bottom, Predictions of PrLDs and disordered regions by ‘Prion-like Amino Acid Composition’ (PLAAC; http://plaac.wi.mit.edu/) and D^2P^2 algorithm. PrLDs, Prion-like domain.

(B) Confocal microscopy showing the co-localization of ECT8-GFP and DCP1-mCherry in P-bodies from protoplast co-expression experiment. Intensity traces (white lines) are analyzed by ImageJ and plotted at right. Scale bar = 10 μm.

(C) The positive control of interaction between ECT8 and DCP5. -Leu-Trp: the selective medium without tryptophan and leucine. AD, the activation domain expressed from *pGADT7*; BD, the binding domain expressed from *pGBKT7*. 0-AD, the empty vector of *pGADT7*; 0-BD, the empty vector of *pGBKT7.*

(D) Y2H assay showing that there are no physical associations between ECT8 and DCP1, DCP2, NOT1, XRN4 or AGO1 in yeast cells instead of VCS. The full-length coding sequences of these genes were fused with the GAL4-AD or BD domain as indicated. -Leu-Trp, selective medium without tryptophan and leucine. -Leu-Trp-Ade-His: the selective medium without tryptophan, leucine, histidine, and adenine. AD, the activation domain expressed from *pGADT7*; BD, the binding domain expressed from *pGBKT7*. 0-AD, the empty vector of *pGADT7*.

(E) Venn plot indicating the overlap ratio between ECT8 & m^6A targeted genes and DCP5 regulated genes.

(F) Integrative genomics viewer (IGV) showing the m^6A-seq and FA-CLIP sequencing results on *AT3G45970* and *AT1G03440* transcripts. FA-CLIP, formaldehyde

crosslinking and immunoprecipitation.

(G) The RNA half-lives of *AT3G45970* and *AT1G03440* transcripts in 7-d-old WT and *ect8-1* seedlings. External spike-ins were used as internal control. Data are presented as means ± SE, n = 2 independent experiments, each with 3 technical replicates.

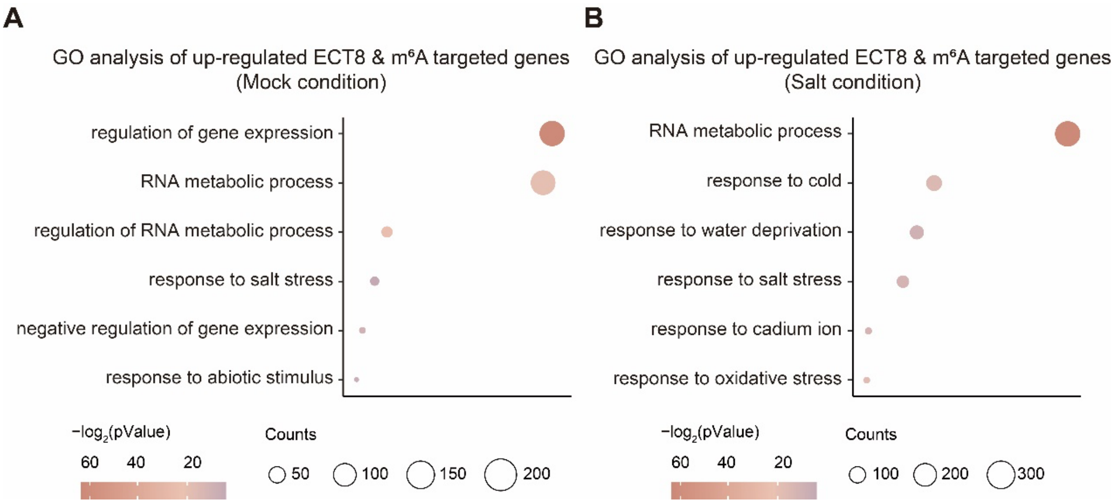

Supplementary Figure S9. GO analysis of up-regulated ECT8-and m^{6}A-targeted genes in *ect8-1* under different conditions.

(Supports Figure 6)

(A, B) GO analysis of ECT8 & m^{6}A targeted genes which are up-regulated (termed as up-regulated ECT8 & m^{6}A targeted genes) in *ect8-1* under mock condition (3,279) (A) and (4,663) salt condition (B) revealed a consistent enrichment in biological processes including RNA metabolic process and response to salt stress. *p*-values were calculated from DAVID website (https://david.ncifcrf.gov/).

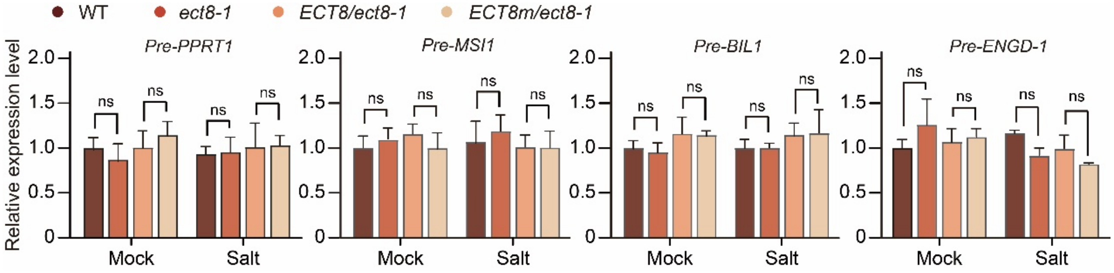

Supplementary Figure S10. ECT8 does not influence the transcription of salt stress negative regulators of the salt stress response.
(Supports Figure 6)
RT-qPCR analysis reveals that the pre-mRNA expression level of these downstream candidates remains largely unchanged under both mock and salt stress treatments. WT, wild-type. Data are presented as means ± SE, n = 3 independent experiments, each with 3 technical replicates. ns, not significant by two-way ANOVA.
