# Supplementary material for: The m6A reader ECT8 is an abiotic stress sensor that accelerates mRNA decay in Arabidopsis
Source: Plant Cell. 2024 Jun 5;36(8):2908–26. doi: 10.1093/plcell/koae149 (PMC11289641; doi:10.1093/plcell/koae149)
Supplement: koae149_Supplementary_Data [file tpc.01169.2023-s05.pdf › tpc.01169.2023-s05.pdf]

THE PLANT CELL

m6A reader ECT8 functions as an abiotic stress sensor by accelerating mRNA decay in Arabidopsis

Zhihe Cai, Qian Tang, Peizhe Song, Enlin Tian, Junbo Yang, and Guifang Jia

Review Timeline:

Submission Date:	05-Dec-2023
Editorial Decision:	22-Jan-2024
Revision Received:	04-Apr-2024
Editorial Decision:	10-Apr-2024
Revision Received:	11-Apr-2024
Accepted:	11-Apr-2024

1st Editorial Decision 22-Jan-2024

Prof. Dr. Guifang Jia
Peking University
Beijing
China

Dear Dr. Jia:

We have received reviews of your manuscript entitled "m6A reader ECT8 functions as an abiotic stress sensor by accelerating mRNA decay in Arabidopsis." Thank you for submitting your best work to The Plant Cell. The editorial board agrees that the work you describe is substantive, falls within the scope of the journal, and may become acceptable for publication, pending revision and potential re-review.

We ask you to pay attention to the following points in preparing your revision. Specifically, please address all reviewer comments as thoroughly as possible, most or which can be addressed through editorial changes rather than significant new experimentation.

Please contact us if there are ambiguous comments or if you wish to discuss the revision.

Given the nature of the comments, we are offering you 60 days from when we have issued this decision to complete the revision. If a revision is not returned within this time frame, and if you have not been granted an extension, we will withdraw the manuscript, which will leave you free to submit the work elsewhere. If you need an extension, we encourage you to contact us at any point before the 60 days have passed.

When you are ready to submit the revised version, please upload a highlighted copy that indicates all changes made in response to the editor and reviewer recommendations. Include an itemized list of all changes made in response to each of the reviewer's suggestions in the "Response to Reviewers" section; please note that reviewers do not have access to your cover letter, nor was this decision letter shared with them.

Thank you for the privilege of reviewing your work. We look forward to receiving your revised manuscript.

Sincerely,

The Plant Cell Editorial Board

Please note the following:

-The Plant Cell now requires authors to complete and submit an author revisions checklist upon submission of a revised manuscript. The aim of the checklist is to aid authors in preparing a high-quality manuscript, facilitate the review and assessment of revised manuscripts, and help to ensure that journal standards are maintained across the board. If your manuscript is accepted, the completed checklist will be published as supplemental material attached to the article online. Please download a copy of the checklist (pdf fillable form) at this link, for submission with your revised manuscript: https://tpc.msubmit.net/html/Author_Revisions_Checklist.pdf.

-Supplemental materials should be restricted to large datasets and tables, presentation of replicates, and validation of reagents, methods, or genotypes. Any data that are used to support the major claims must be in the main manuscript. Supplemental figure legends must indicate what figure in the main manuscript is supported by the supplemental data presented. Please justify how each of the supplemental figures meet the criteria.

-Sampling methods and nature of "biological replicates" should be described precisely (i.e. different plants, parts of plants, pooled tissue, independent pools of tissue, sampled at different times, etc), along with a clear description of and rationale for any statistical analyses conducted. The reader should know exactly what was sampled; what forms the basis of the calculation of any means and statistical parameters reported. This is also necessary to ensure that proper statistical analysis was conducted.

-Want to add this revision deadline to your calendar? Click below!

-- Reviewer comments:

Reviewer #1 (Comments for the Author):

The manuscript "m6A reader ECT8 functions as an abiotic stress sensor by accelerating mRNA decay in Arabidopsis" by Cai et al. describes the role of m6A reader ECT8 under salt stress in Arabidopsis. The authors show that ECT8 binds to m6A modified RNA and the target RNAs have variable lifetime in the ect8-1 mutant. Overall the results and the methods implemented to get said results are satisfactory. m6A and several m6A related proteins have now been shown to be involved in stress regulation. Just recently ECT1 has been shown to be involved in Arabidopsis stress response (Lee et al 2023). However, in the current manuscript authors do show the binding of ECT8 to RNA and provide important datasets like FA-CLIP which helps with the novelty of research.
Below you will find my comments, questions and suggestions (in order of appearance in the MS):
1. The figure numbers in line 138 and 139 are not correct. There is no figure S1D
2. In the section "ECT8 is highly expressed in flowers, roots, and leaves and localized in cytoplasm" I would like to ask why the authors did not use a fluorescent tagged ECT8 line (they use ECT8:GFP and YFP constructs in later sections) to show the localization of ECT8. I do not have anything against the GUS reporter assay, however, as we know, ECT8 has a N-terminal disordered region and according to authors is localized in P-bodies, it would be far more interesting if the authors could show the localization of ECT8 in planta under control and stress conditions.
Minor: line 147 should be ECT8prom:GUS instead of ECT8:GUS
3. In the section "Salt stress induces a significant increase in transcription and expression of ECT8" authors monitor the level of ECT8 mRNA and pre-mRNA under stress. I have a few issues in this section:
a. How did the authors make the distinction between mRNA and pre-mRNA?
b. The primers listed in the supplementary data table are same for both mRNA and pre-mRNA. As far as I know, it is impossible to distinguish these based solely on primers.
c. The methods section for RT-qPCR states "a minimum of two biological replicates". This is not clear. I suggest authors have 3 replicates at the least in every experiment.
d. In the western blots showing ECT8 protein levels, what might be the reason behind two bands? Which band is ECT8? According to Uniprot ECT8 has a MW of 59.5kDa, however the bands shown are more likely around 70kDa.
e. Authors should quantify the bands using ImageJ or similar software since (at least to my eyes) it seems that the band intensity of Mock at 0H is similar to that of treated at 5H. I would assume that at 0H both should have similar intensity bands.
4. In the section "ECT8's m6A binding capability is required for responding to salt stress" In the methods related to this section, please specify how many root lengths were measured.
5. In the section "ECT8 binds to 3' UTR of mRNA with m6A modification under normal and salt stress conditions"
a. As mentioned earlier, only 2 biological replicates were used for FA-CLIP and m6A-seq. Since these are novel data they will be strengthened by the presence of 3 replicates. Authors should add the 3rd replicate and show proof of replicability.
b. The language in this section and the following sections regarding the transcripts that are bound by ECT8, bound by ECT8 and have m6A and not bound by ECT8 needs to be clarified. At present it is not clear when authors talk about peaks, transcripts or genes. I suggest consistency in text and figures about whether they depict transcripts or genes associated to those transcripts.
c. In order to verify the quality of m6A-seq a metaplot to show the m6A peak distribution should be added to supplementary dataset.
6. In section "Elevated ECT8 protein enhances its binding capacity to m6210 A-modified mRNAs under salt stress"
a. Line 217-218: "......stress did not disturb ECT8's binding positions (Figure E)" Figure E what?
b. Regarding figure 3G and 3H, it is unclear to me what 'Peak enrichment fold' means? How is this metric calculated and how does it corelate to ECT8's binding efficiency? Please clarify in methods.
c. In figure 3H, it seems that m6A levels go down after salt stress. However, in the text authors write "significantly increased binding affinity to ECT8 & m6A common targets, even in the absence of a substantial increase in overall m6A abundance" line 225-226. If the m6A levels go down after salt stress, what is ECT8 binding to? This is also where the metaplots for m6A-seq could come in handy.
7. In section "ECT8 promotes m6276 A-modified mRNA decay through direct interaction with DCP5 in P-bodies"
a. Authors use DCP1 to establish that ECT8 localizes in P-bodies. However, every experiment after that uses DCP5. Why?
b. Multiple proteins have been shown to be in P-Bodies, XRN4, VCS, AGO1, etc. The authors did not show if ECT8 interacts exclusively with DCP5 or whether it also interacts with these other proteins? A wider Y2H screen could identify more interactors providing more insight.
c. Since AGO1 is also a part of P-bodies, could miRNA mediated mRNA decay also play a part in this? It is known that m6A also affects miRNA biogenesis (Bhat et al., 2020), how does that affect authors hypothesis.
8. In section "ECT8 destabilizes negative regulators of salt stress response for enhancing salt stress tolerance" line 352-353: "The results showed that they underwent more rapid degradation in ect8-1 compared to WT under both normal and salt stress conditions" I think the authors have misstated the results. Since the figures show stabilization in ect8 mutant.

Reviewer #2 (Comments for the Author):

This article focuses on N6-methyladenosine (m6A), the most prevalent mRNA modification in eukaryotes, including plants, and its roles in various biological processes such as plant growth, development, and stress response. It highlights the identification of ECT8 as an m6A reader protein crucial for the plant's response to salt stress. ECT8 enhances the degradation of target transcripts by interacting with the decapping protein DCP5 within P-bodies, a process particularly important under various environmental stresses. The study observes a significant increase in ECT8 expression under such conditions, and specifically in response to salt stress, both at the transcript and protein levels. This increase in ECT8 leads to more effective binding to m6A-modified mRNAs, promoting their degradation, especially those negatively regulating salt stress response. The findings demonstrate ECT8's role as a sensor of abiotic stresses, aiding in mRNA decay to maintain homeostasis and improve stress tolerance in plants. These insights contribute to the understanding of epitranscriptomic gene regulation and have potential applications in breeding more resilient crops amid changing environmental conditions.
Believing that the article will be read and will be cited, I have a few remarks for the authors to implement corrections or possibly respond to them.

1) I believe that there is a particular lack in Fig S3 of results that show the preferential localization of ECT8 in the cytoplasm using some bioimaging techniques. Ideally, this should be shown for different plant tissues for comparison.
2) Please correct the reference to Fig in line 218 of the text (it is Figure E); it should be 3E.
3) The image in Figure 5A should include the localization of the cell nucleus. This is a note in addition to point 1. Regarding this image, a better marker for P bodies would be DCP2 or of course DCP5, if the authors have the possibility to show these colocalizations with ECT8. However, if it is not feasible to make this correction to the manuscript within the necessary timeframe, the localization of DCP1 is acceptable.
4) In the diagram (Figure 7) summarizing the study results, the site where mRNA degradation occurs through the interaction of ECT8 with DCP5 is indicated as P bodies. This is likely, but since the authors have not presented direct evidence of such localization of this process in P bodies, at this stage of the research it is only a hypothesis, and this should at least be mentioned in the figure description.
5) It's not required, but I really feel the absence of an experiment to find protein partners of ECT8. I am referring to the proteomic analysis of immunoprecipitates (IP-LC-MS/MS). This would provide additional confirmation for DCP5 beyond the Yeast two-hybrid (Y2H) assay, as well as addressing what the authors discuss in lines 420-423.

Reviewer #3 (Comments for the Author):

The manuscript by Cai, Tang and Song et al. reports the function of the m6A reader ECT8 in Arabidopsis. The authors demonstrate ECT8 plays a role in salt stress tolerance and contributes to mRNA decay through its interaction with the decapping activator DCP5. Overall, I find the work interesting and novel, with genetic evidence and a series of genome-wide omics data to support the authors' conclusion. The study is expansive and provides new findings related to the regulation of mRNA fates by an m6A reader.
Please see below my specific comments and questions intended to improve the quality of the manuscript.

Major comments:

1. Western blot images of FLAG-tagged ECT8
The western blot images of FLAG-tagged ECT8 (Figure 2B) showed two discreet bands with anti-FLAG detection. What would be an explanation of for this phenomenon? This is consistent with the data in Supplemental Figure S3C. it seems to me that the total protein fraction showed ECT8 detection with two sizes, while the cytosolic fraction contained the band of smaller size and the nuclear fraction with the larger size. I am not convinced that ECT8 is preferentially cytosolic. The anti-FLAG panel of Supplemental Figure S3C should be cropped in a way that the upper band is not omitted.

2. Comparison of ECT8-binding sites and m6A peaks
The authors compared m6A-seq with FA-CLIP data under both mock (control) and salt stress conditions. This showed that the majority of ECT8 targets have m6A marks, which makes sense. However, I find the number labeling format for Figures 3A and 3B confusing. I don't understand why there were two numbers for the overlaping region of the Venn diagrams. In Figure 3A, for example, it would be understandable if there were 16,177 m6A targets and 7,592 ECT8 targets detected, with the overlap of 5,656. But the authors also labelled 5,684 m6A targets and 6,841 ECT8 targets in the overlapping region that do not add up to 5,656. Please clarify why this was the case. More explanation in the text and/or figure caption would be helpful.

I am also confused why the number of ECT8 & m6A binding sites presented in Figure 3F did not correspond with the number in Figures 3A and 3B. My understanding is that there were 5,656 ECT8 & m6A targets under mock condition and 7,075 under salt stress conditions. However, the number in Figure 3F was 6,841 and 8,119, respectively. Was this because Figures 3A and 3B referred to the number of genes but 3F referred to the number of sites? Whatever the case, the analysis and comparison should be described more clearly in the text and figure captions. The distinction between targets and sites should be clearly defined and presented systematically. Perhaps replacing Figure 3F with Figure S5D would make it easier for the reader to follow.

By comparing Figure 3A with Figure 3B, there was increase of the ECT8 & m6A targets from 5,656 targets under mock condition to 7,075 under salt condition. Is there an enrichment for specific biological functions from those 1,419 targets that was increased by salt stress?

The color scheme for Figure 3F should be changed from blue-green to blue-red as in Figure 2C, as this was a comparison of the mock and salt stress conditions. I understand that GO enrichment analysis was performed on ECT8 & m6A common (mock VS control) targets and presented in Figure 3I, but what about those that are unique to either mock or salt stress conditions?

Lines 224-228: The author wrote, "It has been observed that under salt stress, ECT8 exhibits a significantly increased binding affinity to ECT8 & m6A common targets, even in the absence of a substantial increase in overall m6A abundance or the expression levels of m6A writer subunits and erasers (Figure 3, G and H; Supplemental Figure S4, B and C; Supplemental Figure S5E)." In fact, Figure 3H indicates that m6A level was significantly decreased for ECT8 & m6A common targets under salt stress conditions. The text should be corrected accordingly.

3. mRNA lifetime sequencing & ribosome-sequencing
There was no indication on how many replicate samples were used for mRNA half-life sequencing, and what the statistical model/criterion was used to compare the mRNA half-life between the genotypes. This makes it impossible to determined the quality of the mRNA decay data and whether the authors' interpretation of the data was valid. The data in Figure 4B were presented as cumulative fraction of relative mRNA half-live between ect8-1 mutant and the wild type, without any other information provided to allow for an assessment of the efficiency of cordycepin treatment. What is the number of seedlings used in the cordycepin treatment experiment? Also the libraries was made with poly(A) selection, which overlooked mRNA decay intermediates without poly(A) tails.

This was also the case for the quality of ribosome-sea data. The authors made a conclusion that ECT8 mutation has no effects on translation efficiency of ECT8 & m6A targets, but without providing sufficient evident that the conclusion was based on high quality ribo-seq data.

To support the claim made in Figure 4 and demonstrate that the increase in mRNA abundance of ECT8 & m6A targets in the ect8-1 mutant is specifically due to decreased decay rates without an increased transcription, perhaps quantification of pre-mRNA could be made for some representative genes. It would be more convincing if there was no change in pre-mRNA detected between the two genotypes while the total poly(A)+ mRNA levels were increased in ect8-1 mutant

4. Localization of ECT8 in processing bodies of visible sizes
The conclusion that ECT8 is localized to processing bodies was drawn from transient over-expression of ECT8-GFP in the protoplast system, which does not reflect the physiological ECT8 concentration within the cells. I wondered if this would still be the case if the protein is stably expressed under its endogenous promoter.

5. The oligo primers sequences used for cloning and generation of transgenic lines should have been provided.

Minor comments:

1. Figures 1D and 1E: how was statistical analysis performed with two biological replicates?
2. For Supplemental Figure S1A, in addition to the conserved region with 3 or 4 tryptophan residues, a diagram comparing the protein domains/motifs would provide some insight about the proteins under comparison

Line 138: Supplemental Figure S2, A-C, not Supplemental Figure S1, A-C. Line 139: Supplemental Figure S2D, not Supplemental Figure S1D. It would be helpful to the reader if the location of genotyping primers used for Figure S2D was demonstrated in the diagram of Figure s2A.
Line 218: Figure 4E
Line 449: transcribed
Line 508: Would it be more appropriate to use poly(A)+ RNA instead of mRNA? There was no information on how total RNA isolation and polyA selection were performed and what tissue was used.
Line 548: at the same time

Line 550: presented, typo?
Line 557: (1.54d), what does this mean?
Line 566: No DNAse treatment before cDNA synthesis?
Line 600: after that?
Line 608: poly(A)+ RNA-seq: please provide more information on how sequencing of the RNA-seq libraries was performed.

Editorial comments:
The manuscript is wordy, especially in the Results section, with overly long introductory sentences. It should be more concise.

Point-by-point responses

Reviewer #1 (Comments for the Author):

The manuscript "m^{6}A reader ECT8 functions as an abiotic stress sensor by accelerating mRNA decay in Arabidopsis" by Cai et al. describes the role of m^{6}A reader ECT8 under salt stress in Arabidopsis. The authors show that ECT8 binds to m^{6}A modified RNA and the target RNAs have variable lifetime in the ect8-1 mutant. Overall the results and the methods implemented to get said results are satisfactory. m^{6}A and several m^{6}A related proteins have now been shown to be involved in stress regulation. Just recently ECT1 has been shown to be involved in Arabidopsis stress response (Lee et al 2023). However, in the current manuscript authors do show the binding of ECT8 to RNA and provide important datasets like FA-CLIP which helps with the novelty of research.

Below you will find my comments, questions and suggestions (in order of appearance in the MS):

Response: We thanks for the positive comments and thorough review of our work. The specific, point-by-point responses to your comments are addressed below.

1. The figure numbers in line 138 and 139 are not correct. There is no figure S1D

Response: We apologize for the written mistakes in the manuscript, which have been corrected in the revised manuscript.

2. In the section "ECT8 is highly expressed in flowers, roots, and leaves and localized in cytoplasm" I would like to ask why the authors did not use a fluorescent tagged ECT8 line (they use ECT8:GFP and YFP constructs in later sections) to show the localization of ECT8. I do not have anything against the GUS reporter assay, however, as we know, ECT8 has a N-terminal disordered region and according to authors is localized in P-bodies, it would be far more interesting if the authors could show the localization of ECT8 in planta under control and stress conditions.

Response: We appreciate your kind suggestion. Due to time limitation, we have not yet acquired transgenic materials for stable expression of ECT8 fused with a fluorescent protein such as GFP or YFP driven by its native promoter. Alternatively, we separated nuclear and cytoplasmic fractions and measured ECT8 protein by western blotting, showing that ECT8 localizes in cytoplasm (Supplemental Figure S3C). We also transiently expressed ECT8 and P-body markers, DCP1 and DCP5, in protoplast and showed that ECT8 co-localizes with P-bodies in *Arabidopsis* (Figure 5A; Supplemental Figure S8B).

Minor: line 147 should be ECT8prom:GUS instead of ECT8:GUS

Response: Corrected.

3. In the section "Salt stress induces a significant increase in transcription and expression of ECT8" authors monitor the level of ECT8 mRNA and pre-mRNA under stress. I have a few issues in this section:

a. How did the authors make the distinction between mRNA and pre-mRNA?

Response: The distinction between mRNA and pre-mRNA was made through our primer design strategy, which was conducted on the NCBI website. For the quantification of mRNA via RT-qPCR, we utilized primers situated at the exon-exon junction. In contrast, for pre-mRNA detection, we employed primers located within the intronic region. We have updated all primer information in the revised Supplemental Table 1. Additionally, we used nuclear run-on assay to monitor the transcription accumulation of *ECT8* under salt stress (Figure 2B), with results consistent with those observed in Figure 2A.

b. The primers listed in the supplementary data table are same for both mRNA and pre-mRNA. As far as I know, it is impossible to distinguish these based solely on primers.

Response: Thank you for pointing this out. We apologize for the oversight in our Supplemental Table 1, where we inadvertently omitted the primers used for

pre-mRNA detection. We have now revised this error and included the relevant information.

c. The methods section for RT-qPCR states "a minimum of two biological replicates". This is not clear. I suggest authors have 3 replicates at the least in every experiment.

Response: Thanks for your suggestion. We have conducted the RT-qPCR experiment again, implementing at least three biological replicates in the process. However, due to the numerous time points (0, 15 min, 30 min, 60 min, 120 min) required for both the WT and *ect8-1* mutant in the RNA stability assay under mock and salt stress, it was challenging to handle parallelly these samples with three biological replicates. Therefore, we performed the RNA stability assay with only two biological replicates. We have made this change in the method section of RT-qPCR. Additionally, we have added the detail about the sample replicates in each figure legend in the revised manuscript.

d. In the western blots showing ECT8 protein levels, what might be the reason behind two bands? Which band is ECT8? According to Uniprot ECT8 has a MW of 59.5kDa, however the bands shown are more likely around 70kDa.

Response: We appreciate your observation regarding the ECT8 protein bands. Upon comparison with other WT samples, we found that upper bands corresponding to ECT8 was caused by non-specific binding of the *Arabidopsis* protein with our used FLAG antibody. Therefore, to reduce the influence of non-specific protein bands in western blotting assay, we have switched to a different anti-FLAG antibody (MCE, Catalog#: HY-P80111) and observed a clear and single band of ECT8 around the 65 kDa (Figure 2C; Supplemental Figure S3C). The molecular weight of ECT8 plus 3×FLAG Tag is around 63 kDa. The observed band is very close the predicted molecular weight of ECT8-3×FLAG.

e. Authors should quantify the bands using ImageJ or similar software since (at least to my eyes) it seems that the band intensity of Mock at 0H is similar to that of treated

at 5H. I would assume that at 0H both should have similar intensity bands.

Response: We have repeated the experiment to track the temporal changes in ECT8 protein expression levels at the indicated time point (0 h, 2 h and 4 h) under both mock conditions and 150 mM NaCl stress (Figure 2C). To minimize the impact of exposure time, non-specific binding bands, and ensure accurate quantification, we loaded protein samples on the same SDS-PAGE gel and quantified protein abundance using the newly purchased FLAG antibody along with ImageJ software for analysis. In the revised Figure 2C, compared to the loading control ACTIN, the abundance of ECT8 protein remained relatively unchanged under mock conditions. However, its protein level significantly increased under salt treatment. Please see the revised Figure 2C.

4. In the section "ECT8's m^{6}A binding capability is required for responding to salt stress" In the methods related to this section, please specify how many root lengths were measured.

Response: For our root length measurement experiment, we have added "n = 3 biological replicates, each with at least 10 seedlings" in the figure legend (Fig. 2G). These 3-day-old seedlings grown on 1/2 MS plates were transferred to a vertical 1/2 MS plate with or without 100 mM NaCl (Figure 2F). After cultivating them for an additional four days, we photographed these seedlings and individually measured the root length using ImageJ (Figure 2G).

5. In the section "ECT8 binds to 3' UTR of mRNA with m^{6}A modification under normal and salt stress conditions"

a. As mentioned earlier, only 2 biological replicates were used for FA-CLIP and m^{6}A-seq. Since these are novel data they will be strengthened by the presence of 3 replicates. Authors should add the 3rd replicate and show proof of replicability.

Response: Thanks for your suggestion. Considering the operational challenges associated with FA-CLIP, we have only added a third biological replicate for the m^{6}A-seq and RNA-seq. To demonstrated the reliability of FA-CLIP, m^{6}A-seq, and

RNA-seq, we have presented the data in the form of heatmaps and PCA analysis (Supplemental Figure S5, A and B; Supplemental Figure S6A).

b. The language in this section and the following sections regarding the transcripts that are bound by ECT8, bound by ECT8 and have m^{6}A and not bound by ECT8 needs to be clarified. At present it is not clear when authors talk about peaks, transcripts or genes. I suggest consistency in text and figures about whether they depict transcripts or genes associated to those transcripts.

Response: Sorry for the confusion. In the revision, we have renamed the names: For genes or transcripts, ECT8 targets that bound by ECT8 was names as ECT8 targeted genes; ECT8 & m^{6}A targets that bound by ECT8 and modified with m^{6}A was termed as ECT8 & m^{6}A targeted genes; non-targets were named as non-ECT8 targeted genes. For peaks, peaks bound by ECT8 were names as ECT8-binding sites; peaks bound by ECT8 and modified with m^{6}A were named as ECT8 & m^{6}A binding sites.

c. In order to verify the quality of m^{6}A-seq a metaplot to show the m^{6}A peak distribution should be added to supplementary dataset.

Response: The metagene profile of m^{6}A-seq has been illustrated in Figure S5E.

6. In section "Elevated ECT8 protein enhances its binding capacity to m^{6}A-modified mRNAs under salt stress"

a. Line 217-218: "......stress did not disturb ECT8's binding positions (Figure E)" Figure E what?

Response: We apologize for the incorrect labeling of the figures, it has now been corrected to Figure 3E.

b. Regarding figure 3G and 3H, it is unclear to me what 'Peak enrichment fold' means? How is this metric calculated and how does it corelate to ECT8's binding efficiency? Please clarify in methods.

Response: Sorry for the confusion. We have changed “peak enrichment fold” into

"ECT8's binding ability", which was calculated by the enrichment fold of ECT8's binding peaks divided by the enrichment fold of ECT8m's binding peaks. The enrichment fold of ECT8 and ECT8m's binding peaks in FA-CLIP were calculated by the *macs2 callpeak* software. We have added the clarity in the Methods section of our revised manuscript.

c. In figure 3H, it seems that m^6A levels go down after salt stress. However, in the text authors write "significantly increased binding affinity to ECT8 & m^6A common targets, even in the absence of a substantial increase in overall m^6A abundance" line 225-226. If the m^6A levels go down after salt stress, what is ECT8 binding to? This is also where the metaplots for m^6A-seq could come in handy.

Response: In Figure 3H, we took m^6A spike-in to determine the m^6A levels of the transcripts that ECT8 binds to under mock and salt stress conditions using the method reported (Wei, et al., *Science*, 2022, 376(6596), 968-73). The data revealed that there was no significant change in m^6A levels following a 4-hour high salt treatment. Further, our mass spectrometry analysis (Figure S5H) confirmed that there was no significant change in the overall m^6A levels before and after a 4-hour 150 mM NaCl treatment. This finding corroborates the result of other studies (Hu, et al., *Plant J*, 2021, 106: 1759-75), which also reported that there is no significant increase of m^6A level even under 6-hour 150 mM NaCl treatment.

7. In section "ECT8 promotes m^6A-modified mRNA decay through direct interaction with DCP5 in P-bodies"

a. Authors use DCP1 to establish that ECT8 localizes in P-bodies. However, every experiment after that uses DCP5. Why?

Response: Thanks for your suggestion. In the revision, we have performed the experiment to show that ECT8 colocalizes with DCP5 in P-bodies (please see Figure 5A). At the beginning, we used DCP1 to confirm ECT8's localization in P-body. After that, we investigated how ECT8 gets into P-body, and we found ECT8 directly interacts with DCP5. Thus, we used DCP5 in every experiment after that. In the

revised manuscript, we have showed ECT8 colocalizes with DCP5 and DCP1 in P-bodies, respectively (Figure 5A and Supplementary Figure S8B).

b. Multiple proteins have been shown to be in P-Bodies, XRN4, VCS, AGO1, etc. The authors did not show if ECT8 interacts exclusively with DCP5 or whether it also interacts with these other proteins? A wider Y2H screen could identify more interactors providing more insight.

Response: Thanks for your suggestion. In the revision, we have conducted an expanded Y2H screening to explore the interaction between ECT8 and a selection of proteins including DCP1, DCP2, DCP5, NOT1, XRN4, VCS, and AGO1. The results showed that ECT8 does not interact with most of these proteins, except for VCS and DCP5 (Supplementary Figure S8C). DCP5, along with associated proteins like VCS, DCP1, and DCP2, primarily functions in removing the protective 5' cap structure from mRNA molecules within P-bodies, ultimately leading to mRNA degradation. The identified interaction between ECT8 with VCS lends further support to the conclusion that ECT8 participates in the degradation process of transcripts at their 5' end within P-bodies.

c. Since AGO1 is also a part of P-bodies, could miRNA mediated mRNA decay also play a part in this? It is known that m^6A also affects miRNA biogenesis (Bhat et al., 2020), how does that affect authors hypothesis.

Response: In the revision, we have performed Y2H assay to show no interaction between ECT8 and AGO1 (Supplementary Figure S8C). It is well known that m^6A methylation occurs in pri-miRNAs and influences miRNA biogenesis in *Arabidopsis*, but no m^6A methylation is present in mature miRNA (Bhat, et al., *Proc Natl Acad Sci U S A*, 2020, 117(35), 21785-95). Considering that ECT8 localizes in the cytoplasm and our FA-CLIP sequencing data reveals that there is no miRNA bound by ECT8 under both mock and salt stress conditions (Additional Table 1). Thus, we think that ECT8 does not function in the biogenesis of miRNA or miRNA-mediated mRNA decay.

Annotation of ECT8 targets (Mock)		Annotation of ECT8 targets (Salt)	
Transcript type	Targets numbder	Transcript type	Targets numbder
mRNA	7019	mRNA	8543
lncRNA	16	lncRNA	15
ncRNA	15	ncRNA	18
nontranslating CDS	4	nontranslating CDS	8
miRNA	0	miRNA	0
snoRNA	0	snoRNA	1
snRNA	0	snRNA	3
rRNA	0	rRNA	0
tRNA	0	tRNA	0

Additional Table 1. Annotation of ECT8 targeted genes under mock and salt stress. All genes are annotated using Araport11 from TAIR website (https://www.arabidopsis.org/)

8. In section "ECT8 destabilizes negative regulators of salt stress response for enhancing salt stress tolerance" line 352-353: "The results showed that they underwent more rapid degradation in ect8-1 compared to WT under both normal and salt stress conditions" I think the authors have misstated the results. Since the figures show stabilization in ect8 mutant.

Response: Sorry for the mistake. We have changed it into “they underwent slower degradation in *ect8-1* compared to WT under both normal and salt stress conditions”.

Reviewer #2 (Comments for the Author):

This article focuses on N^6-methyladenosine (m^6A), the most prevalent mRNA modification in eukaryotes, including plants, and its roles in various biological processes such as plant growth, development, and stress response. It highlights the identification of ECT8 as an m^6A reader protein crucial for the plant's response to salt stress. ECT8 enhances the degradation of target transcripts by interacting with the decapping protein DCP5 within P-bodies, a process particularly important under various environmental stresses. The study observes a significant increase in ECT8 expression under such conditions, and specifically in response to salt stress, both at

the transcript and protein levels. This increase in ECT8 leads to more effective binding to m^{6}A-modified mRNAs, promoting their degradation, especially those negatively regulating salt stress response. The findings demonstrate ECT8's role as a sensor of abiotic stresses, aiding in mRNA decay to maintain homeostasis and improve stress tolerance in plants. These insights contribute to the understanding of epitranscriptomic gene regulation and have potential applications in breeding more resilient crops amid changing environmental conditions.

Believing that the article will be read and will be cited, I have a few remarks for the authors to implement corrections or possibly respond to them.

Response: We express our gratitude to you for your positive remarks and comprehensive evaluation of our work. We have provided specific responses to each of the comments in the following sections.

1) I believe that there is a particular lack in Fig S3 of results that show the preferential localization of ECT8 in the cytoplasm using some bioimaging techniques. Ideally, this should be shown for different plant tissues for comparison.

Response: Owing to time limitation, we have not obtained transgenic materials that stably express ECT8 fused with fluorescent proteins under native promoter yet. Instead we reperformed nuclear-cytoplasmic fractionation experiment to blot ECT8 protein with a newly ordered anti-FLAG antibody which has better specificity. The results clearly showed that ECT8 is primarily localized in the cytoplasm (Supplemental Figure S3C). The transiently expression of ECT8-GFP and P-body marker, DCP1-mCherry and DCP5-mCherry, in the *Arabidopsis* protoplast showed that ECT8 colocalizes with DCP1 and DCP5 in P-bodies with condensates, which is present in cytoplasm (Figure 5A; Supplemental Figure S8B).

2) Please correct the reference to Fig in line 218 of the text (it is Figure E); it should be 3E.

Response: We sincerely apologize for the mislabeling of the figures. The error has been revised, and the correct label is now Figure 3E.

3) The image in Figure 5A should include the localization of the cell nucleus. This is a note in addition to point 1. Regarding this image, a better marker for P bodies would be DCP2 or of course DCP5, if the authors have the possibility to show these colocalizations with ECT8. However, if it is not feasible to make this correction to the manuscript within the necessary timeframe, the localization of DCP1 is acceptable.

Response: Thanks for the suggestion. In the revision we have transiently expressed ECT8-GFP and P-body marker DCP5-mCherry in the *Arabidopsis* protoplast and showed that ECT8 colocalizes with DCP5 in the puncta (Figure 5A), further reinforcing the evidence of ECT8's localization in P-bodies.

4) In the diagram (Figure 7) summarizing the study results, the site where mRNA degradation occurs through the interaction of ECT8 with DCP5 is indicated as P bodies. This is likely, but since the authors have not presented direct evidence of such localization of this process in P bodies, at this stage of the research it is only a hypothesis, and this should at least be mentioned in the figure description.

Response: Thanks for the suggestion. In the revision, we redraw the diagram (Figure 7), and stated it is hypothesis in text: "Considering that the number of P-bodies increases in *Arabidopsis* under salt stress conditions (Steffens, et al., *PLoS Biol*, 2015, 13(7): e1002188), we hypothesize that the increased abundance of ECT8 induced by salt stress recruits more m^{6}A-modified mRNAs to get into more P-bodies. As a consequence, this amplifies the degradation of ECT8 bound mRNAs, including negative regulators of salt stress response, ultimately enhancing salt stress tolerance."

5) It's not required, but I really feel the absence of an experiment to find protein partners of ECT8. I am referring to the proteomic analysis of immunoprecipitates (IP-LC-MS/MS). This would provide additional confirmation for DCP5 beyond the Yeast two-hybrid (Y2H) assay, as well as addressing what the authors discuss in lines 420-423.

Response: Thanks for your kind suggestion. In fact, we have conducted protein

IP-LC-MS/MS experiments on ECT8-FLAG/*ect8-1* materials. Due to the low protein level of DCP5, only a small amount of DCP5 protein peptides were detected in our IP group - two and five peptides belonging to DCP5 protein were identified in the two biological replicates, respectively. Thus, we only put Y2H and BiFC results that confirming the interaction between DCP5 and ECT8 in the manuscript.

Reviewer #3 (Comments for the Author):

The manuscript by Cai, Tang and Song et al. reports the function of the m^6A *reader ECT8 in Arabidopsis. The authors demonstrate ECT8 plays a role in salt stress tolerance and contributes to mRNA decay through its interaction with the decapping activator DCP5. Overall, I find the work interesting and novel, with genetic evidence and a series of genome-wide omics data to support the authors' conclusion. The study is expansive and provides new findings related to the regulation of mRNA fates by an* m^6A *reader.*

Please see below my specific comments and questions intended to improve the quality of the manuscript.

Response: We're truly thankful for your kind comments and detailed review of our work. The specific, point-by-point responses to your comments are addressed below.

Major comments:

1. Western blot images of FLAG-tagged ECT8

The western blot images of FLAG-tagged ECT8 (Figure 2B) showed two discreet bands with anti-FLAG detection. What would be an explanation of for this phenomenon? This is consistent with the data in Supplemental Figure S3C. it seems to me that the total protein fraction showed ECT8 detection with two sizes, while the cytosolic fraction contained the band of smaller size and the nuclear fraction with the larger size. I am not convinced that ECT8 is preferentially cytosolic. The anti-FLAG panel of Supplemental Figure S3C should be cropped in a way that the upper band is not omitted.

Response: Thanks for your kind suggestion. We appreciate your observation regarding the ECT8 protein bands. Upon comparing with other wild-type plant samples, we identified that the upper bands corresponding to ECT8 in our initial submission was caused by non-specific binding of the *Arabidopsis* protein with our used anti-FLAG antibody. Therefore, to reduce the influence of non-specific protein bands in western blotting assay, we have purchased a different anti-FLAG antibody (MCE, Catalog#: HY-P80111) and observed a clear and single band of ECT8 around the 65 kDa (Figure 2C; Supplemental Figure S3C).

Additionally, we have also repeated the time-course experiment to track the protein expression level of ECT8 under 150 mM NaCl stress using new antibody (Figure 2C). Our results reveal that the protein level of ECT8 increases during salt stress.

2. Comparison of ECT8-binding sites and m^6A *peaks*

The authors compared m^6A*-seq with FA-CLIP data under both mock (control) and salt stress conditions. This showed that the majority of ECT8 targets have* m^6A *marks, which makes sense. However, I find the number labeling format for Figures 3A and 3B confusing. I don't understand why there were two numbers for the overlaping region of the Venn diagrams. In Figure 3A, for example, it would be understandable if there were 16,177 m6A targets and 7,592 ECT8 targets detected, with the overlap of 5,656. But the authors also labelled 5,684* m^6A *targets and 6,841 ECT8 targets in the overlapping region that do not add up to 5,656. Please clarify why this was the case. More explanation in the text and/or figure caption would be helpful.*

Response: Sorry for the confusion. In the revision, we have changed “ECT8 & m^6A targets” into “ECT8 & m^6A targeted genes” to make clearer and more understandable. We utilized the *bedtools intersect* algorithm to draw the Venn diagrams. In the revision, we have done m^6A-seq using triplicated biological samples which makes the little different on the m^6A peaks numbers. Figure 3A, for instance, 5,532 m^6A peaks were overlapped with ECT8-binding sites, while 6,535 ECT8-binding sites were overlapped with m^6A peaks. The number of genes containing the overlapped 5,532

m^6A peaks and 6,535 ECT8-binding sites were 5,479, which was named as "ECT8 & m^6A targets" in the last version, but now was changed into "ECT8 & m^6A targeted genes".

I am also confused why the number of ECT8 & m^6A binding sites presented in Figure 3F did not correspond with the number in Figures 3A and 3B. My understanding is that there were 5,656 ECT8 & m6A targets under mock condition and 7,075 under salt stress conditions. However, the number in Figure 3F was 6,841 and 8,119, respectively. Was this because Figures 3A and 3B referred to the number of genes but 3F referred to the number of sites? Whatever the case, the analysis and comparison should be described more clearly in the text and figure captions. The distinction between targets and sites should be clearly defined and presented systematically. Perhaps replacing Figure 3F with Figure S5D would make it easier for the reader to follow.

Response: Sorry for the confusion. As suggested, we have replaced Figure 3F with Figure S5F in the revised manuscript. Here is the explanation about Figure 3A and 3B with the related Figure 3F (Figure S5F in the revision). Figure S5F is overlapping ECT8-binding sites modified with m^6A (ECT8 & m^6A binding sites) between normal (6,535 sites) and salt condition (8,240 sites). The number of ECT8 & m^6A binding sites under normal condition (6,535 sites) is shown in Figure 3A, which shows 6,535 ECT8 binding sites were overlapped with m^6A peaks. Similarly, the number of ECT8 & m^6A binding sites under salt condition (8,240 sites) is shown in Figure 3B, which shows 8,240 ECT8 binding sites were overlapped with m^6A peaks. We have revised the description clearer in the text and figure legends.

By comparing Figure 3A with Figure 3B, there was increase of the ECT8 & m^6A targets from 5,656 targets under mock condition to 7,075 under salt condition. Is there an enrichment for specific biological functions from those 1,419 targets that was increased by salt stress?

Response: Thanks for your suggestion. In our manuscript, we conducted a GO analysis on the common ECT8 & m^6A targeted genes under both mock and salt stress

conditions (Figure 3I). In detail, our Venn diagram revealed 1,381 ECT8 & m^6A targets specific to the Mock condition (we called mock specific ECT8 & m^6A targeted genes), 3,172 ECT8 & m^6A targets specific to the salt condition (we called salt specific ECT8 & m^6A targeted genes) and 4,098 ECT8 & m^6A targets common to both conditions (we called common ECT8 & m^6A targeted genes) (Figure 3F).

In response to your suggestion, we have taken mock specific ECT8 & m^6A targeted genes and salt specific ECT8 & m^6A targeted genes to conduct GO analysis (Supplementary Figure S5, I and J in this revision). The results were obtained using DAVID program (https://david.ncifcrf.gov/tools.jsp) and further visualized using R packages *ggplot2*. It is evident that the mock-specific, common, and salt-specific ECT8 & m^6A targeted genes are all enriched in pathways related to gene expression regulation and RNA metabolism. Interestingly, we observed a distinct enrichment in the GO pathways related to osmotic changes and salt stress response in both common and salt-specific ECT8 & m^6A targeted genes.

The color scheme for Figure 3F should be changed from blue-green to blue-red as in Figure 2C, as this was a comparison of the mock and salt stress conditions. I understand that GO enrichment analysis was performed on ECT8 & m^6A common (mock VS control) targets and presented in Figure 3I, but what about those that are unique to either mock or salt stress conditions?

Response: Thanks for the suggestion. We have changed the color of Figure 3F (Figure S5F in the revised manuscript) into blue-red. As responded in last question, we have performed GO analysis for the unique ECT8 & m^6A targeted genes specific to mock and salt, please see Supplementary Figure S5, I and J in the revision.

Lines 224-228: The author wrote, "It has been observed that under salt stress, ECT8 exhibits a significantly increased binding affinity to ECT8 & m^6A common targets, even in the absence of a substantial increase in overall m^6A abundance or the expression levels of m^6A writer subunits and erasers (Figure 3, G and H; Supplemental Figure S4, B and C; Supplemental Figure S5E)." In fact, Figure 3H

indicates that m^6A level was significantly decreased for ECT8 & m^6A common targets under salt stress conditions. The text should be corrected accordingly.

Response: In the revision, we performed m^6A-seq using triplicated biological samples and employed m^6A spike-in to calculate the m^6A levels of common ECT8 & m^6A targeted genes (mock & salt) following the analysis method for m^6A level used in the literature (Wei et al., *Science*, 2022, 376(6596), 968-73). The results showed that the m^6A level of common ECT8 & m^6A targeted genes were not significantly altered upon 4-hour salt treatment compared to normal (Figure 3H). Furthermore, our mass spectrometry analysis (Supplemental Figure S5H) confirmed that there was no significant increase in the overall m^6A levels pre and post salt stress treatment, consistent with the findings of other studies (Hu et al., *Plant J*, 2021, 106: 1759-75), which also reported that there is no significant increase of m^6A level even under 6-hour 150 mM NaCl treatment. Thus, in the revised manuscript we have changed the sentence into "ECT8 exhibits a significantly increased binding ability towards common ECT8 & m^6A targeted genes under salt stress condition compared to normal condition, although overall m^6A abundance varies negligibly between both conditions".

3. mRNA lifetime sequencing & ribosome-sequencing

There was no indication on how many replicate samples were used for mRNA half-life sequencing, and what the statistical model/criterion was used to compare the mRNA half-life between the genotypes. This makes it impossible to determined the quality of the mRNA decay data and whether the authors' interpretation of the data was valid. The data in Figure 4B were presented as cumulative fraction of relative mRNA half-live between ect8-1 mutant and the wild type, without any other information provided to allow for an assessment of the efficiency of cordycepin treatment. What is the number of seedlings used in the cordycepin treatment experiment? Also the libraries was made with poly(A) selection, which overlooked mRNA decay intermediates without poly(A) tails.

Response: We have added the detailed information in the method of "mRNA lifetime

sequencing". In brief, we organized this experiment into five groups, each representing a different time point. Each group consisted 7-d-old WT and *ect8-1* seedlings (ten plants for each genotype). We conducted two biological replicates for mRNA lifetime sequencing and ensure the reliability of our sequencing data (Supplemental Figure S6B).

Regarding the efficiency of our cordycepin treatment, we observed that the median mRNA half-life for WT in our study is approximately 101 minutes (Additional Figure 1A). This finding aligns closely with previously reported data that shows 107 minutes of the median mRNA half-life for WT (Sorenson et al., *Proc Natl Acad Sci U S A*, 2018, 115(7), E1485-94). Moreover, we selected two typical known transcripts - *AT5G46020* with a long half-life and *AT2G41010* with a short half-life - for evaluating the quality of our sequencing data. Our results exhibit the mRNA half-life features of these two transcripts (Additional Figure 1B). This evaluation allows us to validate the feasibility of our procedure for measuring mRNA half-life. Consequently, we have confidence in the effectiveness of our cordycepin treatment approach.

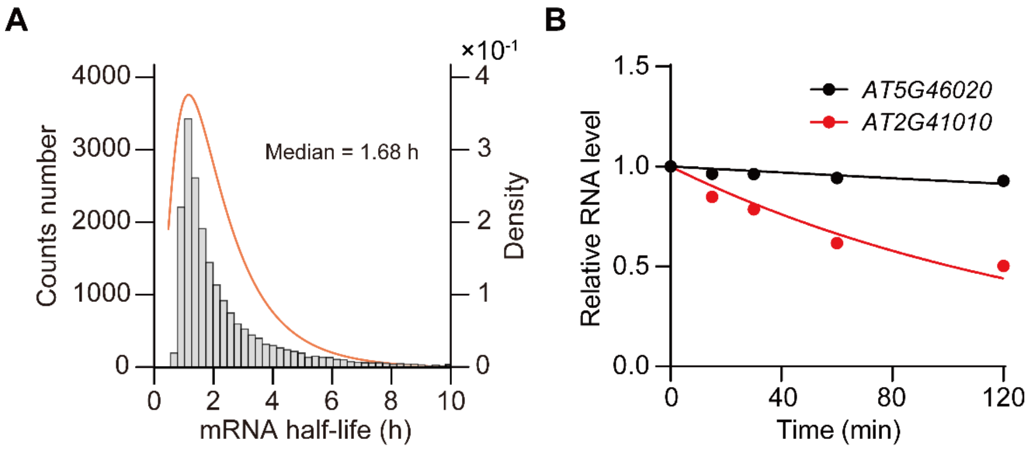

Additional Figure 1. (A) The distribution of mRNA half-life in WT; (B) The degradation curve from two typical transcripts.

We appreciate your point about the poly(A) selection during library preparation, which could potentially overlook mRNA decay intermediates without poly(A) tails.

However, our primary focus in this study was on the mRNAs, which represent the majority of ECT8 targeted genes (Supplemental Figure S5C). Nonetheless, we acknowledge that whether ECT8 influences the decay rate of transcripts without poly(A) tails is also important, and we will consider this in our future investigations. Thank you again for your valuable feedback.

This was also the case for the quality of ribosome-sea data. The authors made a conclusion that ECT8 mutation has no effects on translation efficiency of ECT8 & m^6A targets, but without providing sufficient evident that the conclusion was based on high quality ribo-seq data.

Response: In response to your concern, we have thoroughly examined our ribosome-seq data using heatmaps that clearly demonstrate the consistency and reliability of our data (Supplemental Figure S7B). The heatmaps, we believe, provide substantial evidence to support our conclusion that ECT8 does not significantly affect the overall translation efficiency under normal condition. We appreciate your feedback and hope that this addresses your concern.

To support the claim made in Figure 4 and demonstrate that the increase in mRNA abundance of ECT8 & m^6A targets in the ect8-1 mutant is specifically due to decreased decay rates without an increased transcription, perhaps quantification of pre-mRNA could be made for some representative genes. It would be more convincing if there was no change in pre-mRNA detected between the two genotypes while the total poly(A)$^+$ mRNA levels were increased in ect8-1 mutant.

Response: Thanks for your suggestion. In the revision, we have RT-qPCR to assess the expression levels of pre-mRNA of the representative ECT8's targeted genes (*PPRT1*, *MSI1*, *BIL1*, *ENGD1*). Our findings indicate that the absence of ECT8 does not significantly affect the abundance of pre-mRNA of these genes under both normal and salt conditions (Supplemental Figure S10).

4. Localization of ECT8 in processing bodies of visible sizes

The conclusion that ECT8 is localized to processing bodies was drawn from transient over-expression of ECT8-GFP in the protoplast system, which does not reflect the physiological ECT8 concentration within the cells. I wondered if this would still be the case if the protein is stably expressed under its endogenous promoter.

Response: We appreciate your comment regarding the subcellular localization of ECT8 in P-bodies. Due to the time limitation, we have not obtained transgenic materials that stably express ECT8 fused with fluorescent proteins under native promoter yet. Instead, we have transiently expressed ECT8-GFP and P-body marker DCP5-mCherry in *Arabidopsis* protoplast and showed that ECT8 colocalizes with DCP5 in the puncta (Figure 5A), further reinforcing the evidence of ECT8's localization in P-bodies. We also perform a expand YTH screen to find ECT8 not only interacts with DCP5 but also VCS (Supplemental Figure 8C). DCP5, along with associated proteins like VCS, DCP1, and DCP2, primarily functions in removing the protective 5' cap structure from mRNA molecules for degradation within P-bodies (Xu, et al., *Plant Cell*, 2009, 21(10), 3270-9). The identified interaction between ECT8 with VCS lends further support to the conclusion that ECT8 participates in the degradation process of transcripts at their 5' end within P-bodies.

5. The oligo primers sequences used for cloning and generation of transgenic lines should have been provided.

Response: We apologize for this omission. Complete primer details have now been added to Supplemental Table 1.

Minor comments:

1. Figures 1D and 1E: how was statistical analysis performed with two biological replicates?

Response: Data in (D) and (E) are analyzed by two-sided t test and presented as means ± SE, n = 3 biological replicates × 3 technical replicates, ***$P < 0.001$ and ****$P < 0.0001$. We have added it in the legend of Figure 1.

2. For Supplemental Figure S1A, in addition to the conserved region with 3 or 4 tryptophan residues, a diagram comparing the protein domains/motifs would provide some insight about the proteins under comparison.

Response: Thank you for your suggestion. In the revised manuscript, we have compared the YTH domains among ECT8, ECT2, and human YTHDF1-3, please see Supplemental Figure S1A.

Line 138: Supplemental Figure S2, A-C, not Supplemental Figure S1, A-C. Line 139: Supplemental Figure S2D, not Supplemental Figure S1D. It would be helpful to the reader if the location of genotyping primers used for Figure S2D was demonstrated in the diagram of Figure s2A.

Response: We appreciate your attention to detail and all these errors are corrected. We also illustrated the location of primers used for genotyping in Supplemental Figure S2A.

Line 218: Figure 4E

Line 449: transcribed

Response: All these have been corrected.

Line 508: Would it be more appropriate to use poly(A)+ RNA instead of mRNA? There was no information on how total RNA isolation and polyA selection were performed and what tissue was used.

Response: Thanks for the suggestion. We have changed mRNA into poly(A)$^+$ RNA. The method related with total RNA extraction and poly(A)$^+$ RNA isolation was in the method of "poly(A)$^+$ RNA-seq". In brief, total RNA was extracted from *Arabidopsis* seedlings using TRIzol™ Reagent (Invitrogen). Subsequently, total RNA was then employed to isolate poly(A)$^+$ RNA using oligo(dT)$_{25}$ Dynabeads (Thermo Fisher Scientific) follow by the procedure.

Line 548: at the same time

Response: Sorry for this mistake, and it has been corrected now.

Line 550: presented, typo?

Response: It has been corrected in our revised manuscript.

Line 557: (1.54d), what does this mean?

Response: In this context, '(1.54d)' indicates that the version 1.54d of ImageJ was used. We have changed it into “version 1.54d”.

Line 566: No DNAse treatment before cDNA synthesis?

Response: Thanks for your query regarding our cDNA synthesis process. We used the PrimeScript™ RT Reagent Kit with gDNA Eraser (Perfect Real Time) (RR047A) from TaKaRa for our experiments. The protocol for this kit begins with the treatment of 1μg of total RNA with gDNA eraser to remove any potential DNA contamination. The subsequent reverse transcription process was also conducted following the protocol provided by the kit. The resulting cDNA was appropriately diluted before being used for qPCR analysis. We have added the information into the method of “RT-qPCR”.

Line 600: after that?

Response: It has been revised.

Line 608: poly(A)+ RNA-seq: please provide more information on how sequencing of the RNA-seq libraries was performed.

Response: We have added the detailed information into the related methods. All the libraries of RNA-seq, m^6A-seq, FA-CLIP and Ribo-seq were sequenced on the Illumina NovaSeq 6000 platform with a paired-end model (PE150).

1st Revision - Editorial Decision10-Apr-2024

Dear Dr. Guifang Jia:

We have reviewed your revised manuscript entitled "m6A reader ECT8 functions as an abiotic stress sensor by accelerating mRNA decay in Arabidopsis." On the basis of this review, the board of reviewing editors would like to accept your manuscript for publication in The Plant Cell. This acceptance is contingent on revision based on the comments of our reviewers. In particular, please consider the following. A labeling error in the Legend of Supplemental Figure 8 was noticed. There are two E labels and thus F and G panels are mislabeled. Please correct this and resubmit the documents for final acceptance.

To submit your revised manuscript, click:

Link Not Available

If you have any questions about the revision submission procedures, please contact the Editorial Office Staff (tpc-submissions@aspb.org). If you cannot return the revised manuscript within 30 days, please let us know. Otherwise, we will assume that you have elected not to revise the manuscript and withdraw it.

Thank you very much for the privilege of reviewing this work. I look forward to receiving the next version.

Sincerely,

The Plant Cell Editorial Board

Please also note the following:

-The Plant Cell now requires authors to complete and submit an author revisions checklist upon submission of a revised manuscript. The aim of the checklist is to aid authors in preparing a high-quality manuscript, facilitate the review and assessment of revised manuscripts, and help to ensure that journal standards are maintained across the board. If your manuscript is accepted, the completed checklist will be published as supplemental material attached to the article online. Please download a copy of the checklist (pdf fillable form) at this link, for submission with your revised manuscript: https://tpc.msubmit.net/html/Author_Revisions_Checklist.pdf.

-Supplemental materials should be restricted to large datasets and tables, presentation of replicates, and validation of reagents, methods, or genotypes. Any data that are used to support the major claims must be in the main manuscript. Supplemental figure legends must indicate what figure in the main manuscript is supported by the supplemental data presented. Please justify how each of the supplemental figures meet the criteria.

-Sampling methods and the nature of "biological replicates" should be described precisely (i.e. different plants, parts of plants, pooled tissue, independent pools of tissue, sampled at different times, etc.), along with a clear description of and rationale for any statistical analyses conducted. The reader should know exactly what was sampled; what forms the basis of the calculation of any means and statistical parameters reported. This is also necessary to ensure that proper statistical analysis was conducted.

Point-by-point responses

Reviewer #1 (Comments for the Author):

The manuscript "m^{6}A reader ECT8 functions as an abiotic stress sensor by accelerating mRNA decay in Arabidopsis" by Cai et al. describes the role of m^{6}A reader ECT8 under salt stress in Arabidopsis. The authors show that ECT8 binds to m^{6}A modified RNA and the target RNAs have variable lifetime in the ect8-1 mutant. Overall the results and the methods implemented to get said results are satisfactory. m^{6}A and several m^{6}A related proteins have now been shown to be involved in stress regulation. Just recently ECT1 has been shown to be involved in Arabidopsis stress response (Lee et al 2023). However, in the current manuscript authors do show the binding of ECT8 to RNA and provide important datasets like FA-CLIP which helps with the novelty of research.

Below you will find my comments, questions and suggestions (in order of appearance in the MS):

Response: We thanks for the positive comments and thorough review of our work. The specific, point-by-point responses to your comments are addressed below.

1. The figure numbers in line 138 and 139 are not correct. There is no figure S1D

Response: We apologize for the written mistakes in the manuscript, which have been corrected in the revised manuscript.

2. In the section "ECT8 is highly expressed in flowers, roots, and leaves and localized in cytoplasm" I would like to ask why the authors did not use a fluorescent tagged ECT8 line (they use ECT8:GFP and YFP constructs in later sections) to show the localization of ECT8. I do not have anything against the GUS reporter assay, however, as we know, ECT8 has a N-terminal disordered region and according to authors is localized in P-bodies, it would be far more interesting if the authors could show the localization of ECT8 in planta under control and stress conditions.

Response: We appreciate your kind suggestion. Due to time limitation, we have not yet acquired transgenic materials for stable expression of ECT8 fused with a fluorescent protein such as GFP or YFP driven by its native promoter. Alternatively, we separated nuclear and cytoplasmic fractions and measured ECT8 protein by western blotting, showing that ECT8 localizes in cytoplasm (Supplemental Figure S3C). We also transiently expressed ECT8 and P-body markers, DCP1 and DCP5, in protoplast and showed that ECT8 co-localizes with P-bodies in *Arabidopsis* (Figure 5A; Supplemental Figure S8B).

Minor: line 147 should be ECT8prom:GUS instead of ECT8:GUS

Response: Corrected.

3. In the section "Salt stress induces a significant increase in transcription and expression of ECT8" authors monitor the level of ECT8 mRNA and pre-mRNA under stress. I have a few issues in this section:

a. How did the authors make the distinction between mRNA and pre-mRNA?

Response: The distinction between mRNA and pre-mRNA was made through our primer design strategy, which was conducted on the NCBI website. For the quantification of mRNA via RT-qPCR, we utilized primers situated at the exon-exon junction. In contrast, for pre-mRNA detection, we employed primers located within the intronic region. We have updated all primer information in the revised Supplemental Table 1. Additionally, we used nuclear run-on assay to monitor the transcription accumulation of *ECT8* under salt stress (Figure 2B), with results consistent with those observed in Figure 2A.

b. The primers listed in the supplementary data table are same for both mRNA and pre-mRNA. As far as I know, it is impossible to distinguish these based solely on primers.

Response: Thank you for pointing this out. We apologize for the oversight in our Supplemental Table 1, where we inadvertently omitted the primers used for

pre-mRNA detection. We have now revised this error and included the relevant information.

c. The methods section for RT-qPCR states "a minimum of two biological replicates". This is not clear. I suggest authors have 3 replicates at the least in every experiment.

Response: Thanks for your suggestion. We have conducted the RT-qPCR experiment again, implementing at least three biological replicates in the process. However, due to the numerous time points (0, 15 min, 30 min, 60 min, 120 min) required for both the WT and *ect8-1* mutant in the RNA stability assay under mock and salt stress, it was challenging to handle parallelly these samples with three biological replicates. Therefore, we performed the RNA stability assay with only two biological replicates. We have made this change in the method section of RT-qPCR. Additionally, we have added the detail about the sample replicates in each figure legend in the revised manuscript.

d. In the western blots showing ECT8 protein levels, what might be the reason behind two bands? Which band is ECT8? According to Uniprot ECT8 has a MW of 59.5kDa, however the bands shown are more likely around 70kDa.

Response: We appreciate your observation regarding the ECT8 protein bands. Upon comparison with other WT samples, we found that upper bands corresponding to ECT8 was caused by non-specific binding of the *Arabidopsis* protein with our used FLAG antibody. Therefore, to reduce the influence of non-specific protein bands in western blotting assay, we have switched to a different anti-FLAG antibody (MCE, Catalog#: HY-P80111) and observed a clear and single band of ECT8 around the 65 kDa (Figure 2C; Supplemental Figure S3C). The molecular weight of ECT8 plus 3×FLAG Tag is around 63 kDa. The observed band is very close the predicted molecular weight of ECT8-3×FLAG.

e. Authors should quantify the bands using ImageJ or similar software since (at least to my eyes) it seems that the band intensity of Mock at 0H is similar to that of treated

at 5H. I would assume that at 0H both should have similar intensity bands.

Response: We have repeated the experiment to track the temporal changes in ECT8 protein expression levels at the indicated time point (0 h, 2 h and 4 h) under both mock conditions and 150 mM NaCl stress (Figure 2C). To minimize the impact of exposure time, non-specific binding bands, and ensure accurate quantification, we loaded protein samples on the same SDS-PAGE gel and quantified protein abundance using the newly purchased FLAG antibody along with ImageJ software for analysis. In the revised Figure 2C, compared to the loading control ACTIN, the abundance of ECT8 protein remained relatively unchanged under mock conditions. However, its protein level significantly increased under salt treatment. Please see the revised Figure 2C.

4. In the section "ECT8's m^{6}A binding capability is required for responding to salt stress" In the methods related to this section, please specify how many root lengths were measured.

Response: For our root length measurement experiment, we have added "n = 3 biological replicates, each with at least 10 seedlings" in the figure legend (Fig. 2G). These 3-day-old seedlings grown on 1/2 MS plates were transferred to a vertical 1/2 MS plate with or without 100 mM NaCl (Figure 2F). After cultivating them for an additional four days, we photographed these seedlings and individually measured the root length using ImageJ (Figure 2G).

5. In the section "ECT8 binds to 3' UTR of mRNA with m^{6}A modification under normal and salt stress conditions"

a. As mentioned earlier, only 2 biological replicates were used for FA-CLIP and m^{6}A-seq. Since these are novel data they will be strengthened by the presence of 3 replicates. Authors should add the 3rd replicate and show proof of replicability.

Response: Thanks for your suggestion. Considering the operational challenges associated with FA-CLIP, we have only added a third biological replicate for the m^{6}A-seq and RNA-seq. To demonstrated the reliability of FA-CLIP, m^{6}A-seq, and

RNA-seq, we have presented the data in the form of heatmaps and PCA analysis (Supplemental Figure S5, A and B; Supplemental Figure S6A).

b. The language in this section and the following sections regarding the transcripts that are bound by ECT8, bound by ECT8 and have m^6A and not bound by ECT8 needs to be clarified. At present it is not clear when authors talk about peaks, transcripts or genes. I suggest consistency in text and figures about whether they depict transcripts or genes associated to those transcripts.

Response: Sorry for the confusion. In the revision, we have renamed the names: For genes or transcripts, ECT8 targets that bound by ECT8 was names as ECT8 targeted genes; ECT8 & m^6A targets that bound by ECT8 and modified with m^6A was termed as ECT8 & m^6A targeted genes; non-targets were named as non-ECT8 targeted genes. For peaks, peaks bound by ECT8 were names as ECT8-binding sites; peaks bound by ECT8 and modified with m^6A were named as ECT8 & m^6A binding sites.

c. In order to verify the quality of m^6A-seq a metaplot to show the m^6A peak distribution should be added to supplementary dataset.

Response: The metagene profile of m^6A-seq has been illustrated in Figure S5E.

6. In section "Elevated ECT8 protein enhances its binding capacity to m^6A-modified mRNAs under salt stress"

a. Line 217-218: "......stress did not disturb ECT8's binding positions (Figure E)" Figure E what?

Response: We apologize for the incorrect labeling of the figures, it has now been corrected to Figure 3E.

b. Regarding figure 3G and 3H, it is unclear to me what 'Peak enrichment fold' means? How is this metric calculated and how does it corelate to ECT8's binding efficiency? Please clarify in methods.

Response: Sorry for the confusion. We have changed “peak enrichment fold” into

"ECT8's binding ability", which was calculated by the enrichment fold of ECT8's binding peaks divided by the enrichment fold of ECT8m's binding peaks. The enrichment fold of ECT8 and ECT8m's binding peaks in FA-CLIP were calculated by the *macs2 callpeak* software. We have added the clarity in the Methods section of our revised manuscript.

c. In figure 3H, it seems that m^{6}A levels go down after salt stress. However, in the text authors write "significantly increased binding affinity to ECT8 & m^{6}A common targets, even in the absence of a substantial increase in overall m^{6}A abundance" line 225-226. If the m^{6}A levels go down after salt stress, what is ECT8 binding to? This is also where the metaplots for m^{6}A-seq could come in handy.

Response: In Figure 3H, we took m^{6}A spike-in to determine the m^{6}A levels of the transcripts that ECT8 binds to under mock and salt stress conditions using the method reported (Wei, et al., *Science*, 2022, 376(6596), 968-73). The data revealed that there was no significant change in m^{6}A levels following a 4-hour high salt treatment. Further, our mass spectrometry analysis (Figure S5H) confirmed that there was no significant change in the overall m^{6}A levels before and after a 4-hour 150 mM NaCl treatment. This finding corroborates the result of other studies (Hu, et al., *Plant J*, 2021, 106: 1759-75), which also reported that there is no significant increase of m^{6}A level even under 6-hour 150 mM NaCl treatment.

7. In section "ECT8 promotes m^{6}A-modified mRNA decay through direct interaction with DCP5 in P-bodies"

a. Authors use DCP1 to establish that ECT8 localizes in P-bodies. However, every experiment after that uses DCP5. Why?

Response: Thanks for your suggestion. In the revision, we have performed the experiment to show that ECT8 colocalizes with DCP5 in P-bodies (please see Figure 5A). At the beginning, we used DCP1 to confirm ECT8's localization in P-body. After that, we investigated how ECT8 gets into P-body, and we found ECT8 directly interacts with DCP5. Thus, we used DCP5 in every experiment after that. In the

revised manuscript, we have showed ECT8 colocalizes with DCP5 and DCP1 in P-bodies, respectively (Figure 5A and Supplementary Figure S8B).

b. Multiple proteins have been shown to be in P-Bodies, XRN4, VCS, AGO1, etc. The authors did not show if ECT8 interacts exclusively with DCP5 or whether it also interacts with these other proteins? A wider Y2H screen could identify more interactors providing more insight.

Response: Thanks for your suggestion. In the revision, we have conducted an expanded Y2H screening to explore the interaction between ECT8 and a selection of proteins including DCP1, DCP2, DCP5, NOT1, XRN4, VCS, and AGO1. The results showed that ECT8 does not interact with most of these proteins, except for VCS and DCP5 (Supplementary Figure S8C). DCP5, along with associated proteins like VCS, DCP1, and DCP2, primarily functions in removing the protective 5' cap structure from mRNA molecules within P-bodies, ultimately leading to mRNA degradation. The identified interaction between ECT8 with VCS lends further support to the conclusion that ECT8 participates in the degradation process of transcripts at their 5' end within P-bodies.

c. Since AGO1 is also a part of P-bodies, could miRNA mediated mRNA decay also play a part in this? It is known that m^6A also affects miRNA biogenesis (Bhat et al., 2020), how does that affect authors hypothesis.

Response: In the revision, we have performed Y2H assay to show no interaction between ECT8 and AGO1 (Supplementary Figure S8C). It is well known that m^6A methylation occurs in pri-miRNAs and influences miRNA biogenesis in *Arabidopsis*, but no m^6A methylation is present in mature miRNA (Bhat, et al., *Proc Natl Acad Sci U S A*, 2020, 117(35), 21785-95). Considering that ECT8 localizes in the cytoplasm and our FA-CLIP sequencing data reveals that there is no miRNA bound by ECT8 under both mock and salt stress conditions (Additional Table 1). Thus, we think that ECT8 does not function in the biogenesis of miRNA or miRNA-mediated mRNA decay.

Annotation of ECT8 targets (Mock)		Annotation of ECT8 targets (Salt)	
Transcript type	Targets numbder	Transcript type	Targets numbder
mRNA	7019	mRNA	8543
lncRNA	16	lncRNA	15
ncRNA	15	ncRNA	18
nontranslating CDS	4	nontranslating CDS	8
miRNA	0	miRNA	0
snoRNA	0	snoRNA	1
snRNA	0	snRNA	3
rRNA	0	rRNA	0
tRNA	0	tRNA	0

Additional Table 1. Annotation of ECT8 targeted genes under mock and salt stress. All genes are annotated using Araport11 from TAIR website (https://www.arabidopsis.org/)

8. In section "ECT8 destabilizes negative regulators of salt stress response for enhancing salt stress tolerance" line 352-353: "The results showed that they underwent more rapid degradation in ect8-1 compared to WT under both normal and salt stress conditions" I think the authors have misstated the results. Since the figures show stabilization in ect8 mutant.

Response: Sorry for the mistake. We have changed it into "they underwent slower degradation in *ect8-1* compared to WT under both normal and salt stress conditions".

Reviewer #2 (Comments for the Author):

This article focuses on N^6-methyladenosine (m^6A), the most prevalent mRNA modification in eukaryotes, including plants, and its roles in various biological processes such as plant growth, development, and stress response. It highlights the identification of ECT8 as an m^6A reader protein crucial for the plant's response to salt stress. ECT8 enhances the degradation of target transcripts by interacting with the decapping protein DCP5 within P-bodies, a process particularly important under various environmental stresses. The study observes a significant increase in ECT8 expression under such conditions, and specifically in response to salt stress, both at

the transcript and protein levels. This increase in ECT8 leads to more effective binding to m^{6}A-modified mRNAs, promoting their degradation, especially those negatively regulating salt stress response. The findings demonstrate ECT8's role as a sensor of abiotic stresses, aiding in mRNA decay to maintain homeostasis and improve stress tolerance in plants. These insights contribute to the understanding of epitranscriptomic gene regulation and have potential applications in breeding more resilient crops amid changing environmental conditions.

Believing that the article will be read and will be cited, I have a few remarks for the authors to implement corrections or possibly respond to them.

Response: We express our gratitude to you for your positive remarks and comprehensive evaluation of our work. We have provided specific responses to each of the comments in the following sections.

1) I believe that there is a particular lack in Fig S3 of results that show the preferential localization of ECT8 in the cytoplasm using some bioimaging techniques. Ideally, this should be shown for different plant tissues for comparison.

Response: Owing to time limitation, we have not obtained transgenic materials that stably express ECT8 fused with fluorescent proteins under native promoter yet. Instead we reperformed nuclear-cytoplasmic fractionation experiment to blot ECT8 protein with a newly ordered anti-FLAG antibody which has better specificity. The results clearly showed that ECT8 is primarily localized in the cytoplasm (Supplemental Figure S3C). The transiently expression of ECT8-GFP and P-body marker, DCP1-mCherry and DCP5-mCherry, in the *Arabidopsis* protoplast showed that ECT8 colocalizes with DCP1 and DCP5 in P-bodies with condensates, which is present in cytoplasm (Figure 5A; Supplemental Figure S8B).

2) Please correct the reference to Fig in line 218 of the text (it is Figure E); it should be 3E.

Response: We sincerely apologize for the mislabeling of the figures. The error has been revised, and the correct label is now Figure 3E.

3) The image in Figure 5A should include the localization of the cell nucleus. This is a note in addition to point 1. Regarding this image, a better marker for P bodies would be DCP2 or of course DCP5, if the authors have the possibility to show these colocalizations with ECT8. However, if it is not feasible to make this correction to the manuscript within the necessary timeframe, the localization of DCP1 is acceptable.

Response: Thanks for the suggestion. In the revision we have transiently expressed ECT8-GFP and P-body marker DCP5-mCherry in the *Arabidopsis* protoplast and showed that ECT8 colocalizes with DCP5 in the puncta (Figure 5A), further reinforcing the evidence of ECT8's localization in P-bodies.

4) In the diagram (Figure 7) summarizing the study results, the site where mRNA degradation occurs through the interaction of ECT8 with DCP5 is indicated as P bodies. This is likely, but since the authors have not presented direct evidence of such localization of this process in P bodies, at this stage of the research it is only a hypothesis, and this should at least be mentioned in the figure description.

Response: Thanks for the suggestion. In the revision, we redraw the diagram (Figure 7), and stated it is hypothesis in text: "Considering that the number of P-bodies increases in *Arabidopsis* under salt stress conditions (Steffens, et al., *PLoS Biol*, 2015, 13(7): e1002188), we hypothesize that the increased abundance of ECT8 induced by salt stress recruits more m^{6}A-modified mRNAs to get into more P-bodies. As a consequence, this amplifies the degradation of ECT8 bound mRNAs, including negative regulators of salt stress response, ultimately enhancing salt stress tolerance."

5) It's not required, but I really feel the absence of an experiment to find protein partners of ECT8. I am referring to the proteomic analysis of immunoprecipitates (IP-LC-MS/MS). This would provide additional confirmation for DCP5 beyond the Yeast two-hybrid (Y2H) assay, as well as addressing what the authors discuss in lines 420-423.

Response: Thanks for your kind suggestion. In fact, we have conducted protein

IP-LC-MS/MS experiments on ECT8-FLAG/*ect8-1* materials. Due to the low protein level of DCP5, only a small amount of DCP5 protein peptides were detected in our IP group - two and five peptides belonging to DCP5 protein were identified in the two biological replicates, respectively. Thus, we only put Y2H and BiFC results that confirming the interaction between DCP5 and ECT8 in the manuscript.

Reviewer #3 (Comments for the Author):

The manuscript by Cai, Tang and Song et al. reports the function of the m^6A reader ECT8 in Arabidopsis. The authors demonstrate ECT8 plays a role in salt stress tolerance and contributes to mRNA decay through its interaction with the decapping activator DCP5. Overall, I find the work interesting and novel, with genetic evidence and a series of genome-wide omics data to support the authors' conclusion. The study is expansive and provides new findings related to the regulation of mRNA fates by an m^6A reader.

Please see below my specific comments and questions intended to improve the quality of the manuscript.

Response: We're truly thankful for your kind comments and detailed review of our work. The specific, point-by-point responses to your comments are addressed below.

Major comments:

1. Western blot images of FLAG-tagged ECT8

The western blot images of FLAG-tagged ECT8 (Figure 2B) showed two discreet bands with anti-FLAG detection. What would be an explanation of for this phenomenon? This is consistent with the data in Supplemental Figure S3C. it seems to me that the total protein fraction showed ECT8 detection with two sizes, while the cytosolic fraction contained the band of smaller size and the nuclear fraction with the larger size. I am not convinced that ECT8 is preferentially cytosolic. The anti-FLAG panel of Supplemental Figure S3C should be cropped in a way that the upper band is not omitted.

Response: Thanks for your kind suggestion. We appreciate your observation regarding the ECT8 protein bands. Upon comparing with other wild-type plant samples, we identified that the upper bands corresponding to ECT8 in our initial submission was caused by non-specific binding of the *Arabidopsis* protein with our used anti-FLAG antibody. Therefore, to reduce the influence of non-specific protein bands in western blotting assay, we have purchased a different anti-FLAG antibody (MCE, Catalog#: HY-P80111) and observed a clear and single band of ECT8 around the 65 kDa (Figure 2C; Supplemental Figure S3C).

Additionally, we have also repeated the time-course experiment to track the protein expression level of ECT8 under 150 mM NaCl stress using new antibody (Figure 2C). Our results reveal that the protein level of ECT8 increases during salt stress.

2. Comparison of ECT8-binding sites and m^{6}A peaks

The authors compared m^{6}A-seq with FA-CLIP data under both mock (control) and salt stress conditions. This showed that the majority of ECT8 targets have m^{6}A marks, which makes sense. However, I find the number labeling format for Figures 3A and 3B confusing. I don't understand why there were two numbers for the overlaping region of the Venn diagrams. In Figure 3A, for example, it would be understandable if there were 16,177 m6A targets and 7,592 ECT8 targets detected, with the overlap of 5,656. But the authors also labelled 5,684 m^{6}A targets and 6,841 ECT8 targets in the overlapping region that do not add up to 5,656. Please clarify why this was the case. More explanation in the text and/or figure caption would be helpful.

Response: Sorry for the confusion. In the revision, we have changed "ECT8 & m^{6}A targets" into "ECT8 & m^{6}A targeted genes" to make clearer and more understandable. We utilized the *bedtools intersect* algorithm to draw the Venn diagrams. In the revision, we have done m^{6}A-seq using triplicated biological samples which makes the little different on the m^{6}A peaks numbers. Figure 3A, for instance, 5,532 m^{6}A peaks were overlapped with ECT8-binding sites, while 6,535 ECT8-binding sites were overlapped with m^{6}A peaks. The number of genes containing the overlapped 5,532

m^{6}A peaks and 6,535 ECT8-binding sites were 5,479, which was named as "ECT8 & m^{6}A targets" in the last version, but now was changed into "ECT8 & m^{6}A targeted genes".

I am also confused why the number of ECT8 & m^{6}A binding sites presented in Figure 3F did not correspond with the number in Figures 3A and 3B. My understanding is that there were 5,656 ECT8 & m6A targets under mock condition and 7,075 under salt stress conditions. However, the number in Figure 3F was 6,841 and 8,119, respectively. Was this because Figures 3A and 3B referred to the number of genes but 3F referred to the number of sites? Whatever the case, the analysis and comparison should be described more clearly in the text and figure captions. The distinction between targets and sites should be clearly defined and presented systematically. Perhaps replacing Figure 3F with Figure S5D would make it easier for the reader to follow.

Response: Sorry for the confusion. As suggested, we have replaced Figure 3F with Figure S5F in the revised manuscript. Here is the explanation about Figure 3A and 3B with the related Figure 3F (Figure S5F in the revision). Figure S5F is overlapping ECT8-binding sites modified with m^{6}A (ECT8 & m^{6}A binding sites) between normal (6,535 sites) and salt condition (8,240 sites). The number of ECT8 & m^{6}A binding sites under normal condition (6,535 sites) is shown in Figure 3A, which shows 6,535 ECT8 binding sites were overlapped with m^{6}A peaks. Similarly, the number of ECT8 & m^{6}A binding sites under salt condition (8,240 sites) is shown in Figure 3B, which shows 8,240 ECT8 binding sites were overlapped with m^{6}A peaks. We have revised the description clearer in the text and figure legends.

By comparing Figure 3A with Figure 3B, there was increase of the ECT8 & m^{6}A targets from 5,656 targets under mock condition to 7,075 under salt condition. Is there an enrichment for specific biological functions from those 1,419 targets that was increased by salt stress?

Response: Thanks for your suggestion. In our manuscript, we conducted a GO analysis on the common ECT8 & m^{6}A targeted genes under both mock and salt stress

conditions (Figure 3I). In detail, our Venn diagram revealed 1,381 ECT8 & m^6A targets specific to the Mock condition (we called mock specific ECT8 & m^6A targeted genes), 3,172 ECT8 & m^6A targets specific to the salt condition (we called salt specific ECT8 & m^6A targeted genes) and 4,098 ECT8 & m^6A targets common to both conditions (we called common ECT8 & m^6A targeted genes) (Figure 3F).

In response to your suggestion, we have taken mock specific ECT8 & m^6A targeted genes and salt specific ECT8 & m^6A targeted genes to conduct GO analysis (Supplementary Figure S5, I and J in this revision). The results were obtained using DAVID program (https://david.ncifcrf.gov/tools.jsp) and further visualized using R packages *ggplot2*. It is evident that the mock-specific, common, and salt-specific ECT8 & m^6A targeted genes are all enriched in pathways related to gene expression regulation and RNA metabolism. Interestingly, we observed a distinct enrichment in the GO pathways related to osmotic changes and salt stress response in both common and salt-specific ECT8 & m^6A targeted genes.

The color scheme for Figure 3F should be changed from blue-green to blue-red as in Figure 2C, as this was a comparison of the mock and salt stress conditions. I understand that GO enrichment analysis was performed on ECT8 & m^6A common (mock VS control) targets and presented in Figure 3I, but what about those that are unique to either mock or salt stress conditions?

Response: Thanks for the suggestion. We have changed the color of Figure 3F (Figure S5F in the revised manuscript) into blue-red. As responded in last question, we have performed GO analysis for the unique ECT8 & m^6A targeted genes specific to mock and salt, please see Supplementary Figure S5, I and J in the revision.

Lines 224-228: The author wrote, "It has been observed that under salt stress, ECT8 exhibits a significantly increased binding affinity to ECT8 & m^6A common targets, even in the absence of a substantial increase in overall m^6A abundance or the expression levels of m^6A writer subunits and erasers (Figure 3, G and H; Supplemental Figure S4, B and C; Supplemental Figure S5E)." In fact, Figure 3H

indicates that m^6A level was significantly decreased for ECT8 & m^6A common targets under salt stress conditions. The text should be corrected accordingly.

Response: In the revision, we performed m^6A-seq using triplicated biological samples and employed m^6A spike-in to calculate the m^6A levels of common ECT8 & m^6A targeted genes (mock & salt) following the analysis method for m^6A level used in the literature (Wei et al., *Science*, 2022, 376(6596), 968-73). The results showed that the m^6A level of common ECT8 & m^6A targeted genes were not significantly altered upon 4-hour salt treatment compared to normal (Figure 3H). Furthermore, our mass spectrometry analysis (Supplemental Figure S5H) confirmed that there was no significant increase in the overall m^6A levels pre and post salt stress treatment, consistent with the findings of other studies (Hu et al., *Plant J*, 2021, 106: 1759-75), which also reported that there is no significant increase of m^6A level even under 6-hour 150 mM NaCl treatment. Thus, in the revised manuscript we have changed the sentence into "ECT8 exhibits a significantly increased binding ability towards common ECT8 & m^6A targeted genes under salt stress condition compared to normal condition, although overall m^6A abundance varies negligibly between both conditions".

3. mRNA lifetime sequencing & ribosome-sequencing

There was no indication on how many replicate samples were used for mRNA half-life sequencing, and what the statistical model/criterion was used to compare the mRNA half-life between the genotypes. This makes it impossible to determined the quality of the mRNA decay data and whether the authors' interpretation of the data was valid. The data in Figure 4B were presented as cumulative fraction of relative mRNA half-live between ect8-1 mutant and the wild type, without any other information provided to allow for an assessment of the efficiency of cordycepin treatment. What is the number of seedlings used in the cordycepin treatment experiment? Also the libraries was made with poly(A) selection, which overlooked mRNA decay intermediates without poly(A) tails.

Response: We have added the detailed information in the method of "mRNA lifetime

sequencing”. In brief, we organized this experiment into five groups, each representing a different time point. Each group consisted 7-d-old WT and *ect8-1* seedlings (ten plants for each genotype). We conducted two biological replicates for mRNA lifetime sequencing and ensure the reliability of our sequencing data (Supplemental Figure S6B).

Regarding the efficiency of our cordycepin treatment, we observed that the median mRNA half-life for WT in our study is approximately 101 minutes (Additional Figure 1A). This finding aligns closely with previously reported data that shows 107 minutes of the median mRNA half-life for WT (Sorenson et al., *Proc Natl Acad Sci U S A*, 2018, 115(7), E1485-94). Moreover, we selected two typical known transcripts - *AT5G46020* with a long half-life and *AT2G41010* with a short half-life - for evaluating the quality of our sequencing data. Our results exhibit the mRNA half-life features of these two transcripts (Additional Figure 1B). This evaluation allows us to validate the feasibility of our procedure for measuring mRNA half-life. Consequently, we have confidence in the effectiveness of our cordycepin treatment approach.

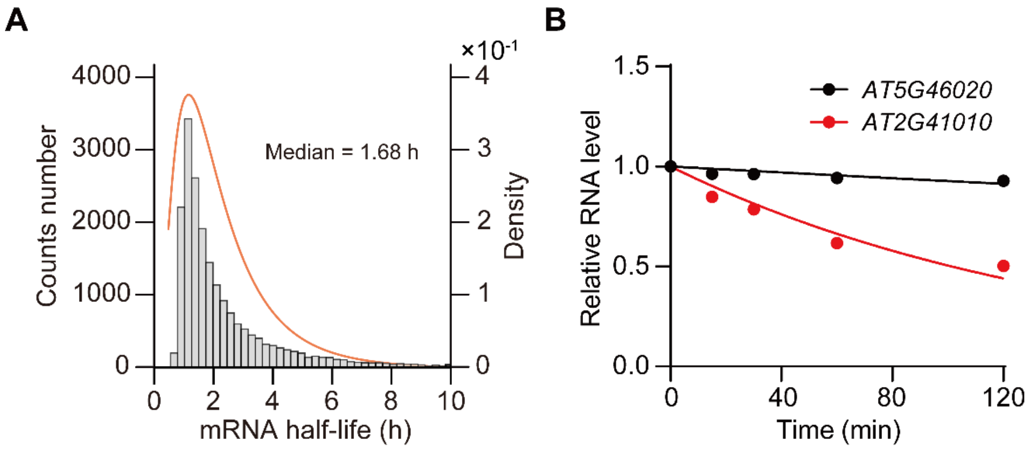

Additional Figure 1. (A) The distribution of mRNA half-life in WT; (B) The degradation curve from two typical transcripts.

We appreciate your point about the poly(A) selection during library preparation, which could potentially overlook mRNA decay intermediates without poly(A) tails.

However, our primary focus in this study was on the mRNAs, which represent the majority of ECT8 targeted genes (Supplemental Figure S5C). Nonetheless, we acknowledge that whether ECT8 influences the decay rate of transcripts without poly(A) tails is also important, and we will consider this in our future investigations. Thank you again for your valuable feedback.

This was also the case for the quality of ribosome-sea data. The authors made a conclusion that ECT8 mutation has no effects on translation efficiency of ECT8 & m^{6}A targets, but without providing sufficient evident that the conclusion was based on high quality ribo-seq data.

Response: In response to your concern, we have thoroughly examined our ribosome-seq data using heatmaps that clearly demonstrate the consistency and reliability of our data (Supplemental Figure S7B). The heatmaps, we believe, provide substantial evidence to support our conclusion that ECT8 does not significantly affect the overall translation efficiency under normal condition. We appreciate your feedback and hope that this addresses your concern.

To support the claim made in Figure 4 and demonstrate that the increase in mRNA abundance of ECT8 & m^{6}A targets in the ect8-1 mutant is specifically due to decreased decay rates without an increased transcription, perhaps quantification of pre-mRNA could be made for some representative genes. It would be more convincing if there was no change in pre-mRNA detected between the two genotypes while the total poly(A)$^+$ mRNA levels were increased in ect8-1 mutant.

Response: Thanks for your suggestion. In the revision, we have RT-qPCR to assess the expression levels of pre-mRNA of the representative ECT8's targeted genes (*PPRT1*, *MSI1*, *BIL1*, *ENGD1*). Our findings indicate that the absence of ECT8 does not significantly affect the abundance of pre-mRNA of these genes under both normal and salt conditions (Supplemental Figure S10).

4. Localization of ECT8 in processing bodies of visible sizes

The conclusion that ECT8 is localized to processing bodies was drawn from transient over-expression of ECT8-GFP in the protoplast system, which does not reflect the physiological ECT8 concentration within the cells. I wondered if this would still be the case if the protein is stably expressed under its endogenous promoter.

Response: We appreciate your comment regarding the subcellular localization of ECT8 in P-bodies. Due to the time limitation, we have not obtained transgenic materials that stably express ECT8 fused with fluorescent proteins under native promoter yet. Instead, we have transiently expressed ECT8-GFP and P-body marker DCP5-mCherry in *Arabidopsis* protoplast and showed that ECT8 colocalizes with DCP5 in the puncta (Figure 5A), further reinforcing the evidence of ECT8's localization in P-bodies. We also perform a expand YTH screen to find ECT8 not only interacts with DCP5 but also VCS (Supplemental Figure 8C). DCP5, along with associated proteins like VCS, DCP1, and DCP2, primarily functions in removing the protective 5' cap structure from mRNA molecules for degradation within P-bodies (Xu, et al., *Plant Cell*, 2009, 21(10), 3270-9). The identified interaction between ECT8 with VCS lends further support to the conclusion that ECT8 participates in the degradation process of transcripts at their 5' end within P-bodies.

5. The oligo primers sequences used for cloning and generation of transgenic lines should have been provided.

Response: We apologize for this omission. Complete primer details have now been added to Supplemental Table 1.

Minor comments:

1. Figures 1D and 1E: how was statistical analysis performed with two biological replicates?

Response: Data in (D) and (E) are analyzed by two-sided t test and presented as means ± SE, n = 3 biological replicates × 3 technical replicates, $^{***}P < 0.001$ and $^{****}P < 0.0001$. We have added it in the legend of Figure 1.

2. For Supplemental Figure S1A, in addition to the conserved region with 3 or 4 tryptophan residues, a diagram comparing the protein domains/motifs would provide some insight about the proteins under comparison.

Response: Thank you for your suggestion. In the revised manuscript, we have compared the YTH domains among ECT8, ECT2, and human YTHDF1-3, please see Supplemental Figure S1A.

Line 138: Supplemental Figure S2, A-C, not Supplemental Figure S1, A-C. Line 139: Supplemental Figure S2D, not Supplemental Figure S1D. It would be helpful to the reader if the location of genotyping primers used for Figure S2D was demonstrated in the diagram of Figure s2A.

Response: We appreciate your attention to detail and all these errors are corrected. We also illustrated the location of primers used for genotyping in Supplemental Figure S2A.

Line 218: Figure 4E

Line 449: transcribed

Response: All these have been corrected.

Line 508: Would it be more appropriate to use poly(A)+ RNA instead of mRNA? There was no information on how total RNA isolation and polyA selection were performed and what tissue was used.

Response: Thanks for the suggestion. We have changed mRNA into poly(A)$^+$ RNA. The method related with total RNA extraction and poly(A)$^+$ RNA isolation was in the method of “poly(A)$^+$ RNA-seq”. In brief, total RNA was extracted from *Arabidopsis* seedlings using TRIzol™ Reagent (Invitrogen). Subsequently, total RNA was then employed to isolate poly(A)$^+$ RNA using oligo(dT)$_{25}$ Dynabeads (Thermo Fisher Scientific) follow by the procedure.

Line 548: at the same time

Response: Sorry for this mistake, and it has been corrected now.

Line 550: presented, typo?

Response: It has been corrected in our revised manuscript.

Line 557: (1.54d), what does this mean?

Response: In this context, '(1.54d)' indicates that the version 1.54d of ImageJ was used. We have changed it into "version 1.54d".

Line 566: No DNAse treatment before cDNA synthesis?

Response: Thanks for your query regarding our cDNA synthesis process. We used the PrimeScript™ RT Reagent Kit with gDNA Eraser (Perfect Real Time) (RR047A) from TaKaRa for our experiments. The protocol for this kit begins with the treatment of 1μg of total RNA with gDNA eraser to remove any potential DNA contamination. The subsequent reverse transcription process was also conducted following the protocol provided by the kit. The resulting cDNA was appropriately diluted before being used for qPCR analysis. We have added the information into the method of "RT-qPCR".

Line 600: after that?

Response: It has been revised.

Line 608: poly(A)+ RNA-seq: please provide more information on how sequencing of the RNA-seq libraries was performed.

Response: We have added the detailed information into the related methods. All the libraries of RNA-seq, m^6A-seq, FA-CLIP and Ribo-seq were sequenced on the Illumina NovaSeq 6000 platform with a paired-end model (PE150).

2nd Accepted Version - Editorial Decision 11-Apr-2024

Dear Dr. Guifang Jia:

We are pleased to inform you that your paper entitled "m6A reader ECT8 functions as an abiotic stress sensor by accelerating mRNA decay in Arabidopsis" has been accepted for publication in The Plant Cell, pending a final editorial review by a science editor. At this stage, your manuscript will be evaluated by a science editor with respect to its presentation of scientific content, compliance with journal policies, and presentation for a broad readership. The Plant Cell has contracted with Plant Editors (planteditors.com) to provide this service to our authors, and you will soon receive additional information on this process.

Please note that each author needs to link their ORCID identifier to their account in the system before your manuscript can be published. If any authors do not have an ORCID linked to their account, they will receive a message with a link to complete this task. Please ensure that ALL of your coauthors have completed this task as soon as possible.

ASPB offers an OPEN option that allows authors to have their online articles available for free to all users immediately upon publication. For more information about the ASPB OPEN option, refer to the Final Submission Checklist Form.

The Plant Cell and The Arabidopsis Information Resource (TAIR) are collaborating to collect functional annotation data about Arabidopsis genes from authors. This includes information about the gene's molecular function (e.g., kinase activity, ATP synthetase activity), the biological process/es it is involved in (e.g., endosperm development, threonine biosynthesis), its subcellular location (e.g., nucleus, ER), anatomical or developmental expression pattern (e.g., leaf, ovule, flower stage 10, seedling stage), or its partner in a protein-protein interaction (e.g., AT1G01010 interacts with AT1G01020).

If your paper contains results falling into one or more of these categories for Arabidopsis genes, we request that you now submit these data for inclusion in TAIR by filling in the form provided at the following URL: https://www.arabidopsis.org/doc/submit/functional_annotation/123. If you need further clarification on what types of data can be submitted please contact curator@arabidopsis.org.

Finally, we encourage your submission of artwork for the journal cover. Monthly issues will have an online cover image and selected covers will be used for posters, other promotional items, and "wallpaper" for mobile devices. For more information, refer to Cover Submission in the Instructions for Authors https://tpc.msubmit.net/cgi-bin/main.plex?form_type=display_auth_instructions.

We look forward to seeing your paper published.

Sincerely,

The Plant Cell Editorial Board

=====
IMPORTANT REMINDER: PEER REVIEW REPORTS
=====

If you opted to publish a peer review report along with your article during the original submission process, it will be prepared by the editorial staff and publicly posted with your manuscript, inside the zip file that contains any other supplemental material. As a reminder, the peer review report is a public record of all comments from editors and reviewers, as well as your prior responses, as you received them in the decision letters for each draft of your manuscript. If you agreed to publish this report and have changed your mind, or are not sure if you selected this option, please contact the editorial office as soon as possible before signing the license agreement from our publisher.

----FOR ASPB OFFICE USE ONLY (DO NOT EDIT)----
MSID: 37378
Corresponding Author: Prof. Dr. Jia
